# Antibiotic resistance genes are differentially mobilized according to resistance mechanism

**DOI:** 10.1093/gigascience/giac072

**Published:** 2022-07-30

**Authors:** Tue Kjærgaard Nielsen, Patrick Denis Browne, Lars Hestbjerg Hansen

**Affiliations:** Department of Plant and Environmental Sciences, Section for Environmental Microbiology and Biotechnology, University of Copenhagen, Thorvaldsensvej 40, Frederiksberg C 1871, Denmark; Department of Plant and Environmental Sciences, Section for Environmental Microbiology and Biotechnology, University of Copenhagen, Thorvaldsensvej 40, Frederiksberg C 1871, Denmark; Department of Plant and Environmental Sciences, Section for Environmental Microbiology and Biotechnology, University of Copenhagen, Thorvaldsensvej 40, Frederiksberg C 1871, Denmark

**Keywords:** antibiotic resistance genes, mobile genetic elements, bioinformatics

## Abstract

**Background:**

Screening for antibiotic resistance genes (ARGs) in especially environmental samples with (meta)genomic sequencing is associated with false-positive predictions of phenotypic resistance. This stems from the fact that most acquired ARGs require being overexpressed before conferring resistance, which is often caused by decontextualization of putative ARGs by mobile genetic elements (MGEs). Consequent overexpression of ARGs can be caused by strong promoters often present in insertion sequence (IS) elements and integrons and the copy number effect of plasmids, which may contribute to high expression of accessory genes.

**Results:**

Here, we screen all complete bacterial RefSeq genomes for ARGs. The genetic contexts of detected ARGs are investigated for IS elements, integrons, plasmids, and phylogenetic dispersion. The ARG-MOB scale is proposed, which indicates how mobilized detected ARGs are in bacterial genomes. It is concluded that antibiotic efflux genes are rarely mobilized and even 80% of β-lactamases have never, or very rarely, been mobilized in the 15,790 studied genomes. However, some ARGs are indeed mobilized and co-occur with IS elements, plasmids, and integrons.

**Conclusions:**

In this study, ARGs in all complete bacterial genomes are classified by their association with MGEs, using the proposed ARG-MOB scale. These results have consequences for the design and interpretation of studies screening for resistance determinants, as mobilized ARGs pose a more concrete risk to human health. An interactive table of all results is provided for future studies targeting highly mobilized ARGs.

## Background

Pathogenic bacteria resistant to antibiotics pose an enormous threat to human health, resulting in up to 10 million annual deaths in 2050 if we do not address the issue now, as estimated by the UN Interagency Coordination Group on Antimicrobial Resistance [1]. Health and environmental challenges imposed by antibiotic resistance have sparked enormous research efforts into characterizing genetic resistance determinants. Combined with broad availability of second- and third-generation sequencing technologies, studying the presence and prevalence of antibiotic resistance genes (ARGs) in the environment has become popular in recent years. Bacteria can become resistant to antibiotics through several genotypic changes, including point mutations that lead to either altered gene expression or change of protein function, gene amplifications, genome shuffling leading to increased expression of resistance determinants, and lastly through acquisition of novel genetic material by horizontal gene transfer. The latter transferral of ARGs is especially problematic as, for example, plasmids carrying ARGs with strong adjacent promoters can be globally spread to important human pathogens. Here, we focus on the genetic context of these acquired resistance determinants and evaluate to what degree they have been mobilized.

Antibiotic resistance remains a significant global issue despite numerous studies into understanding the spread of genes via mobile genetic elements [2, 3] and devising mitigation strategies. However, many classes of ARGs are intrinsic to bacterial genomes and can be considered part of the core bacterial genome and may perform nonresistance functions [4–7]. Furthermore, many ARGs have only been shown to provide resistance when cloned into expression vectors [8, 9] or with constitutive expression in mutants. Some of these cloned ARGs can hypothetically be transiently highly expressed to confer some level of resistance in their native genetic context or, as mentioned above, have mutations in a controlling promoter or repressor gene, leading to high expression. Alternatively, ARGs can become “decontextualized” by mobilization, leading to overexpression and resistance [6, 10–14], which moreover can lead to the spread of the decontextualized ARGs via horizontal gene transfer. Examples of genetic events that can lead to mobilization and dissemination of ARGs include (i) insertion of a proximal insertion sequence (IS) element with an internal promoter [15] that may lead to the formation of a unit or composite transposon that can carry an ARG as an accessory gene, (ii) subsequent transfer from chromosomes to (high copy number) plasmids [16], (iii) capture by and integration within integrative and conjugative elements (ICEs) [17], and (iv) incorporation of an ARG into an integron as a gene cassette [18, 19]. This aspect is often overlooked in culture-independent studies using (meta)genomics and/or quantitative polymerase chain reaction (PCR)–based detection where resistance is rarely experimentally verified. The issue is further discussed in a review by Martínez et al. [14]. Thus, screening the environment for ARGs may give the impression that “resistance is everywhere” or that widespread resistance predates the use of antibiotics [20–22], although the native roles of ARGs’ homologs may not be related to antibiotic resistance [5, 10, 23–27]. This topic is subject to debate, with some researchers claiming that all ARGs are suitable targets to screen for in, for example, metagenomes, since they can be potentially decontextualized and ultimately lead to problematic resistance in pathogens [28] and others warranting that ARGs should be ranked according to how much of a concrete risk they pose [23].

When coupling (meta)genomic predictions with culture-based detection of resistant strains, it is often found that the two approaches do not agree [8, 29–32], partially attributable to the fact that gene expression is rarely considered [33]. Screening a genome for resistance markers against an ARG database sometimes results in copious false-positive resistance predictions [30]. This issue can be most pronounced for efflux-related markers where the specificity of prediction has been reported to be 0.12 [34]. The balanced accuracy of resistance marker prediction against two widely used databases was only 0.52 and 0.66, showing that finding ARGs does not necessarily equate to phenotypic resistance [34]. As discussed in a EUCAST report, the resistance genotype–phenotype concordance has elsewhere been shown to be much higher, with agreements reaching almost 100% in studies that apply subsets of focused and manually curated ARG databases to predict resistance toward selected antibiotics in well-studied species with clinical relevance [35]. In the same report, it is argued that high genotype–phenotype concordances mostly apply to well-characterized (clinical) isolates [35], emphasizing the potential problems in applying large ARG databases to screen for resistance in environmental samples and in less well-studied species. Researchers have devised more advanced machine learning prediction methods to predict resistance in well-characterized bacteria such as *Mycobacterium tuberculosis* with a specificity and sensitivity ranging from 82% to 92.7% [36], in nontyphoidal *Salmonella* with an accuracy of 95% [37], and in *Escherichia coli* with an average accuracy of 91% [38]. These examples highlight that accurate resistance predictions can be made on well-characterized bacteria using curated subsets of well-understood ARGs, but also that predictions on less characterized taxa and in environmental metagenomics using large and unspecific ARG databases are subject to be erroneous.

Although low specificity of resistance prediction from efflux-based ARGs has been reported [34], it should be noted that many groups of efflux pumps are inherently encoded, unmobilized, on chromosomes of important human pathogens where they can confer intrinsic resistance, potentially along with other nonresistance functions [7, 39]. They may be transiently strongly expressed to confer resistance or overexpressed through mutations in transcriptional regulators [39, 40]. The pathogen *Acinetobacter baumannii* serves as a great example of bacteria that host a wide range of efflux pumps, of which some confer intrinsic resistance and others require overexpression. Many of the native *A. baumannii* pumps are involved in nonresistance functions such as membrane composition and stability, opaque/translucent colony phase variation, various stress reliefs, biofilm formation, plasmid transfer rates, natural transformation, quorum sensing, and efflux of dyes, disinfectants, metals, and other nonantibiotic compounds [39]. Expression of efflux pumps is usually under the regulation of either local and/or global transcriptional regulators, and mutations in these regulators are an important path to phenotypic resistance through overexpression in clinical isolates [41]. Besides transient changes in expression or mutations in transcriptional regulator genes, the mobilization of ARGs is an essential aspect of phenotypic resistance.

Most ARGs likely have native roles unrelated to resistance to clinical concentrations of antibiotics [23]. Many antibiotics are natural secondary metabolites, occurring at clinically insignificant concentrations, that are involved in intercellular communication [42], regulation of metabolism, and other nonresistance functions [29]. ARGs have been found in and cloned from susceptible bacteria [8], where they are simply performing their original nonresistance roles. Previous studies have shown that, for example, efflux pumps [10, 24], β-lactamases [25, 26], and lipid A modifying proteins (MCR) [27] have nonresistance functions, although the genes encoding these may be decontextualized to confer resistance.

Functional (meta)genomic approaches, essentially cloning fragmented DNA into expression vectors followed by screening for antibiotic resistance [4, 9, 21, 22, 43, 44], has led to the identification of many putative ARGs [44–50]. Such genes are decontextualized in the experimental setup, and their native roles may not be related to resistance. This has resulted in a problematic dissemination of self-reinforcing resistance-related annotations in gene databases. Thus, sequence homology is a poor proxy for resistance, and culture-independent techniques will often yield misleading results if genetic contexts of ARGs are not considered. With recent advances in long-read sequencing, high-quality metagenome-assembled genomes can be derived [51], facilitating consideration of the genetic context of ARGs.

The associations between ARGs and mobile genetic elements (MGEs) are important [16, 52, 53] and have profound effects on phenotypic resistance [11, 18, 54–56]. It has been argued, for example, in the “RESCon” framework [57] that multiple aspects, including genetic context, should be included in risk assessment of ARGs [58, 59]. Here, we initiate the route to more accurate ARG predictions by categorizing associations between ARGs and MGEs in all completed RefSeq bacterial genomes. We hypothesize that highly mobilized classes of ARGs represent those that are infamous for causing phenotypic resistance and were furthermore initially characterized from already resistant clinical isolates. On the other hand, we also hypothesize that ARGs with a low degree of mobilization are represented by ARG classes that were initially identified through shotgun cloning from nonresistant isolates and subsequently only shown to confer resistance through overexpression from a plasmid vector. This does not rule out future mobilization events and subsequent elevation of the risk posed by a yet unmobilized ARG.

Decontextualization of ARGs is explored by examining their association with (i) plasmids, (ii) IS elements, (iii) integrons, and (iv) their dispersal across distinct genera. We collect this information per class of ARG in the unifying ARG-MOB scale for mobilization of resistance genes in all complete RefSeq bacterial genomes. Among other results, we conclude that most classes of antibiotic efflux genes are rarely mobilized from their original, chromosomal location and that even 80% of classes of β-lactamases have never or very rarely been mobilized. This necessitates both increased awareness of the genetic context of ARGs but also more critical choosing of ARG targets for future, especially environmental, studies.

## Data Description

**Fig. 1:**
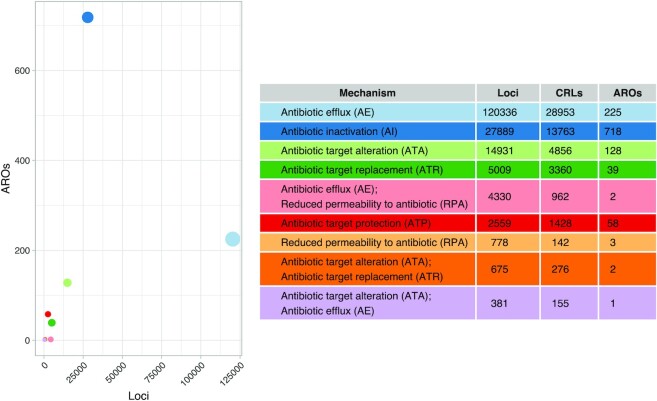
Overview of counts of ARG loci, clustered resistance loci (CRLs), and CARD Antibiotic Resistance Ontology (ARO) terms. In the plot, points are sized according to their CRL count (see table). Row colors in table correspond to their point colors in the left plot. The three hybrid mechanisms AE/RPA, ATA/ATR, and ATA/AE, as well as the low ARO count RPA mechanism, are excluded from most analyses, as they are here not considered “main mechanisms.”

**Fig. 2:**
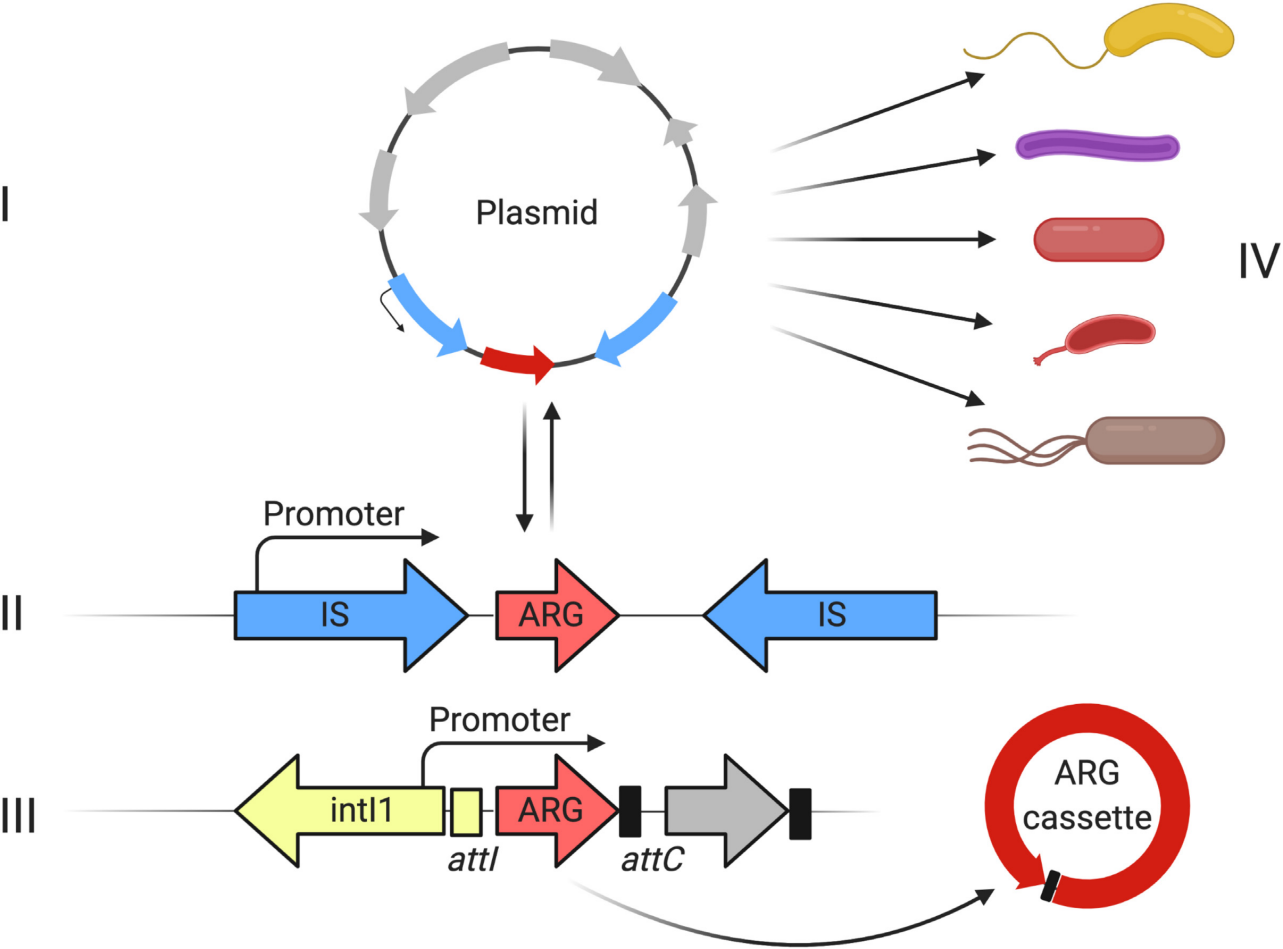
The four investigated mobilization parameters. (I) classification of the replicon type that an ARG loci was found on, (II) presence of one or more IS elements within 12,170 bp either up- or downstream of ARG, (III) association of found ARG with integrons, and (IV) the phylogenetic spread across genera, calculated by the Simpson diversity index. ARGs residing on plasmids can be rapidly spread horizontally and, in the case of multicopy plasmids, may be under heterologous expression. Many IS elements have an internal promoter that can overexpress accessory genes or may contain an outward-facing -35 component that can form a hybrid promoter, if the IS element is inserted close to a -10 box. If inserted as a gene cassette in an integron, the ARG is likely to be overexpressed by the common integron promoter. Furthermore, a gene cassette containing an ARG may form circular DNA molecules from the integron cassette array that can be shuffled to other locations. The final factor considered in this study with regards to mobilization of ARGs is the already observed phylogenetic dispersal of said ARGs across the genera represented in the RefSeq complete genomes database.

The CARD database was used to find ARGs in all completed bacterial genomes from the RefSeq database (*n* = 15,790). Then, 12,170 bp up- and downstream of predicted ARGs were analyzed for IS elements and integrons, while replicon type (plasmid or chromosome) was also considered. For more details, see Methods and Supplementary Information (Supplementary Text 1, Supplementary Figs. S1–S4; Supplementary Table S1). All databases are assumed to be biased, especially toward human-associated bacteria, of which many almost identical genomes have been uploaded to RefSeq, leading to overrepresentation of these compared to, for example, environmental bacteria (Supplementary Text 2, Supplementary Figs. S5 and S6). In order to ameliorate these biases, highly similar genetic loci with predicted ARGs (*n* = 176,888) were clustered to 53,895 clustered resistance loci (CRLs), representing 1,176 Antibiotic Resistance Ontology (ARO) terms from CARD (Fig. 1). We compared the frequency of genera found in both CARD and RefSeq and calculated the Euclidean distance of these frequencies before and after clustering to CRLs. The Euclidean distance of genera frequencies was reduced from 30.89 to 10.26, showing that many ARG loci in RefSeq are highly similar (Supplementary Text 3, Supplementary Fig. S7). Four mobilization parameter ratios were explored for each CRL (Fig. 2): (i) replicon type, (ii) IS element association, (iii) integron association, and (iv) dispersal of CRLs across genera (Simpson diversity). All parameters were calculated on a scale of 0–1, with 1 indicating that a CRL is always associated with the given parameter. The mean of the four ratios is termed the ARG-MOB score and indicates how much genes of a given ARO are mobilized. This is described further in later sections and Methods (Supplementary Fig. S8). Prophages in genomes are not explored for ARGs, since these are not common vectors [60]. Neither are ICEs explored, although they are important in resistance development [17], since they are underexplored and likely difficult to predict across the wide phylogenetic array of genomes studied here. Likewise, bacteriophages represent an astounding reservoir of genetic diversity [61], and successful prediction of integrated prophages is likely extremely limited to those occurring in well-studied bacterial families. Including ICEs and prophages here would introduce severe biases in the analyses and they are thus excluded.

## Analyses

### The 16S ribosomal RNA gene as a nonmobilized control

The average length of 449 composite and unit transposons in The Transposon Registry [62] was calculated to 12.17 kbp (Supplementary Fig. S4). This distance was used to screen for the presence of IS elements and integrons in both directions from identified ARGs. The 16S ribosomal RNA (rRNA) gene was used as a nonmobilized control, as this gene is not expected to be associated with MGEs (Supplementary Text 4). Only 5.59% of 16S rRNA genes in the 15,790 complete genomes are within 12.17 kbp of an IS element (Supplementary Fig. S9). Since 16S rRNA genes should be extremely rarely associated with, for example, transposons, we consider the 5.59% as a proxy confidence interval for false-positive ARG–IS associations.

### Efflux-associated ARG loci are less unique than other mechanisms

Loci with efflux-associated ARGs were more compressed by clustering to CRLs than all other types (Mann–Whitney *U*-test [MWU] *P*_adj_ < 0.0001; Supplementary Fig. S7), indicating that these are more conserved and contain less variation from mobilization events, for example. The *antibiotic efflux* (*efflux*) mechanism is the most abundant category and its CRL count is more than two times more numerous than the second largest category, *antibiotic inactivation* (*inactivation*), although *inactivation* has over three times as many AROs as *efflux* (Fig. 1). As expected, loci in human-associated genera were especially compressed by clustering, showing that these are indeed overrepresented in the RefSeq database (Supplementary Fig. S7).

### Association of ARGs with IS elements and plasmids

**Fig. 3:**
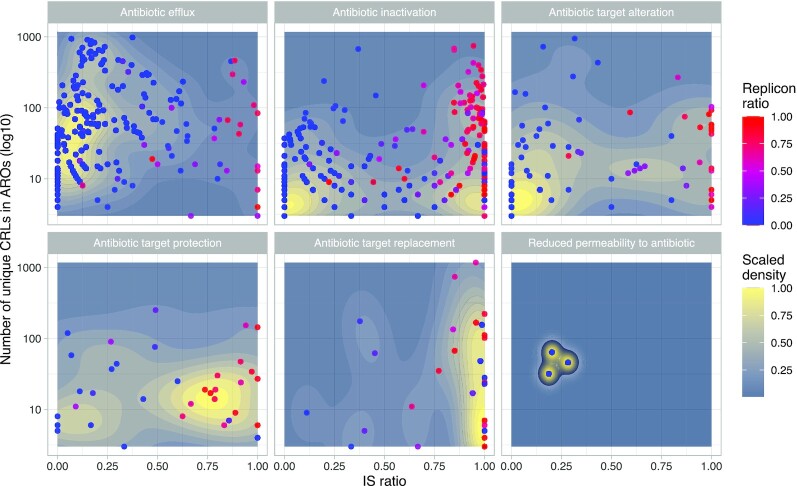
Density plots of IS ratio against the number of unique clustered CRLs in a given ARO category, represented by individual points. Plots are divided into the individual mechanisms and are colored according to the replicon ratio, where a high ratio (red) indicates that an ARO is more often found on plasmids and a low ratio (blue) indicates that an ARO is more on chromosomes. Density estimates are calculated with 2-dimensional kernel density estimation, as implemented in the stat_density_2d function under the ggplot R package. The hybrid mechanisms are not included.

Major resistance mechanisms (nonhybrid) are associated with IS elements and plasmids to varying degrees (Fig. 3) and were associated with different families of IS elements (Supplementary Text 5, Supplementary Fig. S10). *Efflux* AROs generally have very low IS and replicon ratios, which indicates that *efflux* ARGs are rarely mobilized by either IS elements or plasmids. Only few *efflux* AROs have both high IS and high replicon ratios, including ARO3002693 (transposon-encoded *cmlA1* chloramphenicol exporter), ARO3003836 (*qacH* subunit of fluoroquinolone exporter), and ARO3000165 (tetracycline efflux pump *tetA*). Many *efflux* AROs contain a large number of unique CRLs, as is also reflected by *efflux* CRL count in Fig. 1. Therefore, *efflux* ARGs are rarely associated with either IS elements or plasmids. Distances, in terms of nucleotides, between ARGs and IS elements are larger for *efflux* AROs than for other mechanisms, indicating that *efflux* ARGs are more “loosely” associated with IS elements than other mechanisms (MWU test *P*_adj_ < 0.014; Supplementary Text 6, Supplementary Fig. S11). Contrary to *efflux*, the *inactivation* mechanism has many AROs that have been mobilized by both IS elements and plasmids but also AROs that are hardly mobilized at all (Fig. 3). With some exceptions, *antibiotic target alteration* (*target alteration*) AROs have low IS and replicon ratios while also exhibiting a low number of unique CRLs, indicating that *target alteration* CRLs are conserved and often not decontextualized. On the other hand, *antibiotic target replacement* (*target replacement*) AROs are more mobilized by IS elements and plasmids (Fig. 3).

**Fig. 4:**
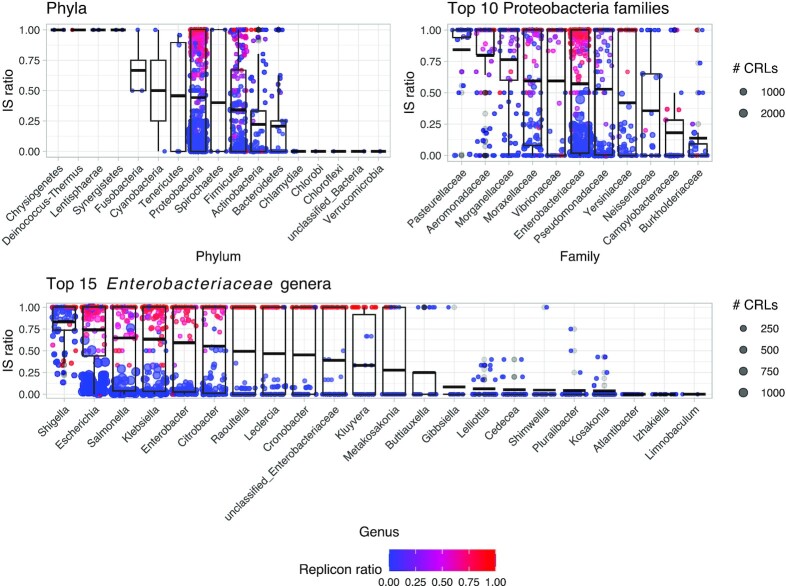
Taxonomic distribution of ARO categories. Boxplots and dots show IS ratio per taxonomic group. The size of the points indicates the number of unique CRLs in a given ARO, while the color is the replicon ratio, with highest (red) indicating more plasmid than chromosome placement of CRLs. Focus is placed on the Proteobacteria for plotting of families and genera. Boxes indicate first and third quartiles (25% and 75% of data) and horizontal lines in boxes show the median. Whiskers extend to 1.5* of the interquartile ranges.

ARGs are more decontextualized in clinically relevant genera (Fig. 4, Supplementary Figs. S12 and S13). As expected from database biases (Supplementary Figs. S5–S7), Proteobacteria harbor 88.18% of unclustered ARG loci (Supplementary Fig. S2). Although likely to be an artifact of selective sampling, Proteobacteria have been proposed to be the confirmed origin taxa of many acquired ARGs, although only an estimated 4% of ARGs have known bacterial origins [2]. Proteobacteria have a higher median IS ratio than Actinobacteria and Bacteriodetes (MWU test; *P*_adj_ < 0.01). Within Proteobacteria, *Enterobacteriaceae* have a higher median IS ratio than *Campylobacteriaceae*, and *Burkholderiaceae* but lower than *Aeromonadaceae, Pasterurellaceae*, and *Morganellaceae* (Fig. 4). However, CRLs in *Enterobacteriaceae* are more often found on plasmids than for *Campylobacteraceae, Moraxellaceae, Morganellaceae, Neisseriaceae, Pasteurellaceae, Pseudomonadaceae*, and *Burkholderiaceae* (Supplementary Fig. S12; MWU test; *P*_adj_ < 0.01), showing that many ARGs in *Enterobacteriaceae* are highly mobilized by both IS elements and plasmids. Within *Enterobacteriaceae*, many ARG loci have been mobilized both by IS elements and plasmids, especially within the genera *Shigella, Escherichia, Salmonella, Klebsiella, Enterobacter*, and, to a lesser degree, *Citrobacter* (Fig. 4). Other genera in *Enterobacteriaceae* show lower median mobilization degrees (significance values in Supplementary Table S3). *Enterobacteriaceae* genera with highly mobilized ARGs all have members of significant importance to human health and persistent fixation of mobilized ARGs is likely a consequence of human interference with pathogenic bacteria [54].

There are significant differences between the median IS ratios of phyla with, for example, Actinobacteria and Bacteriodetes having lower median IS ratios than Proteobacteria (MWU test; *P*_adj_ < 0.01). Within Firmicutes, whose IS ratio is not different from that of Proteobacteria, some families are associated with human activities such as *Enterococcaceae* and *Staphylococcaceae*. These harbor highly mobilized ARGs, while environmentally associated Firmicutes, such as *Bacillaceae*, have many ARGs barely mobilized by either IS elements or plasmids (Supplementary Fig. S13). This exemplifies how homologs of ARGs can be found in both environmental and clinically relevant genera, but that they have been decontextualized more in the latter. It should be noted that the *Bacillaceae* family has many members associated with humans, including the human gut. However, the “isolation source” and “host” modifiers for the downloaded RefSeq genome entries are severely lacking, making it impossible to meaningfully discern environmental from human-associated strains. As an example, only 16.5% of genome entries have “host” information with just 2.5% of that being *Homo sapiens* and only 30.9% have “isolation source” information, with the largest source being “soil” at 13.2%. This supports our assumption that a majority of *Bacillaceae* genomes are from environmental strains.

Generally, diving into specific families and genera shows that ARGs in human-associated bacteria are more mobilized than in others (Supplementary Table S3) and fixation of mobilized ARGs is likely a consequence of human interference with pathogenic bacteria [54]. This selection stemming mainly from antibiotic usage is obviously hugely important in the fixation of ARGs in the context of MGEs.

### Integron-association varies across ARG classes

Under selective pressure for resistance, ARGs may be decontextualized into integrons, where a strong promoter confers overexpression of said ARGs, which may result in phenotypic resistance [19]. Using IntegronFinder [63] on CRL sequences, 3,723 ARGs were identified as gene cassettes in integrons or clusters of *attC* sites lacking integron*–*integrases (CALINs). The most abundant major mechanism was *antibiotic inactivation* with 2,684 unique CRL occurrences. Mechanisms *antibiotic target replacement* and *antibiotic efflux* were found in association with integrons in 694 and 310 CRLs, respectively (Supplementary Fig. S14). At first glance, the sulfonamide resistance genes associated with Tn*402* class 1 integrons [19], *sul1–4*, were not the most frequent submechanism associated with integrons and here found associated with integrons in only 123 unique CRLs out of 2,017 total *sul1–4* CRLs. However, the *sul1* gene associated with class 1 integrons is found as a conserved segment in the 3′ part of the integron and does not have its own *attC* site and is therefore often missed by IntegronFinder. In fact, complete integrons and/or CALINs were found in proximity of *sul1* genes (ARO: 3000410) in 1,393 out of 1,527 unclustered regions, supporting the well-described association between class 1 integrons and *sul1* [19]. Trimethoprim resistance *dfr* genes associated with class 2 integrons and Tn*7* transposon [19] were here found in high abundance in association with integrons. The most abundant submechanism was the “*inactivation” ant(3′′)* category, whose genes encode aminoglycoside nucleotidylylating enzymes, with 1,009 CRLs associated with integrons. The *ant* genes are often found in association with integrons [64]. Here, the 5 *ant* AROs, *aadA, aadA2, aadA5, ant(3'')-IIa*, and *ant(2'')-Ia*, all display integron associations in at least 54.24% of their total CRL occurrences (Supplementary Table S4). Similarly, the aminoglycoside acetyltransferase-encoding *aac(6′)* AROs (*aac(6′)-Ib-cr, aac(6′′)-Ib10, aac(6′′)-Ib7*, and *aac(6′)-Ib9*) are here mostly found in integrons, agreeing with previous description of this class of ARGs [64]. Genes encoding OXA-9 and OXA-1 β-lactamases are found in integrons in 98.51% and 63.90% of the 67 and 277 CRLs, respectively, emphasizing that these ARGs are of concern (Supplementary Table S4).

From these results, it is evident that some target ARGs are highly associated with integrons and are thus more relevant to screen for in an environment with, for example, PCR or sequencing, if the aim is to predict phenotypic resistance. This again emphasizes the importance of considering the genetic contexts of ARGs.

### Mobilization assessment based on four parameters

**Fig. 5:**
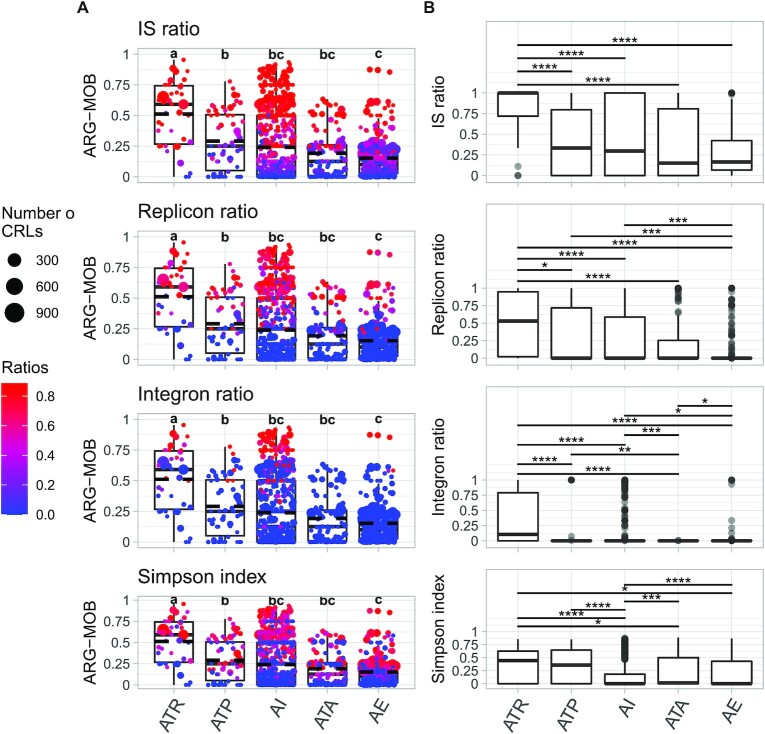
ARG-MOB scores of major resistance mechanisms defined from the 4 MOB parameters. (A) ARG-MOB score of AROs by major mechanism. Each point indicates a specific ARO and the size of the point corresponds to the number of unique CRLs in that ARO. Each of the four plots shows one of the individual MOB parameters as colored gradients of the points. Points are horizontally jittered but placed identically between the four plots in the left column. Mean is shown with dashed lines. Boxes indicate first and third quartiles (25% and 75% of data) and horizontal lines in boxes show the median. Whiskers extend to 1.5* of the interquartile ranges. Letters above boxplots indicate significant differences between mechanism populations (Mann–Whitney *U*-test with Holm–Bonferroni correction; *P* < 0.05). (B) Boxplots of each mobilization factor per major mechanism. Outliers are shown as gray dots. Above boxplots, bars indicate significant differences in distribution between mechanisms (Mann–Whitney *U*-test with Holm–Bonferroni correction). Only significant differences are displayed (*P < 0.05;**P < 0.01; ***P < 0.001; ^****^P ≤ 0.0001).

Inspired by previous work [23], we calculated a mobilization scale for each ARO, termed the ARG-MOB scale, which is calculated as the mean of the four mobilization parameters (MOB) and ranges from 0 to 1 with 1 representing very high mobilization, signified by very high IS and plasmid ratios, frequent association with integrons, and a wide phylogenetic dispersal across genera. Figure 5A shows the MOB parameters and ARG-MOB scale for each ARO. For each MOB parameter, boxplots with MWU test results are also shown (Fig. 5B).

The median ARG-MOB per major mechanism is highest for *antibiotic target replacement* (*P*_adj_ < 0.0001), while *antibiotic efflux* has a low median ARG-MOB but not significantly different from *antibiotic target alteration* and *antibiotic inactivation* groups. *Antibiotic target protection, antibiotic inactivation*,and *antibiotic target alteration* groups are not significantly different (Fig. 5A).

### Efflux genes are rarely mobilized

The *efflux* mechanism has the lowest median ARG-MOB (although only significantly lower than *target replacement* and *target protection*), which is reflected by median replicon, IS, and integron ratios that are lower than most other groups (Fig. 5B), that is, efflux genes are rarely mobilized by these MGEs. It is therefore likely that most identified efflux ARGs are part of core bacterial genomes located in conserved loci of chromosomes, with a few highly mobilized exceptions (Fig. 5A). This supports previous conclusions on efflux pump genes [7, 10, 39, 40]. While transient changes in expression or overexpression through mutations in transcriptional regulators may confer phenotypic resistance through extrusion of antibiotics, we advocate that genetic context needs to be considered when screening environments for efflux-associated ARGs. In clinical settings, the transient or constitutive expression of efflux pumps still warrants emphasis on screening for efflux-associated resistance markers.

The highest median ARG-MOB mechanism, *antibiotic target replacement*, is characterized by AROs with a high degree of mobilization by IS elements and plasmids (Fig. 5). The high ARG-MOB *target replacement* AROs are furthermore strongly associated with integrons and are taxonomically more widespread than *target alteration, inactivation*, and *efflux* groups (Fig. 5B). Likewise, some *target protection* AROs are highly mobilized and widespread but they are to a lesser degree associated with integrons. Generally, *target replacement* is significantly more associated with integrons than other categories, although the median of *inactivation* is higher than *target alteration* and *efflux*.

AROs of *inactivation* mechanisms are the least phylogenetically dispersed but are instead conserved within few genera, as indicated by the lowest median Simpson index. Possibly, many genes and/or proteins under the *inactivation* mechanism only function in specific genera, whereas those of other mechanisms can function in wider ranges of genera. While there are *inactivation* AROs that have been mobilized by plasmids, transposons, and integrons, there are many others that have not been decontextualized (Fig. 5A). All major mechanisms have exceptions in the form of AROs with elevated ARG-MOB, as evaluated on all four parameters, although *target alteration, target protection*, and *efflux* have few or no AROs with ARG-MOB higher than 0.75.

### The ARG-MOB scale proficiently describes decontextualization of ARGs

**Fig. 6:**
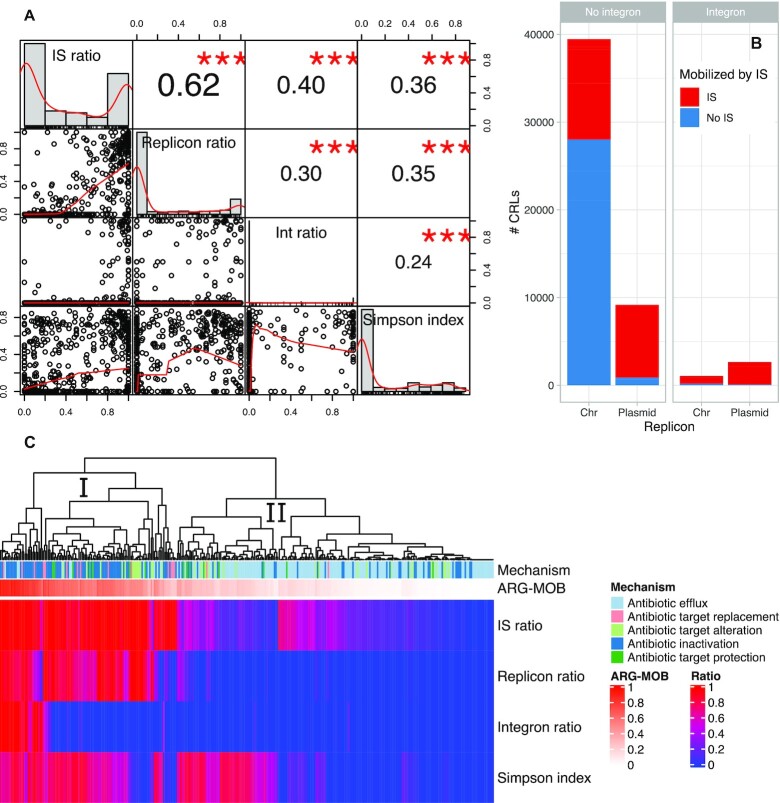
Correlation and co-occurrence of MOB parameters. (A) Pearson correlation coefficients between MOB parameters. Scatterplots between pairwise MOB parameters are shown in the lower left corner. The diagonal shows histograms of distribution of each MOB parameter. The values in the upper right corner show the Pearson correlation coefficients with significance levels (***P < 0.001). (B) Barplot of mobilization of unique CRLs by IS elements, plasmids, and integrons. (C) Heatmap of highly abundant AROs with at least 20 CRLs. The dendrogram shows clustering of the AROs, based on the four MOB parameters, and was calculated using standard parameters in the “ComplexHeatmap” package (complete hierarchical clustering on Euclidean distances).

The four MOB parameters all correlate significantly with each other, showing that they covary and are appropriate for calculating the ARG-MOB scale (Fig. 6A,B). Hierarchical clustering of AROs from all five major mechanisms in a heatmap shows apparent mechanism-specific profiles of ARG-MOB scores, as well as each of the four MOB parameters (Fig. 6C). Two major branches are formed from clustering: (I) a high-ARG-MOB branch dominated by *inactivation*, as well as other individual AROs from other mechanisms, and (II) a low-ARG-MOB branch mostly populated by *efflux* AROs.

The highest correlation coefficient is seen for IS/replicon ratios, showing that ARGs placed on plasmids are likely mobilized by IS elements prior to insertion on plasmids (Fig. 6A,B). The second highest correlation is found between IS and integron ratios, indicating that ARGs, found as gene cassettes in integrons, are likely to have been mobilized (as part of integrons) by IS elements (Fig. 6A,B), which has been often reported and discussed [18, 19, 55]. To a lesser degree, ARGs found on plasmids are correlated with integrons.

Not surprisingly, the Simpson diversity index correlates positively with IS, replicon, and integron ratios (Fig. 6A), showing that highly mobilized ARGs are also likely to be phylogenetically widespread. On the basis of these correlations, we conclude that the ARG-MOB ratio proficiently describes decontextualization of ARGs. Pearson correlation coefficients and MGE co-occurrences were also calculated per mechanism (Supplementary Text 7, Supplementary Figs. S15–S20).

### Some AROs are highly divergent in mobilization

Many AROs can be defined as either highly mobilized or only to a very small degree. Still, some AROs have a large spread from their mean ARG-MOB score, showing that they are most often sitting unmobilized on a chromosome, but have one or more times been mobilized and widely dispersed (Supplementary Figs. S21 and S22). This is exemplified by the efflux pump genes *oqxAB* (ARO3003922-3) [65, 66] (Supplementary Text 8). These genes are found on essentially all *Klebsiella pneumoniae* chromosomes, where they do not confer resistance unless overexpressed [67–69], as seen when placed close to IS elements on plasmid pOLA52 [65]. The *oqx* AROs show high spread across their mean IS and replicon ratios (*oqxA* has IS and replicon ratios of 0.35 and 0.16, respectively), showing that their mean ratios are low due to *Klebsiella* chromosomes but that there are many outliers due to variants in *Escherichia* and *Salmonella* that are only found mobilized by IS elements and usually on plasmids (Supplementary Fig. S21, Supplementary Table S5).

Outliers from the mean of IS and replicon ratios can also be considered per genus instead of ARO, in order to highlight that ARGs in some genera are much more mobilized than in others. For example, efflux pump genes in *Shigella* are more associated with IS elements compared to the global average, but they are not found on plasmids more than on average (Supplementary Fig. S22). Likewise, many *antibiotic inactivation* ARGs are found more on plasmids in *Escherichia, Salmonella, Klebsiella, Citrobacter*, and *Enterobacter* than their respective average placements per ARO. Other genera including *Proteus, Pseudomonas, Acinetobacter*, and *Morganella* tend to have some *inactivation* ARGs more located on chromosomes than the given ARO average, indicating that chromosomes in these genera may be considered reservoirs of potential genes with potential as resistance determinants. This highlights the complexity of the ARG issue and emphasizes the importance of considering the genetic context before predicting resistance.

### Defining ARG-MOB categories

**Fig. 7:**
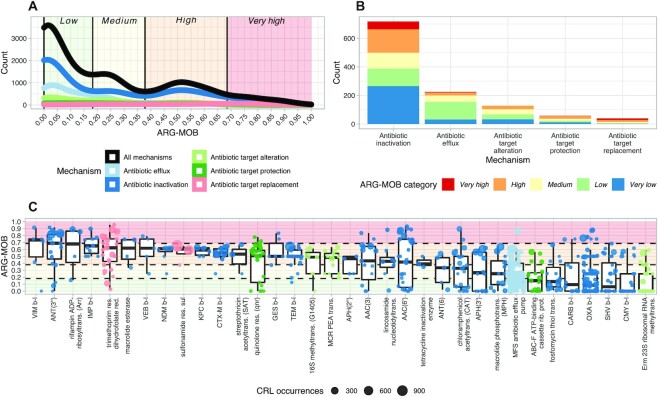
Density of ARG-MOB categories and distribution per major resistance mechanism. (A) Count density of ARG-MOB per mechanism. Only major nonhybrid mechanisms are shown. (B) Count of each ARG-MOB category per mechanism. (C) All submechanisms that have AROs with *High* or *Very high* ARG-MOB scores. Points are colored by major resistance mechanism as in panel (A) and sized according to the number of CRLs of the given ARO. The background is colored similarly to panel (A), representing *Low, Medium, High*, and *Very high* categories. The Very low category is for ARG-MOB = 0 and does thus not have a background color in the graph.

Smoothed kernel density estimates of AROs and their ARG-MOB values are shown in Fig. 7A per mechanism and cumulatively for all mechanisms. The following five ARG-MOB groupings were defined computationally: *Very low* (ARG-MOB = 0), *Low* (0 < ARG-MOB < 0.182), *Medium* (0.182 < ARG-MOB < 0.378), *High* (0.378 < ARG-MOB < 0.681), and *Very high* (ARG-MOB > 0.681). These definitions are largely the same when estimating per mechanism individually (Supplementary Fig. S23). Numerically, *inactivation* has the highest number of *High* and *Very high* ARG-MOB AROs (144 and 54, respectively), while *target replacement* has the highest percentage of *High* and *Very high* ARG-MOB AROs with these categories representing 64% of *target replacement* AROs (Fig. 7B).

### *High* ARG-MOB AROs correspond with high-risk ARGs

*High* and *Very high* ARG-MOB AROs (Fig. 7C) are mainly ARGs that were initially identified in resistant pathogens where they indeed confer resistance. Conversely, many low ARG-MOB AROs have only been shown to confer resistance when placed on high-expression cloning vectors but not in any natural wild-type isolate. A few examples are described below and in Supplementary Text 9. A table for all 1,176 AROs can be found as an interactive table in Additional File 1.

For all major mechanisms, many AROs are classified as *Very low* or *Low* ARG-MOB (Fig. 7C) and *antibiotic target alteration* does not have any *Very high* ARG-MOB AROs, while *target protection* has two (ARO3002803 and ARO3002801; quinolone resistance genes *qnrVC6* and *qnrVC4*). *Inactivation* has many AROs with *High* and *Very high* ARG-MOB, which include infamous β-lactamases, aminoglycoside nucleotidyltransferases (ANTs), and others (Fig. 7C). Aminoglycoside resistance by *antibiotic inactivation* is highly represented by ARGs scoring *High* and *Very high* on the ARG-MOB scale, showing that aminoglycoside resistance is in many cases highly mobilized (Supplementary Fig. S24). Likewise, ARGs encoding resistance to up to five groups of β-lactam antibiotics (carbapenem, cephalosporin, cephamycin, penam, and penem) are highly mobilized, highlighting the critical state of resistance toward these antibiotics (Supplementary Fig. S24). With a median ARG-MOB of 0.73, the gene encoding the Verone integron-encoded metallo-β-lactamase (VIM) is the β-lactamase gene with the highest median ARG-MOB. There are three VIM β-lactamase AROs, of which ARO3002271 has the highest ARG-MOB of any *inactivation* ARO at 0.91. In RefSeq complete genomes, the gene is only found inserted in integrons and is located close to IS elements and on plasmids in 95% of the CRLs found (*n* = 21). It is dispersed across 6 unique genera for a Simpson index of 0.75 (*Pseudomonas, Salmonella, Escherichia, Klebsiella, Citrobacter*, and *Enterobacter*). The VIM-1 gene was found in a multiresistant *E. coli* from a patient. It was inserted in a class 1 integron and found on a conjugative plasmid [70]. It has since been seen in multiple *Enterobacteriaceae*, typically in association with MGEs, and is globally spread [71].

The highest ARG-MOB *target replacement* AROs belong to the trimethoprim-resistant dihydrofolate reductase *dfr* submechanism. The ARO3003013 within this submechanism has the highest ARG-MOB of any ARO at 0.95. A class 1 integron with *dfrA15* is widespread in *Vibrio cholera* isolates in Africa and was found on a conjugative plasmid [72]. It is the ARO with the highest ARG-MOB, since it was only found to be associated with IS elements, integrons, and plasmids (all ratios = 1). It has a Simpson index of 0.82 and the 7 CRLs are dispersed across 6 genera (*Vibrio, Salmonella, Enterobacter, Leclercia, Klebsiella*, and *Escherichia*). Generally, ARGs conferring diaminopyrimidine (including trimethoprim) resistance by *antibiotic target replacement* mechanism are highly mobilized and numerous in the complete genomes studied here (Supplementary Fig. S24).

## Discussion

The ARG–MGE association aspect has received much attention recently [2, 3, 18]. For instance, it was shown that mobilized ARGs often have confirmed origins in Proteobacteria, especially from human- and animal-associated species, although the confirmed origins have only been found for an estimated 4% of ARGs [2]. This is likely due to selective pressure for resistance in these environments that make them ARG mobilization hotspots [2]. This is supported in our work where 88.18% of ARG loci are found in Proteobacteria but is also elaborated by our finding that Proteobacteria not only is the origin of many ARGs but also harbors the bulk of mobilized ARGs, especially within the *Enterobacteriaceae*. Of course, database biases (Supplementary Text 2) strongly influence the findings presented here and elsewhere [2].

The publications described above were part of our inspiration for the work in this study, which expands on the subject of ARG–MGE associations. We have applied a similar context-focused approach as these [2, 3], although we here, with empirical evidence, investigate a distance of 12.17 kbp from ARGs rather than the distances of 10 kbp or 10 open reading frames in the other studies. Furthermore, the threshold for including ARG matches is more stringent in our study at 80% similarity and 80% query coverage, compared to the other studies with 90%/50% [3] and 70%/80% [2], respectively. The two referenced papers are from the same research group and are published less than 6 months apart, highlighting that there is no consensus strategy for finding ARG homologs, although a standardized approach is sorely needed. The same research group published in 2019 the tool fARGene [73], which applies curated hidden Markov models to make ARG predictions. While the sensitivity is surely higher with such model-based approaches, they are severely limited by the number of available models, which, at the time of writing, are not sufficient for large-scale studies such as that presented here.

It is well established that mobile ARGs pose a more concrete threat [23], although it has been argued that all ARGs, irrespective of whether they have ever been mobilized or found in a clinical isolate, should be considered a potential threat [28]. With the astounding sizes of current ARG databases in the thousands of genes, surely there must be ARGs that pose a bigger threat than others? The ARG-MOB score presented here is our approach for identifying target ARGs that have been mobilized in bacterial genomes.

Our results clearly demonstrate the importance of including the genetic context in ARG predictions, since even the highest ARG-MOB scoring genes have representatives that are not decontextualized and may not confer resistance. This study documents how genes from even the most mobilized categories of ARGs can be found unmobilized on chromosomes. Therefore, the validity of using PCR-based screening to assess the abundance and distribution of putative ARGs is questionable at best, unless context is likewise included in PCR design, as seen before [74] and suggested elsewhere [3]. Including this aspect in future studies may help to alleviate the occasional discrepancies between genotypic and phenotypic resistance predictions [8, 29–32], with especially efflux-related markers producing a high number of false-positive predictions [34]. Even the presence of β-lactamase genes cannot solely be used as predictors of resistance, as they are involved in regular cell upkeep [25] and are in this study found to not have been mobilized in 80% of their genomic occurrences (see Additional File 1). It should be noted that other studies did find high congruence between genotypic and phenotypic resistance [35–38], using well-known human pathogens and subsets of curated ARG databases. This highlights that using very large ARG databases to screen nonclinical environments for resistance markers is likely to result in false-positive resistance predictions.

Based on examples of high and low ARG-MOB AROs (Supplementary Text 9), a pattern emerges that high ARG-MOB AROs, such as the *bla(VIM)* [70], *dfrA15* [72], *aac(6′)* [75], and *arr-2* [76], were originally identified in already virulent, pathogenic bacteria that had indeed been verified to be resistant. On the other hand, low ARG-MOB AROs were generally identified in susceptible bacteria and/or only shown to cause resistance when cloned into vectors with strong gene expression, such as *murA* [45], *norB* [46], and *bla(CME-1)* [48] (Supplementary Text 9). This warrants caution when choosing ARGs of interest in either targeted quantitative PCR screening or metagenomic sequencing of environmental samples. We advocate that knowledge of ARG mobilization paired with other factors, such as trends in antibiotic usage [77], will allow us to better understand ARGs of concern and to predict future problematic resistance determinants. A worrying aspect in our results is the extent to which several classes of genes encoding broad-spectrum β-lactamases are found to be highly mobilized (Fig. 7), coupled with the fact that penicillins and other β-lactams have seen a great increase in global usage in recent years [77].

## Potential Implications

Based on the results presented here and as discussed elsewhere [11, 23, 57], it is clearly necessary to consider the genetic context of genes when predicting ARGs from (meta)genomes. This could be achieved by applying PCR primers that target regions spanning both an ARG and an associated MGE [74]. For more accurate ARG calling, metagenomic sequencing using long-read platforms is a prerequisite to enable the detection of ARGs and their genetic contexts. For more targeted investigations where a “meta” approach is either not feasible or within scope, we provide a comprehensive and interactive table of the results presented in this study (see Additional File 1). This table can be used as a tool to select more relevant possible resistant determinants in future studies. We are strong proponents of more focused and accurate predictions of true ARGs, especially when dealing with environmental samples, as it is vital that the serious resistance issue is managed and discussed with diligence and precision.

## Methods

### Databases and ARG prediction

All code for data processing was written in BASH scripts, and statistics and plotting were primarily done in RStudio.

All complete bacterial genomes (15,790 entries with 16,785 chromosomes and 14,280 plasmids) were downloaded from RefSeq on 12 December 2019 using the ncbi-genome-download tool v0.2.11 [78]. In order to ensure uniform prediction of genes across all bacterial genomes, Prodigal [79] (v2.6.3; RRID:SCR_011936) was used to predict genes from nucleotide sequences and write corresponding amino acid sequences from all RefSeq genomes. Since Prodigal first trains itself based on the input sequence, gene prediction was performed on subsets of each genus present in RefSeq genomes. Per genus, two rounds of Prodigal were performed with the -meta flag enabled in the second run, as it predicts some genes that are missed in single genome mode and vice versa. Results from the “single” and “meta” gene predictions were consolidated to omit redundancy.

Several ARG databases and tools for predicting ARGs have been produced, including CARD [80], ARDB [81], MEGARes [82], ResFinder [83], SARG [84], ARG-ANNOT [85], DeepARG-DB [86], ARGminer [87], FARME [88], and others. Some are discontinued while others receive updates occasionally. The CARD database is large, actively updated, well curated, and widely used. Furthermore, it makes use of ontology terms (Antibiotic Resistance Ontology: ARO) that allow for the grouping of resistance genes according to resistance mechanisms. Because of these advantages over other databases and the essential role of ontology terms, the CARD database was used in this study. The “protein homolog” models from CARD were used here, since they do not contain resistance determinants that are based on mutations. The main resistance mechanisms defined in the CARD database are “*antibiotic efflux*” (*efflux*), “*antibiotic inactivation*” (*inactivation*), “*antibiotic target alteration*” (*target alteration*), “*antibiotic target protection*” (*target protection*), “*antibiotic target replacement*” (*target replacement*), and the less abundant “*reduced permeability to antibiotic*” (*reduced permeability*). A few additional categories exist that are hybrids of two of the above mechanisms, but there are very few entries of these in CARD and are for most of the analyses not considered.

The CARD database (v3.0.7) was downloaded, and only the protein homolog model was used in this study, excluding resistance determinants related to sequence variants (e.g., single-nucleotide polymorphisms). DIAMOND [89] blastp (BLASTP,0020RRID:SCR_001010) was used to identify ARGs in all RefSeq genomes. For blastp against the CARD database, both query and subject coverages were set to a minimum of 80%, while E-value cutoffs were set to 1e-10, to limit the rate of spurious hits. For each query protein from all RefSeq genomes, only the single best CARD match was kept.

The CARD auxiliary tool, RGI [90], for predicting ARGs in (meta)genomes, uses curated blastp bitscore cutoffs unique to every ARG protein in the CARD database. The same bitscore cutoffs were applied here, with the exception that hits with bitscores lower than the RGI cutoff were included if they had an identity score and a query coverage of at least 80%. These hits were included in order to keep more ARG hits from environmental bacteria that are not clinically relevant, since it is assumed that CARD and other ARG databases are biased toward genes that reside in anthropogenically relevant strains. Blastp hits with bitscores above the RGI cutoff were also only kept if query coverage was at least 80%. The effects of these filters are further described in Supporting Information (Supplementary Text 1, Supplementary Figs. S1–S3).

### Extracting the genetic context of ARGs

The average length of composite and unit transposons was calculated based on 449 entries in The Transposon Registry [62]. This average (12.17 kbp) was used as the maximum allowed distance between an ARG and an IS element for classifying an association (Supplementary Table S1). However, since ARGs in transposons can be on either strand relative to the transposase, IS elements are identified within 12.17 kbp of an ARG in both directions. This enables searching for transposons of up to 24.34 kbp (plus the length of the identified ARG), which would include 77.73% of the 449 composite and unit transposons in The Transposon Registry [62] (Supplementary Fig. S4).

For all filtered blastp ARO hits, up to 12,170 bp both up- and downstream of the hit were extracted from the respective RefSeq replicon using the faidx command from Samtools [91, 92] (v1.9–166-g74718c2; RRID:SCR_002105). If an ARG was found within 12.17 kbp of either terminus of a replicon, only sequence until the terminus was extracted and not continued from the other end of the sequence, since entries in RefSeq complete genomes may not be actually complete, due to low sequencing coverage regions stemming from, for example, genomic GC-content biases in sequencing [93]. Loci were categorized according to the ARO of the identified ARG. There are 9 ARO major mechanism categories, of which three are less abundant “hybrids” merged by two other categories. The 6 nonhybrid categories *efflux, inactivation, target alteration, target replacement, target protection*, and *reduced permeability* are here considered the main categories and are the ones mainly investigated in this study. The mechanism *reduced permeability* is only represented by three ARO categories and is excluded from some statistical analyses.

### IS elements in ARG loci and 16S rRNA as control

IS elements in ARG loci were predicted using DIAMOND blastp against the ISfinder database [94], as implemented in Prokka [95] (v1.14.0; RRID:SCR_014732). The same E-value cutoff for IS annotations, as Prokka applies during gene annotation (1e-30), was used here and the minimum query coverage accepted was 90%. Only the top IS hit for each query protein was kept, since multiple “good” hits to distinct IS families may occur per query. The distance between a given ARG and its closest IS neighbor within 12.17 kbp in either direction (if any) was calculated without considering the coding strand of the genes. ARGs not within 12.17 kbp of an IS element were not considered when calculating the mean ARG–IS element distances.

Since 16S rRNA genes are not expected to be often mobilized by IS elements, the distance between 16S rRNA genes and IS elements was explored in all complete RefSeq bacterial genomes, in order to assess how many “false-positive” ARG–IS associations are expected to be identified using the 12.17-kbp distance cutoff (Supplementary Text 9). In total, 80,141 16S rRNA genes in 15,790 strains were predicted using barrnap [96]. Of these, 94.61% did not have identified IS elements within 12,170 bp in either direction, which can be seen as analogous to a 95% confidence interval for predicting an association between ARGs and IS elements.

### Clustering ARG loci to remove redundancy

Extracted loci with ARGs were grouped based on the CARD ARO category of the loci ARGs. In order to remove redundancy from the RefSeq database, stemming from overrepresentation of, for example, almost identical *E. coli* chromosomes, extracted loci were clustered with USEARCH [97] (v11.0.667_i86linux64) (Supplementary Texts 2–3, Supplementary Figs. S5 and S6). Per ARO group, sequence loci were clustered into what we refer to here as CRLs using the “-cluster_fast” command with the criteria that sequences in a cluster are at least 99% similar over at least 90% of the length (both target and query coverage) and only the single best hit was allowed per sequence. The “-sort length” flag was also enabled to sort loci by length before clustering, since loci vary in length (sum of 12.17 kbp up- and downstream plus an ARG of varying length). This ensures that loci of identical length (with the exact same ARG) are merged into the same CRLs. For each CRL, the centroid sequence was used as a representative sequence for downstream analyses.

### Integron prediction

Integrons and cassette arrays were predicted using IntegronFinder [63] using the centroid CRL sequences as input. IntegronFinder can predict complete integrons including gene cassettes, In0 elements where only integrase is present, and CALINs. All three classes of integrons are included in the analyses and no distinction is made, since an ARG observed in, for example, a CALIN has been previously associated with an integron and may still be in related, but not sequenced, strains.

### Statistical analysis

Data tables were imported into R for statistics and visualization using the packages ggplot2 (ggplot2, RRID:SCR_014601), dplyr (dplyr, RRID:SCR_016708), tidyr (tidyr, RRID:SCR_017102), gridExtra, ggpubr (ggpubr, RRID:SCR_021139), ggExtra, reshape2, knitr (knitr, RRID:SCR_018533), kableExtra, vegan (vegan, RRID:SCR_011950), PerformanceAnalytics, ComplexHeatmap (ComplexHeatmap, RRID:SCR_017270), RColorBrewer (RColorBrewer, RRID:SCR_016697), DT, rstatix (rstatix, RRID:SCR_021240), tidyverse (tidyverse, RRID:SCR_019186), broom, and plotly (Plotly, RRID:SCR_013991). Prior to pairwise *post hoc* tests, all data sets were tested for whether samples originate from the same distribution using nonparametric Kruskal–Wallis tests. Significance was observed for all data sets, allowing for pairwise *post hoc* tests. Subsequently, statistical tests on rank sums of groupings were performed with unpaired MWU with Holm–Bonferroni correction for multiple testing, since this method does not require independence. All reported *P*-values are Holm–Bonferroni corrected. Pairwise correlation analyses between MOB parameters were calculated with Pearson correlation coefficients and significance tested with the R function cor.test. A clustered dendrogram of clustering of the AROs, based on the four MOB parameters, was calculated using standard parameters in the “ComplexHeatmap” package (complete hierarchical clustering on Euclidean distances).

### MOB metrics and the ARG-MOB scale

Four main mobilization (MOB) metrics (Fig. 2), or ratios (0 to 1), of mobilization were calculated per ARO that aim to quantify just how mobilized groups of ARGs are. These four ratios are (i) the replicon ratio, (ii) the IS ratio, (iii) the integron ratio, and (iv) the phylogenetic spread of an ARO across distinct genera, quantified by the Simpson diversity index. Pearson correlation coefficients between MOB metrics were calculated.

For each ARO category, the number of CRLs with and without identified IS elements was counted and the IS ratio was derived where an IS ratio of 1 indicates that all CRLs belonging to a given ARO have an IS element within 12,170 bp either up- or downstream of the ARG. Vice versa, an IS ratio of 0 indicates that none of the CRLs in an ARO have IS elements in proximity. Similarly, the replicon ratio was calculated per ARO based on the CRLs’ location on either plasmids or chromosomes. A replicon ratio of 1 means that all CRLs in a given ARO are of plasmid origin and a 0 means that all CRLs are from chromosomes. The integron ratio indicates how many CRLs are inserted in integrons per ARO. For measuring the taxonomic distribution of each ARO category, the Simpson diversity index (range 0 to 1) was calculated per ARO using unclustered sequences and the genera they were identified in.

The ARG-MOB scale (0–1) represents the mean of the four MOB metrics described above (plasmid, IS, and integron association and Simpson diversity index) and serves as a ranking scheme to evaluate the degree to which members of an AROs have been mobilized. Based on the smoothed kernel density estimates of all ARG-MOB scores, groupings were made to categorize AROs by their ARG-MOB score. An ARG-MOB score of 0 indicates that ARGs of the given ARO were not once found to be mobilized in the RefSeq genomes and a score of 0 is thus categorized as *Very low*. Valleys in the density distribution of ARG-MOB scores were used to computationally pinpoint thresholds between ARG-MOB categories. The ARG-MOB score in the *Low* group ranges from 0.0 to 0.182, the *Medium* group ranges from 0.182 to 0.378, *High* ranges from 0.378 to 0.685, and *Very high* ranges from 0.685 to 1.0. For the *Low-Medium* and *Medium-High* cutoffs, the low point in valleys was used to define values but no apparent valley is present between *High* and *Very high*. Instead, a linear model was fitted to the right-side slope of the *High* peak and another fitted to the approximately linear data range starting at ARG-MOB score of 0.7. The intersection between the two linear models (0.685) was used as the cutoff between the *High* and *Very high* groups (Supplementary Fig. S8).

## Additional Files

**Additional File 1**. Dataset1.html. An interactive table summarizing the results presented in the study. This file can be opened in a web browser.

The file “ARGMOB_Supplementary Material.pdf” contains the following supplementary materials:

**Supplementary Text 1**. Additional details about filtering of DIAMOND blastp hits against CARD database.

**Supplementary Fig. 1**. Distribution of DIAMOND blastp hits plotted as % ID against the bitratio. The defined filters are shown.

**Supplementary Fig. 2**. Major bacterial orders of ARG blastp hits passing either of the defined filters. This plot shows the taxonomic distribution of passing hits on the Order level. Only orders that constitute >0.2% of the total hits are shown here.

**Supplementary Fig. 3**. Minor bacterial orders of ARG blastp hits passing either of the defined filters. This plot shows the taxonomic distribution of passing hits on the Order level. Only orders that constitute <0.2% of the total hits are shown here.

**Supplementary Fig. 4**. Violin plot of the lengths of unit- and composite transposons in The Transposon Registry. The mean length of transposons from this curated database is used to find the length cutoff for investigating the proximity of ARGs.

**Supplementary Table 1**. Summary of filters and cutoffs for including ARG and IS element blastp hits.

**Supplementary Text 2**. Investigation of the taxonomic biases and skews of the RefSeq complete genome database and the CARD database. These two databases are both biased, but they are not biased towards the same genera.

**Supplementary Fig. 5**. Biased composition of the RefSeq complete genome database investigated at Order level.

**Supplementary Fig. 6**. Comparison of the relative representation of genera in the RefSeq complete genome database and the CARD database. The databases are not skewed towards the same genera. The biggest differences are from the genera *Acinetobacter, Klebsiella, Escherichia, Salmonella, Streptococcus*, and *Bordetella*.

**Supplementary Text 3**. By clustering 176,688 genetic loci with ARGs to 53,895 Clustered Resistance Loci, we reduce the differential biases between the databases. Furthermore, we reduce the compositional database biases stemming from the presence of many almost identical genomes (e.g. *E. coli* genomes) in RefSeq.

**Supplementary Fig. 7**. Effect of clustering ARG loci to CRLs. Several of the most abundant genera in RefSeq are reduced in their relative abundance by clustering of almost identical ARG loci. Loci with antibiotic efflux ARGs are compressed more by clustering than other resistance mechanisms.

**Supplementary Table 2**. Overview of blastp hits passing filters, number of CRLs formed, and total number of AROs represented.

**Supplementary Fig. 8**. Smoothed kernel density estimates of all AROs and their ARG-MOB values. In this density plot, the cutoffs between ARG-MOB groupings (calculated by identification of local minima in density distribution) are shown. For the cutoff between High and Very high groups, no local minimum could be found. Instead, linear models were fitted in two approximately linear ARG-MOB ranges. The intersection of the two linear models were chosen as the cutoff.

**Supplementary Text 4 and Supplementary Figure 9**. 16S rRNA genes were identified in the studied complete genomes and IS elements were identified within the same proximity as for ARGs. This serves as a control, in that 16S rRNA is rarely expected to be associated with IS elements. Within 12.17 kbp on either side of 16S rRNA genes, IS elements were identified in 5.59% of cases. We consider this an indication of a false-positive rate of approx. 5% of association of ARGs with IS elements.

**Supplementary Text 5**. Investigation of which IS element families co-occur with individual resistance mechanisms.

**Supplementary Fig. 10**. Boxplots of abundance of IS families in proximity to ARGs, shown by major resistance mechanism. Significance values for Mann-Whitney tests are shown above boxplots.

**Supplementary Text 6**. The distance, in terms of bases, between IS elements and ARGs are discussed here. Efflux ARGs have significantly larger distance to IS elements than other mechanisms.

**Supplementary Fig. 11**. a: Density plots of IS ratio against distance to nearest IS element per ARO. b: boxplot of the ARG-IS distance per resistance mechanism with Mann-Whitney tests.

**Supplementary Fig. 12**. Replicon ratios of the 10 most abundant Proteobacteria families. ARO points are colored by their Replicon ratio and sized by how many unique CRLs belong to them.

**Supplementary Fig. 13**. IS ratios of Firmicutes families. ARO points are colored by their Replicon ratio and sized by how many unique CRLs belong to them. Many Firmicutes families are associated with the environment but some are associated with human activites, such as *Enterococcaceae* and *Staphylococcaceae*. This plot illustrates well the differences in ARG mobilization in environmental and human-associated bacteria.

**Supplementary Table 3**. Pairwise Mann-Whitney tests for IS and Replicon ratios in *Enterobacteriaceae* genera.

**Supplementary Fig. 14**. a: Overview of major mechanisms associated with integrons. b: submechanisms associated with integrons. ANT(3’’) is the most integron-associated submechanism.

**Supplementary Table 4**. Table of the top 20 AROs associated with integrons with their % integron association.

**Supplementary Text 7 and Supplementary Figures 15-19**. Pearson correlation coefficient analyses for major mechanisms. For each of the five major mechanisms, pairwise Pearson correlation coefficients are calculated and shown, similar to main figure 5a.

**Supplementary Fig. 20**. Bar charts of co-occurrence of IS elements, chromosome/plasmid location, and integrons per major mechanism. This shows that e.g. efflux ARGs are most often located on chromosomes and not in association with integrons or IS elements. On the other hand, inactivation ARGs are often found on plasmids and in association with IS elements and integrons.

**Supplementary Text 8, Supplementary Figures 21-22, and Supplementary Table 5**. Some AROs are highly divergent in mobilization. It is here discussed that some AROs have a large spread from their mean IS and Replicon ratios. As discussed in the main text, the oqxAB efflux pump genes are good examples of AROs with low IS and Replicon ratio means, since they are found un-mobilized on almost all *Klebsiella* genomes, but have been mobilized in many *Escherichia* and *Salmonella* strains, resulting in large spreads from the mean IS and Replicon ratios. It is also discussed that *Proteus, Pseudomonas, Morganella*, and *Acinetobacter* may act as reservoirs of yet unmobilized potential ARGs.

**Supplementary Fig. 23**. Count density of ARG-MOB per mechanism. Similar to main figure 6a, the count density of ARG-MOB categories are shown per major resistance mechanism.

**Supplementary Figure 24**. Antimicrobial compounds to which there are ARGs with Very high or High ARG-MOB scores.

**Supplementary Text 9**. Some examples Additional information and analyses for integron-ARG associations. Some examples of low and high ARG-MOB AROs are discussed with references. These examples illustrate that low ARG-MOB scoring AROs have generally only been shown to confer resistance in broad screenings where genes have been randomly cloned into strong expression vectors. On the other hand, high ARG-MOB scoring AROs were identified in clinical isolates that were experimentally verified to be resistant.

## Availability of Source Code and Requirements

Project name: ARG-MOB

Project home page: https://github.com/tueknielsen/ARG-MOB

Operating system: Linux—Ubuntu

Programming language: bash, R

Other requirements: For a full list of software and databases, please consult the script ARG_MOB_v0_5.sh at https://github.com/tueknielsen/ARG-MOB.

License: MIT license

## Data Availability

A searchable table of all results is available online as Dataset S1 (Additional File 1). All supporting data and materials are available in the *GigaScience* GigaDB database [98].

## Abbreviations

ANT: aminoglycoside nucleotidyltransferases; ARG: antibiotic resistance gene; ARO: antibiotic resistance ontology; bp: base pair; CALIN: cluster of *attC* sites lacking integron–integrases; CRL: clustered resistance locus; ICE: integrative conjugative element; IS: insertion sequence; MGE: mobile genetic element; MOB: mobilization parameter; MWU: Mann–Whitney *U*; PCR: polymerase chain reaction; rRNA: ribosomal RNA; VIM: Verone integron-encoded metallo-β-lactamase.

## Authors' Contributions

Conceptualization: TKN, LHH; Methodology: TKN, PDB, LHH; Investigation: TKN, PDB; Visualization: TKN; Supervision: LHH; Writing—original draft: TKN, PDB, LHH; Writing—review & editing: TKN, PDB, LHH.

## Competing Interests

The authors declare that they have no competing interests.

## Supplementary Material

giac072_GIGA-D-22-00081_Original_Submission

giac072_Reviewer_1_Report_Original_Submission Qing-Lin Chen -- 5/29/2022 Reviewed

giac072_Reviewer_2_Report_Original_Submission Timothy Ghaly -- 5/31/2022 Reviewed

giac072_Supplemental_Files
