# **Correction to:** Antibiotic resistance genes are differentially mobilized according to resistance mechanism

**DOI:** 10.1093/gigascience/giad012

**Published:** 2023-03-06

This is a correction to: Tue Kjærgaard Nielsen, Patrick Denis Browne, Lars Hestbjerg Hansen, Antibiotic resistance genes are differentially mobilized according to resistance mechanism, GigaScience, Volume 11, 2022, giac072, https://doi.org/10.1093/gigascience/giac072

In the originally published version of this manuscript, the legend scales in Figures 3 and 4 were incorrect.

This error has now been corrected.

