# Supplementary material for: Antibiotic resistance genes are differentially mobilized according to resistance mechanism
Source: Gigascience. 2022 Jul 30;11:giac072. doi: 10.1093/gigascience/giac072 (PMC9338424; doi:10.1093/gigascience/giac072)
Supplement: giac072_GIGA-D-22-00081_Original_Submission [file giac072_giga-d-22-00081_original_submission.pdf]

GigaScience

Antibiotic resistance genes are differentially mobilized according to resistance mechanism

--Manuscript Draft--

Manuscript Number:	GIGA-D-22-00081
Full Title:	Antibiotic resistance genes are differentially mobilized according to resistance mechanism
Article Type:	Research
Funding Information:	
Abstract:	Background<br><br>Screening for antibiotic resistance genes (ARGs) in, especially environmental, samples with (meta)genomic sequencing is associated with false-positive predictions of phenotypic resistance. This stems from the fact that most acquired ARGs require being overexpressed before conferring resistance, which is often caused by decontextualisation of putative ARGs by mobile genetic elements (MGEs). Consequent overexpression of ARGs can be caused by strong promoters often present in insertion sequence (IS) elements and integrons and the copy number effect of plasmids which may contribute to high expression of accessory genes.<br><br>Results<br><br>Here, we screen all complete bacterial RefSeq genomes for ARGs. The genetic contexts of detected ARGs are investigated for IS elements, integrons, plasmids, and phylogenetic dispersion. The ARG-MOB scale is proposed which indicates how mobilized detected ARGs are in bacterial genomes. It is concluded that antibiotic efflux genes are rarely mobilized and even 80% of β-lactamases have never, or very rarely, been mobilized in the 15,790 studied genomes. However, some ARGs are indeed mobilized and co-occur with IS elements, plasmids, and integrons.<br><br>Conclusions<br><br>In this study, ARGs in all complete bacterial genomes are classified by their association with MGEs, using the proposed ARG-MOB scale. These results have consequences for the design and interpretation of studies screening for resistance determinants, as mobilized ARGs pose a more concrete risk to human health. An interactive table of all results is provided for future studies targeting highly mobilized ARGs.
Corresponding Author:	Tue Kjærgaard Nielsen, Ph.D.<br>Københavns Universitet<br>Frederiksberg C, Denmark DENMARK
Corresponding Author Secondary Information:	
Corresponding Author's Institution:	Københavns Universitet
Corresponding Author's Secondary Institution:	
First Author:	Tue Kjærgaard Nielsen, Ph.D.
First Author Secondary Information:	
Order of Authors:	Tue Kjærgaard Nielsen, Ph.D.
	Patrick Denis Browne, PhD
	Lars Hestbjerg Hansen, PhD
Order of Authors Secondary Information:	

Powered by Editorial Manager® and ProduXion Manager® from Aries Systems Corporation

Additional Information:	
**Question**	**Response**
Are you submitting this manuscript to a special series or article collection?	No
**Experimental design and statistics**<br><br>Full details of the experimental design and statistical methods used should be given in the Methods section, as detailed in our Minimum Standards Reporting Checklist. Information essential to interpreting the data presented should be made available in the figure legends.<br><br>Have you included all the information requested in your manuscript?	Yes
**Resources**<br><br>A description of all resources used, including antibodies, cell lines, animals and software tools, with enough information to allow them to be uniquely identified, should be included in the Methods section. Authors are strongly encouraged to cite Research Resource Identifiers (RRIDs) for antibodies, model organisms and tools, where possible.<br><br>Have you included the information requested as detailed in our Minimum Standards Reporting Checklist?	Yes
**Availability of data and materials**<br><br>All datasets and code on which the conclusions of the paper rely must be either included in your submission or deposited in publicly available repositories (where available and ethically appropriate), referencing such data using a unique identifier in the references and in the "Availability of Data and Materials" section of your manuscript.	Yes

Have you have met the above requirement as detailed in our Minimum Standards Reporting Checklist?	

Powered by Editorial Manager® and ProduXion Manager® from Aries Systems Corporation

Manuscript

Click here to access/download;Manuscript;ARGMOB_GigaScience_revised

Antibiotic resistance genes are differentially mobilized according to resistance mechanism

Tue Kjærgaard Nielsen[1*], Patrick Denis Browne[1], Lars Hestbjerg Hansen[1*]

[1]Department of Plant and Environmental Sciences, Section for Environmental Microbiology and Biotechnology, University of Copenhagen, Thorvaldsensvej 40, Frederiksberg C 1871, Denmark

*Corresponding authors

Tue Kjærgaard Nielsen

Thorvaldsensvej 40, 1871 Frederiksberg C, Denmark

Phone: +45 30234821

Email: tkn@plen.ku.dk

Lars Hestbjerg Hansen

Thorvaldsensvej 40, 1871 Frederiksberg C, Denmark

Phone: +45 28752053

Email: lhha@plen.ku.dk

ORCIDs:

Tue Kjærgaard Nielsen [0000-0001-6610-8450]; Patrick Denis Browne [0000-0001-8300-7758];

Lars Hestbjerg Hansen [0000-0003-0027-1524];

Abstract

Background

Screening for antibiotic resistance genes (ARGs) in, especially environmental, samples with (meta)genomic sequencing is associated with false-positive predictions of phenotypic resistance. This stems from the fact that most acquired ARGs require being overexpressed before conferring resistance, which is often caused by decontextualisation of putative ARGs by mobile genetic elements (MGEs). Consequent overexpression of ARGs can be caused by strong promoters often present in insertion sequence (IS) elements and integrons and the copy number effect of plasmids which may contribute to high expression of accessory genes.

Results

Here, we screen all complete bacterial RefSeq genomes for ARGs. The genetic contexts of detected ARGs are investigated for IS elements, integrons, plasmids, and phylogenetic dispersion. The ARG-MOB scale is proposed which indicates how mobilized detected ARGs are in bacterial genomes. It is concluded that antibiotic efflux genes are rarely mobilized and even 80% of β-lactamases have never, or very rarely, been mobilized in the 15,790 studied genomes. However, some ARGs are indeed mobilized and co-occur with IS elements, plasmids, and integrons.

Conclusions

In this study, ARGs in all complete bacterial genomes are classified by their association with MGEs, using the proposed ARG-MOB scale. These results have consequences for the design and interpretation of studies screening for resistance determinants, as mobilized ARGs pose a more concrete risk to human health. An interactive table of all results is provided for future studies targeting highly mobilized ARGs.

Keywords: Antibiotic resistance genes, mobile genetic elements, bioinformatics.

Background

Pathogenic bacteria resistant to antibiotics pose an enormous threat to human health, resulting in up to 10 million annual deaths in 2050 if we do not address the issue now, as estimated by the UN Interagency Coordination Group on Antimicrobial Resistance [1]. Health and environmental challenges imposed by antibiotic resistance has sparked enormous research efforts into characterizing genetic resistance determinants. Combined with broad availability of 2nd and 3rd generation sequencing technologies, studying the presence and prevalence of ARGs in the environment has become popular in recent years. Bacteria can become resistant to antibiotics through several genotypic changes including point mutations that lead to either altered gene expression or change of protein function, gene amplifications, genome shuffling leading to increased expression of resistance determinants, and lastly through acquisition of novel genetic material by horizontal gene transfer. The latter transferral of ARGs is especially problematic, as e.g. plasmids carrying ARGs with strong adjacent promoters can be globally spread to important human pathogens. Here, we focus on the genetic context of these acquired resistance determinants and evaluate to what degree they have been mobilized.

Antibiotic resistance remains a significant global issue despite numerous studies into understanding the spread of genes via mobile genetic elements [2,3] and devising mitigation strategies. However, many classes of ARGs are intrinsic to bacterial genomes and can be considered part of the core bacterial genome and may perform non-resistance functions [4–7]. Furthermore, many ARGs have only been shown to provide resistance when cloned into expression vectors [8,9] or with constitutive expression in mutants. Some of these cloned ARGs can hypothetically be transiently highly expressed to confer some level of resistance in their native genetic context, or as mentioned above have mutations in a controlling promoter or repressor gene, leading to high expression. Alternatively, ARGs can become ’decontextualized’ by mobilization, leading to overexpression and

resistance [6,10–14], which moreover can lead to the spread of the decontextualized ARGs via horizontal gene transfer. Examples of genetic events that can lead to mobilization and dissemination of ARGs include I) insertion of proximal IS element with an internal promoter [15] that may lead to the formation of a unit or composite transposon that can carry an ARG as an accessory gene, II) subsequent transfer from chromosomes to (high copy number) plasmids [16], III) capture by- and integration within integrative and conjugative elements (ICEs) [17], and IV) incorporation of an ARG into an integron as a gene cassette [18,19]. This aspect is often overlooked in culture-independent studies using (meta)genomics and/or (q)PCR-based detection where resistance is rarely experimentally verified. The issue is further discussed in a review by Martínez et al [14]. Thus, screening the environment for ARGs may give the impression that “resistance is everywhere” or that widespread resistance predates the use of antibiotics [20–22], although the native roles of ARGs’ homologs may not be related to antibiotic resistance [5,10,23–27]. This topic is subject to debate, with some researchers claiming that all ARGs are suitable targets to screen for in e.g. metagenomes, since they can be potentially decontextualized and ultimately lead to problematic resistance in e.g. pathogens [28] and others warranting that ARGs should be ranked according to how much of a concrete risk they pose [23].

When coupling (meta)genomic predictions with culture-based detection of resistant strains, it is often found that the two approaches do not agree [8,29–32], partially attributable to the fact that gene expression is rarely considered [33]. Screening a genome for resistance markers against an ARG database sometimes results in copious false-positive resistance predictions [30]. This issue can be most pronounced for efflux-related markers where the specificity of prediction has been reported to be 0.12 [34]. The balanced accuracy of resistance marker prediction against two widely used databases was only 0.52 and 0.66, showing that finding ARGs does not necessarily equate to phenotypic resistance [34]. As discussed in a EUCAST report, the resistance genotype-phenotype

concordance has elsewhere been shown to be much higher, with agreements reaching almost 100% in studies that apply subsets of focused and manually curated ARG databases to predict resistance toward selected antibiotics in well-studied species with clinical relevance [35]. In the same report, it is argued that high genotype-phenotype concordances mostly apply to well-characterized (clinical) isolates [35], emphasizing the potential problems in applying large ARG databases to screen for resistance in environmental samples and in less well-studied species. Researchers have devised more advanced machine learning prediction methods to predict resistance in well-characterized bacteria such as *Mycobacterium tuberculosis* with a specificity and sensitivity ranging from 82% to 92.7% [36], in non-typhoidal *Salmonella* with an accuracy of 95% [37], and in *E. coli* with an average accuracy of 91% [38]. These examples highlight that accurate resistance predictions can be made on well-characterized bacteria using curated subsets of well-understood ARGs, but also that predictions on less characterized taxa and in environmental metagenomics using large and unspecific ARG databases is subject to erroneous predictions.

Although low specificity of resistance prediction from efflux-based ARGs has been reported [34], it should be noted that many groups of efflux pumps are inherently encoded, unmobilized, on chromosomes of important human pathogens where they can confer intrinsic resistance, potentially along with other non-resistance functions [7,39]. They may be transiently strongly expressed to confer resistance or overexpressed through mutations in transcriptional regulators [39,40]. The pathogen *Acinetobacter baumanii* serves as a great example of bacteria that host a wide range of efflux pumps, of which some confer intrinsic resistance and others require overexpression. Many of the native *A. baumanii* pumps are involved in non-resistance functions such as membrane composition and stability, opaque/translucent colony phase variation, various stress reliefs, biofilm formation, plasmid transfer rates, natural transformation, quorum sensing, and efflux of dyes, disinfectants, metals, and other non-antibiotic compounds [39]. Expression of efflux pumps are

usually under the regulation of either local and/or global transcriptional regulators and mutations in these regulators are an important path to phenotypic resistance through overexpression in clinical isolates [41]. Besides transient changes in expression or mutations in transcriptional regulator genes, the mobilization of ARGs is an essential aspect of phenotypic resistance.

Most ARGs likely have native roles unrelated to resistance to clinical concentrations of antibiotics [23]. Many antibiotics are natural secondary metabolites, occurring at clinically insignificant concentrations, that are involved in inter-cellular communication, regulation of metabolism, and other non-resistance functions [29]. ARGs have been found in and cloned from susceptible bacteria [8], where they are simply performing their original non-resistance roles. Previous studies have shown that e.g. efflux pumps [10,24], β-lactamases [25,26], and lipid A modifying proteins (MCR) [27] have non-resistance functions, although the genes encoding these may be decontextualized to confer resistance.

Functional (meta)genomic approaches, essentially cloning fragmented DNA into expression vectors followed by screening for e.g. antibiotic resistance [4,9,21,22,43,44], has led to the identification of many putative ARGs [44–50]. Such genes are decontextualized in the experimental setup and their native roles may not be related to resistance. This has resulted in a problematic dissemination of self-reinforcing resistance-related annotations in gene databases. Thus, sequence homology is a poor proxy for resistance and culture-independent techniques will often yield misleading results if genetic contexts of ARGs are not considered. With recent advances in long-read sequencing, high-quality metagenome-assembled genomes can be derived[51], facilitating consideration of the genetic context of ARGs.

The associations between ARGs and MGEs are important [16,52,53] and have profound effects on phenotypic resistance [11,18,54–56]. It has been argued, e.g. in the “RESCon” framework [57], that

multiple aspects, including genetic context, should be included in risk assessment of ARGs [58,59]. Here, we initiate the route to more accurate ARG predictions by categorizing associations between ARGs and MGEs in all completed RefSeq bacterial genomes. We hypothesize that highly mobilized classes of ARGs represent those that are infamous for causing phenotypic resistance and were furthermore initially characterized from already resistant clinical isolates. On the other hand, we also hypothesize that ARGs with low degree of mobilization are represented by ARG classes that were initially identified through shotgun cloning from non-resistant isolates and subsequently only shown to confer resistance through overexpression from a plasmid vector. This does not rule out future mobilization events and subsequent elevation of the risk posed by a yet unmobilized ARG. Decontextualization of ARGs is explored by examining their association with I) plasmids, II) IS elements, III) integrons, and IV) their dispersal across distinct genera. We collect this information per class of ARG in the unifying ARG-MOB scale for mobilization of resistance genes in all complete RefSeq bacterial genomes. Among other results, we conclude that most classes of antibiotic efflux genes are rarely mobilized from their original, chromosomal location and that even 80% of classes of β-lactamases have never or very rarely been mobilized. This necessitates both increased awareness of the genetic context of ARGs but also more critical choosing of ARG targets for future, especially environmental, studies.

Data Description

The CARD database was used to find ARGs in all completed bacterial genomes from the RefSeq database (n=15,790). Then, 12,170 bp up- and downstream of predicted ARGs were analysed for IS elements and integrons, while replicon type (plasmid or chromosome) was also considered. For more details, see Methods and Supplementary Information (Supplementary Text 1, Supplementary Figs. 1-4; Supplementary Table 1). All databases are assumed to be biased, especially towards

human-associated bacteria of which many almost identical genomes have been uploaded to RefSeq, leading to overrepresentation of these compared to e.g. environmental bacteria (Supplementary Text 2, Supplementary Figs. 5 and 6). In order to ameliorate these biases, highly similar genetic loci with predicted ARGs (n=176,888) were clustered to 53,895 Clustered Resistance Loci (CRLs), representing 1,176 Antibiotic Resistance Ontology (ARO) terms from CARD (Fig. 1). We compared the frequency of genera found in both CARD and RefSeq and calculated the Euclidean distance of these frequencies before and after clustering to CRLs. The Euclidean distance of genera frequencies was reduced from 30.89 to 10.26, showing that many ARG loci in RefSeq are highly similar (Supplementary Text 3, Supplementary Fig. 7). Four mobilization parameter ratios were explored for each CRL (Fig. 2): I) replicon type, II) IS element-association, III) integron-association, and IV) dispersal of CRL across genera (Simpson diversity). All parameters were calculated on a scale of 0-1, with 1 indicating that a CRL is always associated with the given parameter. The mean of the four ratios is termed the ARG-MOB score and indicates how much genes of a given ARO are mobilized. This is described further in later sections and Methods (Supplementary Fig. 8). Prophages in genomes are not explored for ARGs, since these are not common vectors [60]. Neither are ICEs explored, although they are important in resistance development [17], since they are underexplored and likely difficult to predict across the wide phylogenetic array of genomes studied here. Likewise, bacteriophages represent an astounding reservoir of genetic diversity [61] and successful prediction of integrated prophages is likely extremely limited to those occurring in well-studied bacterial families. Including ICEs and prophages here would introduce severe biases in the analyses and they are thus excluded.

Analyses

The 16S rRNA gene as a non-mobilized control

The average length of 449 composite and unit transposons in The Transposon Registry [62] was calculated to 12.17 kbp (Supplementary Fig. 4). This distance was used to screen for the presence of IS elements and integrons in both directions from identified ARGs. The 16S rRNA gene was used as a non-mobilized control, as this gene is not expected to be associated with MGEs (Supplementary Text 4). Only 5.59% of 16S rRNA genes in the 15,790 complete genomes are within 12.17 kbp of an IS element (Supplementary Fig. 9). Since 16S rRNA genes should be extremely rarely associated with e.g. transposons, we consider the 5.59% as a proxy confidence interval for false-positive ARG-IS associations.

Efflux-associated ARG loci are less unique than other mechanisms

Loci with efflux-associated ARGs were more compressed by clustering to CRLs than all other types (Mann-Whitney U-test (MWU) $p_{adj} < 0.0001$; Supplementary Fig. 7), indicating that these are more conserved and contain less variation from e.g. mobilization events. The *antibiotic efflux* (*efflux*) mechanism is the most abundant category and its CRL count is more than two times more numerous than the second-largest category, *antibiotic inactivation* (*inactivation*), although *inactivation* has over 3 times as many AROs as *efflux* (Fig. 1). As expected, loci in human-associated genera were especially compressed by clustering, showing that these are indeed overrepresented in the RefSeq database (Supplementary Fig. 7).

Association of ARGs with IS elements and plasmids

Major resistance mechanisms (non-hybrid) are associated with IS elements and plasmids to varying degrees (Fig. 3) and were associated with different families of IS elements (Supplementary Text 5, Supplementary Fig. 10). *Efflux* AROs generally have very low IS and Replicon ratios, which indicates that *efflux* ARGs are rarely mobilized by either IS elements or plasmids. Only few *efflux*

AROs have both high IS and high Replicon ratios, including ARO3002693 (transposon-encoded *cmlA1* chloramphenicol exporter), ARO3003836 (*qacH* subunit of fluoroquinolone exporter), and ARO3000165 (tetracycline efflux pump *tetA*). Many *efflux* AROs contain a large number of unique CRLs, as is also reflected by *efflux* CRL count in Fig. 1. Therefore, *efflux* ARGs are rarely associated with either IS elements or plasmids. Distances, in terms of nucleotides, between ARGs and IS elements are larger for *efflux* AROs than for other mechanisms, indicating that *efflux* ARGs are more “loosely” associated with IS elements than other mechanisms (MWU test $p_{adj} < 0.014$; Supplementary Text 6, Supplementary Fig. 11). Contrary to *efflux*, the *inactivation* mechanism has many AROs that have been mobilized by both IS elements and plasmids, but also AROs that are hardly mobilized at all (Fig. 3). With some exceptions, *antibiotic target alteration* (*target alteration*) AROs have low IS and Replicon ratios while also exhibiting a low number of unique CRLs, indicating that *target alteration* CRLs are conserved and often not decontextualized. On the other hand, *antibiotic target replacement* (*target replacement*) AROs are more mobilized by IS elements and plasmids (Fig. 3).

ARGs are more decontextualized in clinically relevant genera (Fig. 4, Supplementary Figs. 12 and 13). As expected from database biases (Supplementary Figs. 5-7), Proteobacteria harbour 88.18% of unclustered ARG loci (Supplementary Fig. 2). Although likely to be an artefact of selective sampling, Proteobacteria have been proposed to be the confirmed origin taxa of many acquired ARGs, although only an estimated 4% of ARGs have known bacterial origins [2]. Proteobacteria have a higher median IS ratio than Actinobacteria and Bacteriodetes (MWU test; $p_{adj} < 0.01$). Within Proteobacteria, *Enterobacteriaceae* have a higher median IS ratio than *Campylobacteriaceae,* and *Burkholderiaceae* but lower than *Aeromonadaceae, Pasterurellaceae* and *Morganellaceae* (Fig. 4). However, CRLs in *Enterobacteriaceae* are more often found on plasmids than for *Campylobacteraceae*, *Moraxellaceae*, *Morganellaceae, Neisseriaceae*,

Pasteurellaceae, *Pseudomonadaceae*, and *Burkholderiaceae* (Supplementary Fig. 12; MWU test; $p_{adj} < 0.01$), showing that many ARGs in *Enterobacteriaceae* are highly mobilized by both IS elements and plasmids. Within *Enterobacteriaceae*, many ARG loci have been mobilized both by IS elements and plasmids, especially within the genera *Shigella*, *Escherichia*, *Salmonella*, *Klebsiella*, *Enterobacter*, and to a lesser degree *Citrobacter* (Fig. 4). Other genera in *Enterobacteriaceae* show lower median mobilization degrees (Significance values in Supplementary Table 3). *Enterobacteriaceae* genera with highly mobilized ARGs all have members of significant importance to human health and persistent fixation of mobilized ARGs are likely a consequence of human interference with pathogenic bacteria [54].

There are significant differences between the median IS ratios of phyla with e.g. Actinobacteria and Bacteriodetes having lower median IS ratios than Proteobacteria (MWU test; $p_{adj} < 0.01$). Within Firmicutes, whose IS ratio is not different from that of Proteobacteria, some families are associated with human activities such as *Enterococcaceae* and *Staphylococcaceae*. These harbour highly mobilized ARGs, while environmentally associated Firmicutes, such as *Bacillaceae*, have many ARGs barely mobilized by either IS elements or plasmids (Supplementary Fig. 13). This exemplifies how homologs of ARGs can be found in both environmental and clinically relevant genera, but that they have been decontextualized more in the latter. It should be noted that the *Bacillaceae* family has many members associated with humans, including the human gut. However, the “isolation source” and “host” modifiers for the downloaded RefSeq genome entries are severely lacking, making it impossible to meaningfully discern environmental from human-associated strains. As an example, only 16.5% of genome entries have “host” information with just 2.5% of that being “Homo sapiens” and only 30.9% have “isolation source” information with the largest source being “soil” at 13.2%. This supports our assumption that a majority of *Bacillaceae* genomes are from environmental strains.

Generally, diving into specific families and genera shows that ARGs in human-associated bacteria are more mobilized than in others (Supplementary Table 3) and fixation of mobilized ARGs are likely a consequence of human interference with pathogenic bacteria [54]. This selection stemming mainly from antibiotic usage is obviously hugely important in the fixation of ARGs in context of MGEs.

Integron-association varies across ARG classes

Under selective pressure for resistance, ARGs may be decontextualized into integrons, where a strong promoter confers overexpression of said ARGs, that may result in phenotypic resistance [19]. Using IntegronFinder [63] on CRL sequences, 3,723 ARGs were identified as gene cassettes in integrons or clusters of *attC* sites lacking integron-integrases (CALIN). The most abundant major mechanism was *antibiotic inactivation* with 2,684 unique CRL occurrences. Mechanisms *antibiotic target replacement* and a*ntibiotic efflux* were found in association with integrons in 694 and 310 CRLs, respectively (Supplementary Fig. 14). At first glance, the sulfonamide resistance genes associated with Tn*402* Class 1 integrons [19], *sul1-4,* were not the most frequent submechanism associated with integrons and was here found associated with integrons in only 123 unique CRLs out of 2,017 total *sul1-4* CRLs. However, the *sul1* gene associated with Class 1 integrons is found as a conserved segment in the 3' part of the integron and does not have its own *attC* site and is therefore often missed by IntegronFinder. In fact, complete integrons and/or CALINs were found in proximity of *sul1* genes (ARO:3000410) in 1,393 out of 1,527 unclustered regions, supporting the well-described association between Class 1 integrons and *sul1* [19]. Trimethoprim resistance *dfr* genes associated with Class 2 integrons and Tn*7* transposon [19] were here found in high abundance in association with integrons. The most abundant submechanism was the "*inactivation" ant(3'')* category, whose genes encode aminoglycoside nucleotidylylating enzymes, with 1,009

CRLs associated with integrons. The *ant* genes are often found in association with integrons [64. Here, the 5 *ant* AROs *aadA, aadA2, aadA5, ant(3")-IIa,* and *ant(2")-Ia* all display integron associations in at least 54.24% of their total CRL occurrences (Supplementary Table 4). Similarly, the aminoglycoside acetyltransferase-encoding *aac(6’)* AROs (*aac(6’)-Ib-cr, aac(6')-Ib10, aac(6')-Ib7,* and *aac(6')-Ib9*) are here mostly found in integrons, agreeing with previous description of this class of ARGs [64]. Genes encoding OXA-9 and OXA-1 β-lactamases are found in integrons in 98.51% and 63.90% of the 67 and 277 CRLs, respectively, emphasizing that these ARGs are of concern (Supplementary Table 4).

From these results, it is evident that some target ARGs are highly associated with integrons and are thus more relevant to screen for in an environment with e.g. PCR or sequencing, if the aim is to predict phenotypic resistance. This again emphasizes the importance of considering the genetic contexts of ARGs.

Mobilization assessment based on four parameters

Inspired by previous work [23], we calculated a mobilization scale for each ARO, termed the ARG-MOB scale, which is calculated as the mean of the four mobilization parameters (MOB) and ranges from 0 to 1 with 1 representing very high mobilization, signified by very high IS and plasmid ratios, frequent association with integrons, and a wide phylogenetic dispersal across genera. Fig. 5a shows the MOB parameters and ARG-MOB scale for each ARO. For each MOB parameter, boxplots with MWU test results are also shown (Fig. 5b).

The median ARG-MOB per major mechanism is highest for *antibiotic target replacement* (p_{adj} < 0.0001), while *antibiotic efflux* has a low median ARG-MOB but not significantly different from *antibiotic target alteration* and *antibiotic inactivation* groups. *Antibiotic target protection,*

antibiotic inactivation, and *antibiotic target alteration* groups are not significantly different (Fig. 5a).

Efflux genes are rarely mobilized

The *efflux* mechanism has the lowest median ARG-MOB (although only significantly lower than *target replacement* and *target protection*, which is reflected by median Replicon, IS, and Integron ratios that are lower than most other groups (Fig. 5b), i.e. efflux genes are rarely mobilized by these MGEs. It is therefore likely that most identified efflux ARGs are part of core bacterial genomes located in conserved loci of chromosomes, with a few highly mobilized exceptions (Fig. 5a). This supports previous conclusions on efflux pump genes [7,10,39,40]. While transient changes in expression or overexpression through mutations in transcriptional regulators may confer phenotypic resistance through extrusion of antibiotics, we advocate that genetic context needs to be considered, when screening environments for efflux-associated ARGs. In clinical settings, the transient or constitutive expression of efflux pumps still warrant emphasis on screening for efflux-associated resistance markers.

The highest median ARG-MOB mechanism, *antibiotic target replacement*, is characterized by AROs with a high degree of mobilization by IS elements and plasmids (Fig. 5). The high ARG-MOB *target replacement* AROs are furthermore strongly associated with integrons and are taxonomically more widespread than *target alteration*, *inactivation*, and *efflux* groups (Fig. 5b). Likewise, some *target protection* AROs are highly mobilized and widespread but they are to a lesser degree associated with integrons. Generally, *target replacement* is significantly more associated with integrons than other categories, although the median of *inactivation* is higher than *target alteration* and *efflux*.

AROs of *inactivation* mechanisms are the least phylogenetically dispersed but are instead conserved within few genera, as indicated by the lowest median Simpson index. Possibly, many genes and/or proteins under the *inactivation* mechanism only function in specific genera, whereas those of other mechanisms can function in wider ranges of genera. While there are *inactivation* AROs that have been mobilized by plasmids, transposons, and integrons, there are many others that have not been decontextualized (Fig. 5a). All major mechanisms have exceptions in the form of AROs with elevated ARG-MOB, as evaluated on all four parameters, although *target alteration*, *target protection*, and *efflux* have few or no AROs with ARG-MOB higher than 0.75.

The ARG-MOB scale proficiently describes decontextualization of ARGs

The four MOB parameters all correlate significantly with each other, showing that they covary and are appropriate for calculating the ARG-MOB scale (Fig. 6a and b). Hierarchical clustering of AROs from all five major mechanisms in a heatmap shows apparent mechanism-specific profiles of ARG-MOB scores, as well as each of the four MOB parameters (Fig. 6c). Two major branches are formed from clustering: I) a high-ARG-MOB branch dominated by *inactivation*, as well as other individual AROs from other mechanisms and II) a low-ARG-MOB branch mostly populated by *efflux* AROs.

The highest correlation coefficient is seen for IS-Replicon ratios, showing that ARGs placed on plasmids are likely mobilized by IS elements prior to insertion on plasmids (Fig. 6a and b). The second highest correlation is found between IS and integron ratios, indicating that ARGs, found as gene cassettes in integrons, are likely to have been mobilized (as part of integrons) by IS elements (Fig. 6a and b) which has been often reported and discussed [18,19,55]. To a lesser degree, ARGs found on plasmids are correlated with integrons.

Not surprisingly, the Simpson diversity index correlates positively with IS, replicon, and integron ratios (Fig. 6a), showing that highly mobilized ARGs are also likely to be phylogenetically widespread. On the basis of these correlations, we conclude that the ARG-MOB ratio proficiently describes decontextualization of ARGs. Pearson correlation coefficients and MGE co-occurrences were also calculated per mechanism (Supplementary Text 7, Supplementary Figs. 15-20).

Some AROs are highly divergent in mobilization

Many AROs can be defined as either highly mobilized or only to a very small degree. Still, some AROs have a large spread from their mean ARG-MOB score, showing that they are most often sitting unmobilized on a chromosome, but have one or times been mobilized and widely dispersed (Supplementary Figs. 21 and 22). This is exemplified by the efflux pump genes *oqxAB* (ARO3003922-3) [65,66] (Supplementary Text 8). These genes are found on essentially all *Klebsiella pneumoniae* chromosomes where they do not confer resistance unless overexpressed [67–69], as seen when placed close to IS elements on plasmid pOLA52 [65]. The *oqx* AROs show high spread across their mean IS and Replicon ratios (*oqxA* has IS and Replicon ratios of 0.35 and 0.16, respectively), showing that their mean ratios are low due to *Klebsiella* chromosomes but that there are many outliers due to variants in *Escherichia* and *Salmonella* that are only found mobilized by IS elements and usually on plasmids (Supplementary Fig. 21, Supplementary Table 5). Outliers from the mean of IS and Replicon ratios can also be considered per genus instead of ARO, in order to highlight that ARGs in some genera are much more mobilized than in others. For example, efflux pump genes in *Shigella* are more associated with IS elements compared to the global average, but they are not found on plasmids more than on average (Supplementary Fig. 22). Likewise, many *antibiotic inactivation* ARGs are found more on plasmids in *Escherichia, Salmonella*, *Klebsiella*, *Citrobacter*, and *Enterobacter* than their respective average placements per

ARO. Other genera including *Proteus*, *Pseudomonas*, *Acinetobacter*, and *Morganella* tend to have some *inactivation* ARGs more located on chromosomes than the given ARO average, indicating that chromosomes in these genera may be considered reservoirs of potential genes with potential as resistance determinants. This highlights the complexity of the ARG issue and emphasizes the importance of considering the genetic context before predicting resistance.

Defining ARG-MOB categories

Smoothed kernel density estimates of AROs and their ARG-MOB values are shown in Fig. 7a per mechanism and cumulatively for all mechanisms. The following five ARG-MOB groupings were defined computationally: *Very low* (ARG-MOB = 0), *Low* (0 < ARG-MOB < 0.182), *Medium* (0.182 < ARG-MOB < 0.378), *High* (0.378 < ARG-MOB < 0.681), *Very high* (ARG-MOB > 0.681). These definitions are largely the same when estimating per mechanism individually (Supplementary Fig. 23). Numerically, *inactivation* has the highest number of *High* and *Very high* ARG-MOB AROs (144 and 54, respectively), while *target replacement* has the highest percentage of *High* and *Very high* ARG-MOB AROs with these categories representing 64% of *target replacement* AROs (Fig. 7b).

***High* ARG-MOB AROs correspond with high-risk ARGs**

High and *Very high* ARG-MOB AROs (Fig. 7c) are mainly ARGs that were initially identified in resistant pathogens where they indeed confer resistance. Conversely, many low ARG-MOB AROs have only been shown to confer resistance when placed on high-expression cloning vectors but not in any natural wild-type isolate. A few examples are described below and in Supplementary Text 9. A table for all 1,176 AROs can be found as an interactive table in Additional file 1.

For all major mechanisms, many AROs are classified as *Very low* or *Low* ARG-MOB (Fig. 7c) and *antibiotic target alteration* does not have any *Very high* ARG-MOB AROs, while *target protection* has two (ARO3002803 and 3002801; quinolone resistance genes *qnrVC6* and *qnrVC4*).

Inactivation has many AROs with *High* and *Very high* ARG-MOB, which include infamous β-lactamases, aminoglycoside nucleotidyltransferases (ANT), and others (Fig. 7c). Aminoglycoside resistance by *antibiotic inactivation* is highly represented by ARGs scoring *High* and *Very high* on the ARG-MOB scale, showing that aminoglycoside resistance is in many cases highly mobilized (Supplementary Fig. 24). Likewise, ARGs encoding resistance to up to five groups of β-lactam antibiotics (carbapenem, cephalosporin, cephamycin, penam, and penem) are highly mobilized, highlighting the critical state of resistance towards these antibiotics (Supplementary Fig. 24). With a median ARG-MOB of 0.73, the gene encoding the Verone integron-encoded metallo-β-lactamase (VIM) is the β-lactamase gene with the highest median ARG-MOB. There are three VIM β-lactamase AROs, of which ARO3002271 has the highest ARG-MOB of any *inactivation* ARO at 0.91. In RefSeq complete genomes, the gene is only found inserted in integrons, and is located close to IS elements and on plasmids in 95% of the CRLs found (n=21). It is dispersed across 6 unique genera for a Simpson index of 0.75 (*Pseudomonas*, *Salmonella*, *Escherichia, Klebsiella*, *Citrobacter*, and *Enterobacter*). The VIM-1 gene was found in a multiresistant *E. coli* from a patient. It was inserted in a Class 1 integron and found on a conjugative plasmid [70]. It has since been seen in multiple *Enterobacteriaceae*, typically in association with MGEs, and is globally spread [71].

The highest ARG-MOB *target replacement* AROs belong to the trimethoprim resistant dihydrofolate reductase *dfr* submechanism. The ARO3003013 within this submechanism has the highest ARG-MOB of any ARO at 0.95. A Class 1 integron with *dfrA15* is widespread in *Vibrio cholera* isolates in Africa and was found on a conjugative plasmid [72]. It is the ARO with the

highest ARG-MOB, since it was only found to be associated with IS elements, integrons, and plasmids (all ratios = 1). It has a Simpson index of 0.82 and the 7 CRLs are dispersed across 6 genera (*Vibrio, Salmonella*, *Enterobacter*, *Leclercia*, *Klebsiella* and *Escherichia*). Generally, ARGs conferring diaminopyrimidine (including trimethoprim), resistance by *antibiotic target replacement* mechanism are highly mobilized and numerous in the complete genomes studied here (Supplementary Fig. 24).

Discussion

The ARG-MGE association aspect has received much attention recently [2,3,18]. For instance, it was shown that mobilized ARGs often have confirmed origins in Proteobacteria, especially from human- and animal-associated species, although the confirmed origins have only been found for an estimated 4% of ARGs [2]. This is likely due to selective pressure for resistance in these environments that make them ARG mobilization hotspots [2]. This is supported in our work where 88.18% of ARG loci are found in Proteobacteria but is also elaborated by our finding that Proteobacteria is not only the origin of many ARGs but also harbour the bulk of mobilized ARGs, especially within the *Enterobacteriaceae*. Of course, database biases (Supplementary Text 2) strongly influence the findings presented here and elsewhere [2].

The publications described above were part of our inspiration for the work in this study which expands on the subject of ARG-MGE associations. We have applied a similar context-focused approach as these [2,3], although we here, with empirical evidence, investigate a distance of 12.17 kbp from ARGs rather than the distances of 10 kbp or 10 ORFs in the other studies. Furthermore, the threshold for including ARG matches is more stringent in our study at 80% similarity and 80% query coverage, compared to the other studies with 90/50% [3] and 70/80% [2], respectively. The two referenced papers are from the same research group and are published less than 6 months apart,

highlighting that there is no consensus strategy for finding ARG homologs, although a standardized approach is sorely needed. The same research group published in 2019 the tool fARGene [73] which applies curated Hidden Markov Models to make ARG predictions. While the sensitivity is surely higher with such model-based approaches, they are severely limited by the number of available models which, at the time of writing, are not sufficient for large-scale studies such as that presented here.

It is well established that mobile ARGs pose a more concrete threat [23], although it has been argued that all ARGs, irrespective of whether they have ever been mobilized or found in a clinical isolate, should be considered a potential threat [28]. With the astounding sizes of current ARG databases in the thousands of genes, surely there must be ARGs that pose a bigger threat than others? The ARG-MOB score presented here is our approach for identifying target ARGs that have been mobilized in bacterial genomes.

Our results clearly demonstrate the importance of including the genetic context in ARG predictions, since even the highest ARG-MOB scoring genes have representatives that are not decontextualized and may not confer resistance. This study documents how genes from even the most mobilized categories of ARGs can be found unmobilized on chromosomes. Therefore, the validity of using PCR-based screening to assess the abundance and distribution of putative ARGs is questionable at best, unless context is likewise included in PCR design, as seen before [74] and suggested elsewhere [3]. Including this aspect in future studies may help to alleviate the occasional discrepancies between genotypic and phenotypic resistance predictions [8,29–32], with especially efflux-related markers producing a high number of false-positive predictions [34]. Even the presence of β-lactamase genes cannot solely be used as predictors of resistance, as they are involved in regular cell upkeep [25] and are in this study found to not have been mobilized in 80% of their genomic occurrences (see Additional file 1). It should be noted that other studies did find high

congruence between genotypic and phenotypic resistance [35–38], using well-known human pathogens and subsets of curated ARG databases. This highlights that using very large ARG databases to screen non-clinical environments for resistance markers is likely to result in false-positive resistance predictions.

Based on examples of high and low ARG-MOB AROs (Supplementary Text 9), a pattern emerges that high ARG-MOB AROs, such as the *bla(VIM)* [70], *dfrA15* [72], *aac(6')* [75] and *arr-2* [76], that were originally identified in already virulent, pathogenic bacteria that had indeed been verified to be resistant. On the other hand, low ARG-MOB AROs were generally identified in susceptible bacteria and/or only shown to cause resistance when cloned into vectors with strong gene expression, such as *murA* [45], *norB* [46], and *bla(CME-1)* [48] (Supplementary Text 9). This warrants caution when choosing ARGs of interest in either targeted (q)PCR screening or metagenomic sequencing of environmental samples. We advocate that knowledge of ARG mobilization paired with other factors, such as trends in antibiotic usage [77], will allow us to better understand ARGs of concern and to predict future problematic resistance determinants. A worrying aspect in our results is the extent to which several classes of genes encoding broad-spectrum β-lactamases are found to be highly mobilized (Fig. 7), coupled with the fact that penicillins and other β-lactams have seen a great increase in global usage in recent years [77].

Potential Implications

Based on the results presented here and as discussed elsewhere [11,23,57], it is clearly necessary to consider the genetic context of genes when predicting ARGs from (meta)genomes. This could be achieved by applying PCR primers that target regions spanning both an ARG and an associated MGE [74]. For more accurate ARG calling, metagenomic sequencing using long-read platforms is a prerequisite to enable the detection of ARGs and their genetic contexts. For more targeted

investigations where a "meta" approach is either not feasible or within scope, we provide a comprehensive and interactive table of the results presented in this study (see Additional file 1). This table can be used as a tool to select more relevant possible resistant determinants in future studies. We are strong proponents of more focused and accurate predictions of true ARGs, especially when dealing with environmental samples, as it is vital that the serious resistance issue is managed and discussed with diligence and precision.

Methods

Databases and ARG prediction

All code for data processing was written in BASH scripts and statistics and plotting were primarily done in RStudio.

All complete bacterial genomes (15,790 entries with 16,785 chromosomes and 14,280 plasmids) were downloaded from RefSeq on Dec. 12 2019 using the ncbi-genome-download tool v0.2.11 [78]. In order to ensure uniform prediction of genes across all bacterial genomes, Prodigal [79] (v2.6.3; RRID:SCR_011936) was used to predict genes from nucleotide sequences and write corresponding amino acid sequences from all RefSeq genomes. Since Prodigal first trains itself based on the input sequence, gene prediction was performed on subsets of each genus present in RefSeq genomes. Per genus, two rounds of Prodigal were performed with the -meta flag enabled in the second run, as it predicts some genes that are missed in single genome mode and vice versa. Results from the "single" and "meta" gene predictions were consolidated to omit redundancy. Several ARG databases and tools for predicting ARGs have been produced, including CARD [80], ARDB [81], MEGARes [82], ResFinder [83], SARG [84], ARG-ANNOT [85], DeepARG-DB [86], ARGminer [87], FARME [88], and others. Some are discontinued while others receive updates

occasionally. The CARD database is large, actively updated, well-curated, and widely used. Furthermore, it makes use of ontology terms (Antibiotic Resistance Ontology: ARO) that allow for the grouping of resistance genes according to resistance mechanisms. Because of these advantages over other databases and the essential role of ontology terms, the CARD database was used in this study. The "protein homolog" models from CARD were used here, since they do not contain resistance determinants that are based on mutations. The main resistance mechanisms defined in the CARD database are "*antibiotic efflux*" (*efflux*), "*antibiotic inactivation*" (*inactivation*), "*antibiotic target alteration*" (*target alteration*), "*antibiotic target protection*" (*target protection*), "*antibiotic target replacement*" (*target replacement*), and the less abundant "*reduced permeability to antibiotic*" (*reduced permeability*). A few additional categories exist that are hybrids of two of the above mechanisms, but there are very few entries of these in CARD and are for most of the analyses not considered.
The CARD database (v3.0.7) was downloaded and only the protein homolog model was used in this study, excluding resistance determinants related to sequence variants (e.g. SNPs). DIAMOND [89] blastp (BLASTP, RRID:SCR_001010) was used to identify ARGs in all RefSeq genomes. For blastp against the CARD database, both query and subject coverages were set to a minimum of 80%, while E-value cutoffs were set to 1e-10, to limit the rate of spurious hits. For each query protein from all RefSeq genomes, only the single best CARD match was kept.

The CARD auxiliary tool, RGI [90], for predicting ARGs in (meta)genomes uses curated blastp bitscore cutoffs unique to every ARG protein in the CARD database. The same bitscore cutoffs were applied here, with the exception that hits with bitscores lower than the RGI cutoff were included if they had an identity score and a query coverage of at least 80%. These hits were included in order to keep more ARG hits from environmental bacteria that are not clinically

relevant, since it is assumed that CARD and other ARG databases are biased towards genes that reside in anthropogenically relevant strains. Blastp hits with bitscores above the RGI cutoff were also only kept if query coverage was at least 80%. The effects of these filters are further described in Supporting Information (Supplementary Text 1, Supplementary Figs. 1-3).

Extracting the genetic context of ARGs

The average length of composite and unit transposons were calculated based on 449 entries in the Transposon Registry [62]. This average (12.17 kbp) was used as the maximum allowed distance between an ARG and an IS element for classifying an association (Supplementary Table 1). However, since ARGs in transposons can be on either strand relative to the transposase, IS elements are identified within 12.17 kbp of an ARG in both directions. This enables searching for transposons of up to 24.34 kbp (plus the length of the identified ARG), which would include 77.73% of the 449 composite and unit transposons in The Transposon Registry [62] (Supplementary Fig. 4).

For all filtered blastp ARO hits, up to 12,170 bp both up- and downstream of the hit were extracted from the respective RefSeq replicon using the faidx command from Samtools [91,92] (v1.9-166-g74718c2; RRID:SCR_002105). If an ARG was found within 12.17 kbp of either terminus of a replicon, only sequence until the terminus was extracted and not continued from the other end of the sequence, since entries in RefSeq complete genomes may not be actually complete, due to low sequencing coverage regions stemming from e.g. GC-bias in sequencing [93]. Loci were categorized according to the ARO of the identified ARG. There are 9 ARO major mechanism categories of which three are less abundant “hybrids” merged by two other categories. The 6 non-hybrid categories *efflux*, *inactivation*, *target alteration*, *target replacement*, *target protection*, and

reduced permeability are here considered the main categories and are the ones mainly investigated in this study. The mechanism *reduced permeability* is only represented by three ARO categories and is excluded from some statistical analyses.

IS elements in ARG loci and 16S rRNA as control

IS elements in ARG loci were predicted using DIAMOND blastp against the ISfinder database [94], as implemented in Prokka [95] (v1.14.0; RRID:SCR_014732). The same E-value cutoff for IS annotations, as Prokka applies during gene annotation (1e-30), was used here and the minimum query coverage accepted was 90%. Only the top IS hit for each query protein was kept, since multiple “good” hits to distinct IS families may occur per query. The distance between a given ARG and its closest IS neighbour within 12.17 kbp in either direction (if any) was calculated without considering the coding strand of the genes. ARGs not within 12.17 kbp of an IS element were not considered when calculating the mean ARG-IS element distances.

Since 16S rRNA genes are not expected to be often mobilized by IS elements, the distance between 16S rRNA genes and IS elements were explored in all complete RefSeq bacterial genomes, in order to assess how many “false-positive” ARG-IS associations are expected to be identified using the 12.17 kbp distance cutoff (Supplementary Text 9). 80,141 16S rRNA genes in 15,790 strains were predicted using barrnap [96]. Of these, 94.61% did not have identified IS elements within 12,170 bp in either direction, which can be seen as analogous to a 95% confidence interval for predicting association between ARGs and IS elements.

Clustering ARG loci to remove redundancy

Extracted loci with ARGs were grouped based on the CARD ARO category of the loci ARGs. In order to remove redundancy from the RefSeq database, stemming from overreprentation of e.g.

almost identical *E. coli* chromosomes, extracted loci were clustered with USEARCH [97] (v11.0.667_i86linux64) (Supplementary Texts 2-3, Supplementary Figs. 5 and 6). Per ARO group, sequence loci were clustered into what we refer to here as Clustered Resistance Loci (CRL) using the "-cluster_fast" command with the criteria that sequences in a cluster are at least 99% similar over at least 90% of the length (both target- and query coverage) and only the single best hit was allowed per sequence. The "-sort length" flag was also enabled to sort loci by length before clustering, since loci vary in length (sum of 12.17 kbp up- and downstream plus an ARG of varying length). This ensures that loci of identical length (with the exact same ARG) are merged into the same CRLs. For each CRL, the centroid sequence was used as representative sequence for downstream analyses.

Integron prediction

Integrons and cassette arrays were predicted using IntegronFinder [63] using the centroid CRL sequences as input. IntegronFinder can predict complete integrons including gene cassettes, In0 elements where only integrase is present, and CALINs (Cluster of *attC* sites Lacking Integrase nearby). All three classes of integrons are included in the analyses and no distinction is made, since an ARG observed in e.g. a CALIN has been previously associated with an integron and may still be in related, but not sequenced, strains.

Statistical Analysis

Data tables were imported into R for statistics and visualization using the packages ggplot2 (ggplot2, RRID:SCR_014601), dplyr (dplyr, RRID:SCR_016708), tidyr (tidyr, RRID:SCR_017102), gridExtra, ggpubr (ggpubr, RRID:SCR_021139), ggExtra, reshape2, knitr (knitr, RRID:SCR_018533), kableExtra, vegan (vegan, RRID:SCR_011950),

PerformanceAnalytics, ComplexHeatmap (ComplexHeatmap, RRID:SCR_017270), RColorBrewer (RColorBrewer, RRID:SCR_016697), DT, rstatix (rstatix, RRID:SCR_021240), tidyverse (tidyverse, RRID:SCR_019186), broom, and plotly (Plotly, RRID:SCR_013991). Prior to pairwise *post-hoc* tests, all datasets were tested for whether samples originate from the same distribution using non-parametric Kruskal-Wallis tests. Significance was observed for all datasets, allowing for pairwise *post-hoc* tests. Subsequently, statistical tests on rank-sums of groupings were performed with unpaired Mann-Whitney U-tests (MWU) with Holm-Bonferroni correction for multiple testing, since this method does not require independence. All reported p-values are Holm-Bonferroni corrected. Pairwise correlation analyses between MOB parameters were calculated with Pearson correlation coefficients and significance tested with the R function cor.test. A clustered dendrogram of clustering of the AROs, based on the four MOB parameters, was calculated using standard parameters in the 'ComplexHeatmap' package (complete hierarchical clustering on Euclidean distances).

MOB metrics and the ARG-MOB scale

Four main mobilization (MOB) metrics (Fig. 2), or ratios (0 to 1), of mobilization were calculated per ARO that aim to quantify just how mobilized groups of ARGs are. These four ratios are I) the Replicon ratio II) the IS ratio III) the Integron ratio and IV) the phylogenetic spread of an ARO across distinct genera, quantified by the Simpson diversity index. Pearson correlation coefficients between MOB metrics were calculated.

For each ARO category, the number of CRLs with and without identified IS elements were counted and the IS ratio was derived where an IS ratio of 1 indicates that all CRLs belonging to a given ARO have an IS element within 12,170 bp either up- or downstream of the ARG. Vice versa, an IS

ratio of 0 indicates that none of the CRLs in an ARO have IS elements in proximity. Similarly, the Replicon ratio was calculated per ARO based on the CRLs' location on either plasmids or chromosomes. A replicon ratio of 1 means that all CRLs in a given ARO are of plasmid origin and a 0 means that all CRLs are from chromosomes. The Integron ratio indicates how many CRLs are inserted in integrons per ARO. For measuring the taxonomic distribution of each ARO category, the Simpson diversity index (range 0 to 1) was calculated per ARO using unclustered sequences and the genera they were identified in.

The ARG-MOB scale (0-1) represents the mean of the four MOB metrics described above (plasmid, IS, and integron association and Simpson diversity index) and serves as a ranking scheme to evaluate the degree to which members of an AROs have been mobilized. Based on the smoothed kernel density estimates of all ARG-MOB scores, groupings were made to categorize AROs by their ARG-MOB score. An ARG-MOB score of 0 indicates that ARGs of the given ARO were not once found to be mobilized in the RefSeq genomes and a score of 0 is thus categorized as *Very low*. Valleys in the density distribution of ARG-MOB scores were used to computationally pinpoint thresholds between ARG-MOB categories. The *Low* group ranges ARG-MOB score from 0.0 to 0.182, the *Medium* group ranges from 0.182 to 0.378, *High* ranges from 0.378 to 0.685, and *Very high* ranges from 0.685 to 1.0. For the *Low-Medium* and *Medium-High* cutoffs, the low point in valleys was used to define values but no apparent valley is present between *High* and *Very high*. Instead, a linear model was fitted to the right-side slope of the *High* peak and another fitted to the approximately linear data range starting at ARG-MOB score 0.7. The intersection between the two linear models (0.685) was used as the cutoff between the *High* and *Very high* groups (Supplementary Fig. 8).

Availability of Source Code and Requirements

Project name: ARG-MOB

Project home page: https://github.com/tueknielsen/ARG-MOB

Operating system: Linux—Ubuntu

Programming language: bash, R

Other requirements: For a full list of software and databases, please consult the script ARG_MOB_v0_5.sh at https://github.com/tueknielsen/ARG-MOB.

License: MIT license

Data Availability

A searchable table of all results is available online as Dataset S1 (Additional file 1). All supporting data and materials are available in the *GigaScience* GigaDB database [98].

Declarations

List of Abbreviations

ANT: Aminoglycoside nucleotidyltransferases

ARG: Antibiotic resistance gene

ARO: Antibiotic resistance ontology

CALIN: Cluster of *attC* sites lacking integron-integrases

CRL: Clustered resistance locus

ICE: Integrative conjugative element

IS: Insertion sequence

MGE: Mobile genetic element

MOB: Mobilization parameter

MWU: Mann-Whitney U

VIM: Verone integron-encoded metallo-β-lactamase

Consent for publication

Not applicable.

Competing interests

The authors declare that they have no competing interests.

Author contributions:

Conceptualization: TKN, LHH

Methodology: TKN, PDB, LHH

Investigation: TKN, PDB

Visualization: TKN

Supervision: LHH

Writing—original draft: TKN, PDB, LHH

Writing—review & editing: TKN, PDB, LHH

Acknowledgements

We would like to thank the reviewers for providing valuable comments.

Additional Files

Additional file 1: Dataset1.html. An interactive table summarizing the results presented in the study. This file can be opened in a web browser.

Supplementary Material

The file “ARGMOB_Supplementary Material.pdf” contains the following supplementary materials:

Supplementary Text 1. Additional details about filtering of DIAMOND blastp hits against CARD database.

Supplementary Fig. 1. Distribution of DIAMOND blastp hits plotted as % ID against the bitratio. The defined filters are shown.

Supplementary Fig. 2. Major bacterial orders of ARG blastp hits passing either of the defined filters. This plot shows the taxonomic distribution of passing hits on the Order level. Only orders that constitute >0.2% of the total hits are shown here.

Supplementary Fig. 3. Minor bacterial orders of ARG blastp hits passing either of the defined filters. This plot shows the taxonomic distribution of passing hits on the Order level. Only orders that constitute <0.2% of the total hits are shown here.

Supplementary Fig. 4. Violin plot of the lengths of unit- and composite transposons in The Transposon Registry. The mean length of transposons from this curated database is used to find the length cutoff for investigating the proximity of ARGs.

Supplementary Table 1. Summary of filters and cutoffs for including ARG and IS element blastp hits.

Supplementary Text 2. Investigation of the taxonomic biases and skews of the RefSeq complete genome database and the CARD database. These two databases are both biased, but they are not biased towards the same genera.

Supplementary Fig. 5. Biased composition of the RefSeq complete genome database investigated at Order level.

Supplementary Fig. 6. Comparison of the relative representation of genera in the RefSeq complete genome database and the CARD database. The databases are not skewed towards the same genera. The biggest differences are from the genera *Acinetobacter, Klebsiella, Escherichia, Salmonella, Streptococcus,* and *Bordetella.*

Supplementary Text 3. By clustering 176,688 genetic loci with ARGs to 53,895 Clustered Resistance Loci, we reduce the differential biases between the databases. Furthermore, we reduce the compositional database biases stemming from the presence of many almost identical genomes (e.g. *E. coli* genomes) in RefSeq.

Supplementary Fig. 7. Effect of clustering ARG loci to CRLs. Several of the most abundant genera in RefSeq are reduced in their relative abundance by clustering of almost identical ARG loci. Loci with antibiotic efflux ARGs are compressed more by clustering than other resistance mechanisms.

Supplementary Table 2. Overview of blastp hits passing filters, number of CRLs formed, and total number of AROs represented.

Supplementary Fig. 8. Smoothed kernel density estimates of all AROs and their ARG-MOB values. In this density plot, the cutoffs between ARG-MOB groupings (calculated by identification of local minima in density distribution) are shown. For the cutoff between *High* and *Very high* groups, no local minimum could be found. Instead, linear models were fitted in two approximately linear ARG-MOB ranges. The intersection of the two linear models were chosen as the cutoff.

Supplementary Text 4 and Supplementary Figure 9. 16S rRNA genes were identified in the studied complete genomes and IS elements were identified within the same proximity as for ARGs. This serves as a control, in that 16S rRNA is rarely expected to be associated with IS elements. Within 12.17 kbp on either side of 16S rRNA genes, IS elements were identified in 5.59% of cases. We consider this an indication of a false-positive rate of approx. 5% of association of ARGs with IS elements.

Supplementary Text 5. Investigation of which IS element families co-occur with individual resistance mechanisms.

Supplementary Fig. 10. Boxplots of abundance of IS families in proximity to ARGs, shown by major resistance mechanism. Significance values for Mann-Whitney tests are shown above boxplots.

Supplementary Text 6. The distance, in terms of bases, between IS elements and ARGs are discussed here. Efflux ARGs have significantly larger distance to IS elements than other mechanisms.

Supplementary Fig. 11. a: Density plots of IS ratio against distance to nearest IS element per ARO. b: boxplot of the ARG-IS distance per resistance mechanism with Mann-Whitney tests.

Supplementary Fig. 12. Replicon ratios of the 10 most abundant Proteobacteria families. ARO points are colored by their Replicon ratio and sized by how many unique CRLs belong to them.

Supplementary Fig. 13. IS ratios of Firmicutes families. ARO points are colored by their Replicon ratio and sized by how many unique CRLs belong to them. Many Firmicutes families are associated with the environment but some are associated with human activites, such as *Enterococcaceae* and *Staphylococcaceae*. This plot illustrates well the differences in ARG mobilization in environmental and human-associated bacteria.

Supplementary Table 3. Pairwise Mann-Whitney tests for IS and Replicon ratios in *Enterobacteriaceae* genera.

Supplementary Fig. 14. a: Overview of major mechanisms associated with integrons. b: submechanisms associated with integrons. ANT(3’’) is the most integron-associated submechanism.

Supplementary Table 4. Table of the top 20 AROs associated with integrons with their % integron association.

Supplementary Text 7 and Supplementary Figures 15-19. Pearson correlation coefficient analyses for major mechanisms. For each of the five major mechanisms, pairwise Pearson correlation coefficients are calculated and shown, similar to main figure 5a.

Supplementary Fig. 20. Bar charts of co-occurrence of IS elements, chromosome/plasmid location, and integrons per major mechanism. This shows that e.g. efflux ARGs are most often located on chromosomes and not in association with integrons or IS elements. On the other hand, inactivation ARGs are often found on plasmids and in association with IS elements and integrons.

Supplementary Text 8, Supplementary Figures 21-22, and Supplementary Table 5. Some AROs are highly divergent in mobilization. It is here discussed that some AROs have a large spread from their mean IS and Replicon ratios. As discussed in the main text, the *oqxAB* efflux pump genes are good examples of AROs with low IS and Replicon ratio means, since they are found un-mobilized on almost all *Klebsiella* genomes, but have been mobilized in many *Escherichia* and *Salmonella* strains, resulting in large spreads from the mean IS and Replicon ratios. It is also discussed that *Proteus*, *Pseudomonas*, *Morganella*, and *Acinetobacter* may act as reservoirs of yet unmobilized potential ARGs.

Supplementary Fig. 23. Count density of ARG-MOB per mechanism. Similar to main figure 6a, the count density of ARG-MOB categories are shown per major resistance mechanism.

Supplementary Figure 24. Antimicrobial compounds to which there are ARGs with *Very high* or *High* ARG-MOB scores.

Supplementary Text 9. Some examples Additional information and analyses for integron-ARG associations. Some examples of low and high ARG-MOB AROs are discussed with references. These examples illustrate that low ARG-MOB scoring AROs have generally only been shown to confer resistance in broad screenings where genes have been randomly cloned into strong expression vectors. On the other hand, high ARG-MOB scoring AROs were identified in clinical isolates that were experimentally verified to be resistant.

References

1. UN Interagency Coordination Group on Antimicrobial Resistance. NO TIME TO WAIT: SECURING THE FUTURE FROM DRUG-RESISTANT INFECTIONS. 2019. https://www.who.int/publications/i/item/no-time-to-wait-securing-the-future-from-drug-resistant-infections

2. Ebmeyer S, Kristiansson E, Larsson DGJ. A framework for identifying the recent origins of mobile antibiotic resistance genes. Communications Biology. 2021; doi: 10.1038/s42003-020-01545-5.

3. Mohammad R, Erik K, Carl-Fredrik F, Joakim LDG, M. LT. The Association between Insertion Sequences and Antibiotic Resistance Genes. mSphere. American Society for Microbiology; 2021; doi: 10.1128/mSphere.00418-20.

4. Allen HK, Donato J, Wang HH, Cloud-Hansen KA, Davies J, Handelsman J. Call of the wild: Antibiotic resistance genes in natural environments. Nature Reviews Microbiology. 2010; doi: 10.1038/nrmicro2312.

5. Martinez JL. The role of natural environments in the evolution of resistance traits in pathogenic bacteria. Proceedings of the Royal Society B: Biological Sciences. 2009; doi: 10.1098/rspb.2009.0320.

6. Yoon E-J, Goussard S, Touchon M, Krizova L, Cerqueira G, Murphy C, et al. Origin in Acinetobacter guillouiae and Dissemination of the Aminoglycoside-Modifying Enzyme Aph(3′)-VI. Davies JE, editor. mBio. 2014; doi: 10.1128/mBio.01972-14.

7. Alvarez-Ortega C, Olivares J, Martinez J. RND multidrug efflux pumps: what are they good for? Frontiers in Microbiology; doi: 10.3389/fmicb.2013.00007.

8. Clemente JC, Pehrsson EC, Blaser MJ, Sandhu K, Gao Z, Wang B, et al. The microbiome of uncontacted Amerindians. Science Advances. 2015; doi: 10.1126/sciadv.1500183.

9. Sommer MOA, Dantas G, Church GM. Functional characterization of the antibiotic resistance reservoir in the human microflora. Science. 2009; doi: 10.1126/science.1176950.

10. Blanco P, Hernando-Amado S, Reales-Calderon J, Corona F, Lira F, Alcalde-Rico M, et al. Bacterial Multidrug Efflux Pumps: Much More Than Antibiotic Resistance Determinants. Microorganisms. 2016; doi: 10.3390/microorganisms4010014.

11. Dantas G, Sommer MOA. Context matters - the complex interplay between resistome genotypes and resistance phenotypes. Current Opinion in Microbiology. 2012; doi: 10.1016/j.mib.2012.07.004.

12. Ito R, Pacey MP, Mettus RT, Sluis-Cremer N, Doi Y. Origin of the plasmid-mediated fosfomycin resistance gene fosA3. Journal of Antimicrobial Chemotherapy. 2018; doi: 10.1093/jac/dkx389.

13. Poirel L, Figueiredo S, Cattoir V, Carattoli A, Nordmann P. Acinetobacter radioresistens as a Silent Source of Carbapenem Resistance for Acinetobacter spp. Antimicrobial Agents and Chemotherapy. 2008; doi: 10.1128/AAC.01304-07.

14. Martínez JL, Coque TM, Baquero F. What is a resistance gene? Ranking risk in resistomes. Nature Reviews Microbiology. 2015; doi: 10.1038/nrmicro3399.

15. Kamruzzaman M, Patterson JD, Shoma S, Ginn AN, Partridge SR, Iredell JR. Relative strengths of promoters provided by common mobile genetic elements associated with resistance gene expression in Gram-negative bacteria. Antimicrobial Agents and Chemotherapy. 2015; doi: 10.1128/AAC.00420-15.

16. Rodríguez-Beltrán J, DelaFuente J, León-Sampedro R, MacLean RC, San Millán Á. Beyond horizontal gene transfer: the role of plasmids in bacterial evolution. Nature Reviews Microbiology. 2021; doi: 10.1038/s41579-020-00497-1.

17. Botelho J, Schulenburg H. The Role of Integrative and Conjugative Elements in Antibiotic Resistance Evolution. Trends in Microbiology. Elsevier; 2020; doi: 10.1016/j.tim.2020.05.011.

18. Partridge SR, Kwong SM, Firth N, Jensen SO. Mobile genetic elements associated with antimicrobial resistance. Clinical Microbiology Reviews. 2018; doi: 10.1128/CMR.00088-17.

19. Gillings MR. Integrons: Past, Present, and Future. Microbiology and Molecular Biology Reviews. 2014; doi: 10.1128/mmbr.00056-13.

20. Waglechner N, Wright GD. Antibiotic resistance: It's bad, but why isn't it worse? BMC Biology. 2017; doi: 10.1186/s12915-017-0423-1.

21. Dcosta VM, King CE, Kalan L, Morar M, Sung WWL, Schwarz C, et al. Antibiotic resistance is ancient. Nature. 2011; doi: 10.1038/nature10388.

22. Allen HK, Moe LA, Rodbumrer J, Gaarder A, Handelsman J. Functional metagenomics reveals diverse B-lactamases in a remote Alaskan soil. ISME Journal. 2009; doi: 10.1038/ismej.2008.86.

23. Martínez JL, Coque TM, Baquero F. What is a resistance gene? Ranking risk in resistomes. Nature Reviews Microbiology. 2015; doi: 10.1038/nrmicro3399.

24. Hernando-Amado S, Blanco P, Alcalde-Rico M, Corona F, Reales-Calderón JA, Sánchez MB, et al. Multidrug efflux pumps as main players in intrinsic and acquired resistance to antimicrobials. Drug Resistance Updates. 2016; doi: 10.1016/j.drup.2016.06.007.

25. Henderson TA, Young KD, Denome SA, Elf PK. AmpC and AmpH, proteins related to the class C β-lactamases, bind penicillin and contribute to the normal morphology of Escherichia coli. Journal of Bacteriology. 1997; doi: 10.1128/jb.179.19.6112-6121.1997.

26. Mickiewicz KM, Kawai Y, Drage L, Gomes MC, Davison F, Pickard R, et al. Possible role of L-form switching in recurrent urinary tract infection. Nature Communications. 2019; doi: 10.1038/s41467-019-12359-3.

27. Khedher M Ben, Baron SA, Riziki T, Ruimy R, Raoult D, Diene SM, et al. Massive analysis of 64,628 bacterial genomes to decipher water reservoir and origin of mobile colistin resistance genes: is there another role for these enzymes? Scientific Reports. 2020; doi: 10.1038/s41598-020-63167-5.

28. Bengtsson-Palme J, Larsson DGJ. Antibiotic resistance genes in the environment: prioritizing risks. Nature Reviews Microbiology. 2015; doi: 10.1038/nrmicro3399-c1.

29. Bengtsson-Palme J, Angelin M, Huss M, Kjellqvist S, Kristiansson E, Palmgren H, et al. The human gut microbiome as a transporter of antibiotic resistance genes between continents. Antimicrobial Agents and Chemotherapy. 2015; doi: 10.1128/AAC.00933-15.

30. Hughes D, Andersson DI. Environmental and genetic modulation of the phenotypic expression of antibiotic resistance. FEMS Microbiology Reviews. 2017; doi: 10.1093/femsre/fux004.

31. Moran RA, Anantham S, Holt KE, Hall RM. Prediction of antibiotic resistance from antibiotic resistance genes detected in antibiotic-resistant commensal Escherichia coli using PCR or WGS. Journal of Antimicrobial Chemotherapy. 2017; doi: 10.1093/jac/dkw511.

32. Thomas M, Fenske GJ, Antony L, Ghimire S, Welsh R, Ramachandran A, et al. Whole genome sequencing-based detection of antimicrobial resistance and virulence in non-typhoidal Salmonella enterica isolated from wildlife. Gut Pathogens. 2017; doi: 10.1186/s13099-017-0213-x.

33. Kos VN, Déraspe M, McLaughlin RE, Whiteaker JD, Roy PH, Alm RA, et al. The resistome of Pseudomonas aeruginosa in relationship to phenotypic susceptibility. Antimicrobial Agents and Chemotherapy. 2015; doi: 10.1128/AAC.03954-14.

34. Mahfouz N, Ferreira I, Beisken S, von Haeseler A, Posch AE. Large-scale assessment of antimicrobial resistance marker databases for genetic phenotype prediction: a systematic review. Journal of Antimicrobial Chemotherapy. 2020; doi: 10.1093/jac/dkaa257.

35. Ellington MJ, Ekelund O, Aarestrup FM, Canton R, Doumith M, Giske C, et al. The role of whole genome sequencing in antimicrobial susceptibility testing of bacteria: report from the EUCAST Subcommittee. Clinical Microbiology and Infection. Elsevier; 2017; doi: 10.1016/j.cmi.2016.11.012.

36. Chen ML, Doddi A, Royer J, Freschi L, Schito M, Ezewudo M, et al. Beyond multidrug resistance: Leveraging rare variants with machine and statistical learning models in Mycobacterium tuberculosis resistance prediction. EBioMedicine. Elsevier; 2019; doi: 10.1016/j.ebiom.2019.04.016.

37. Marcus N, Wesley LS, F. MP, J. OR, Robert O, L. SR, et al. Using Machine Learning To Predict Antimicrobial MICs and Associated Genomic Features for Nontyphoidal Salmonella. Journal of Clinical Microbiology. American Society for Microbiology; 2021; doi: 10.1128/JCM.01260-18.

38. Moradigaravand D, Palm M, Farewell A, Mustonen V, Warringer J, Parts L. Prediction of antibiotic resistance in Escherichia coli from large-scale pan-genome data. PLOS Computational Biology. doi: 10.1371/journal.pcbi.1006258.

39. Vanessa K, Ayush K. Update on Multidrug Resistance Efflux Pumps in Acinetobacter spp. Antimicrobial Agents and Chemotherapy. American Society for Microbiology; 2021; doi: 10.1128/AAC.00514-21.

40. Alcalde-Rico M, Hernando-Amado S, Blanco P, Martínez JL. Multidrug Efflux Pumps at the Crossroad between Antibiotic Resistance and Bacterial Virulence. Frontiers in Microbiology. doi: 10.3389/fmicb.2016.01483.

41. Du D, Wang-Kan X, Neuberger A, van Veen HW, Pos KM, Piddock LJ V, et al. Multidrug efflux pumps: structure, function and regulation. Nature Reviews Microbiology. 2018; doi: 10.1038/s41579-018-0048-6.

42. Romero D, Traxler MF, López D, Kolter R. Antibiotics as signal molecules. Chemical Reviews. 2011; doi: 10.1021/cr2000509.

43. Forsberg KJ, Patel S, Gibson MK, Lauber CL, Knight R, Fierer N, et al. Bacterial phylogeny structures soil resistomes across habitats. Nature. 2014; doi: 10.1038/nature13377.

44. dos Santos DFK, Istvan P, Quirino BF, Kruger RH. Functional Metagenomics as a Tool for Identification of New Antibiotic Resistance Genes from Natural Environments. Microbial Ecology. 2017; doi: 10.1007/s00248-016-0866-x.

45. McCoy AJ, Sandlin RC, Maurelli AT. In vitro and in vivo functional activity of Chlamydia MurA, a UDP-N-acetylglucosamine enolpyruvyl transferase involved in peptidoglycan synthesis and fosfomycin resistance. Journal of Bacteriology. 2003; doi: 10.1128/JB.185.4.1218-1228.2003.

46. Truong-Bolduc QC, Dunman PM, Strahilevitz J, Projan SJ, Hooper DC. MgrA is a multiple regulator of two new efflux pumps in Staphylococcus aureus. Journal of Bacteriology. 2005; doi: 10.1128/JB.187.7.2395-2405.2005.

47. Cundliffe E. Glycosylation of macrolide antibiotics in extracts of Streptomyces lividans. Antimicrobial Agents and Chemotherapy. 1992; doi: 10.1128/AAC.36.2.348.

48. Rossolini GM, Franceschini N, Lauretti L, Caravelli B, Riccio ML, Galleni M, et al. Cloning of a Chryseobacterium (Flavobacterium) meningosepticum chromosomal gene (blaA(CME)) encoding an extended-spectrum class a β- lactamase related to the Bacteroides cephalosporinases and the

VEB-1 and PER β-lactamases. Antimicrobial Agents and Chemotherapy. 1999; doi: 10.1128/aac.43.9.2193.

49. Hegde SS, Vetting MW, Roderick SL, Mitchenall LA, Maxwell A, Takiff HE, et al. Biochemistry: A fluoroquinolone resistance protein from Mycobacterium tuberculosis that mimics DNA. Science (1979). 2005; doi: 10.1126/science.1110699.

50. Okazaki A, Avison MB. Aph(3′)-IIc, an aminoglycoside resistance determinant from Stenotrophomonas maltophilia. Antimicrobial Agents and Chemotherapy. 2007; doi: 10.1128/AAC.00795-06.

51. Singleton CM, Petriglieri F, Kristensen JM, Kirkegaard RH, Michaelsen TY, Andersen MH, et al. Connecting structure to function with the recovery of over 1000 high-quality metagenome-assembled genomes from activated sludge using long-read sequencing. Nat Commun. 2021; doi: 10.1038/s41467-021-22203-2.

52. Larsson DGJ, Andremont A, Bengtsson-Palme J, Brandt KK, de Roda Husman AM, Fagerstedt P, et al. Critical knowledge gaps and research needs related to the environmental dimensions of antibiotic resistance. Environment International. 2018; doi: 10.1016/j.envint.2018.04.041.

53. MacLean RC, San Millan A. The evolution of antibiotic resistance. Science. 2019; doi: 10.1126/science.aax3879.

54. Gillings MR, Paulsen IT, Tetu SG. Genomics and the evolution of antibiotic resistance. Ann N Y Acad Sci. 2017; doi: 10.1111/nyas.13268.

55. Siguier P, Gourbeyre E, Chandler M. Bacterial insertion sequences: Their genomic impact and diversity. FEMS Microbiology Reviews. 2014; doi: 10.1111/1574-6976.12067.

56. Mahillon J, Chandler M. Insertion sequences. Microbiol Mol Biol Rev. American Society for Microbiology; doi: 10.1128/MMBR.62.3.725-774.1998.

57. Berendonk TU, Manaia CM, Merlin C, Fatta-Kassinos D, Cytryn E, Walsh F, et al. Tackling antibiotic resistance: The environmental framework. Nature Reviews Microbiology. 2015; doi: 10.1038/nrmicro3439.

58. Sommer MOA, Munck C, Toft-Kehler RV, Andersson DI. Prediction of antibiotic resistance: Time for a new preclinical paradigm? Nature Reviews Microbiology. 2017; doi: 10.1038/nrmicro.2017.75.

59. Bengtsson-Palme J. The diversity of uncharacterized antibiotic resistance genes can be predicted from known gene variants-but not always. Microbiome. 2018; doi: 10.1186/s40168-018-0508-2.

60. Enault F, Briet A, Bouteille L, Roux S, Sullivan MB, Petit MA. Phages rarely encode antibiotic resistance genes: A cautionary tale for virome analyses. ISME Journal. 2017; doi: 10.1038/ismej.2016.90.

61. Dion MB, Oechslin F, Moineau S. Phage diversity, genomics and phylogeny. Nature Reviews Microbiology. 2020; doi: 10.1038/s41579-019-0311-5.

62. Tansirichaiya S, Rahman MA, Roberts AP. The Transposon Registry. Mob DNA. 2019; doi: 10.1186/s13100-019-0182-3.

63. Cury J, Jové T, Touchon M, Néron B, Rocha EP. Identification and analysis of integrons and cassette arrays in bacterial genomes. Nucleic Acids Research. 2016; doi: 10.1093/nar/gkw319.

64. Ramirez MS, Tolmasky ME. Aminoglycoside modifying enzymes. Drug Resistance Updates. 2010; doi: 10.1016/j.drup.2010.08.003.

65. Norman A, Hansen LH, She Q, Sørensen SJ. Nucleotide sequence of pOLA52: A conjugative IncX1 plasmid from Escherichia coli which enables biofilm formation and multidrug efflux. Plasmid. 2008; doi: 10.1016/j.plasmid.2008.03.003.

66. Hansen LH, Johannesen E, Burmølle M, Sørensen AH, Sørensen SJ. Plasmid-encoded multidrug efflux pump conferring resistance to olaquindox in Escherichia coli. Antimicrobial Agents and Chemotherapy. 2004; doi: 10.1128/AAC.48.9.3332-3337.2004.

67. Li J, Zhang H, Ning J, Sajid A, Cheng G, Yuan Z, et al. The nature and epidemiology of OqxAB, a multidrug efflux pump. Antimicrobial Resistance and Infection Control. 2019; doi: 10.1186/s13756-019-0489-3.

68. Perez F, Rudin SD, Marshall SH, Coakley P, Chen L, Kreiswirth BN, et al. OqxAB, a quinolone and olaquindox efflux pump, is widely distributed among multidrug-resistant Klebsiella pneumoniae isolates of human origin. Antimicrobial Agents and Chemotherapy. 2013; doi: 10.1128/AAC.00725-13.

69. Yuan J, Xu X, Guo Q, Zhao X, Ye X, Guo Y, et al. Prevalence of the oqxAB gene complex in Klebsiella pneumoniae and Escherichia coli clinical isolates. Journal of Antimicrobial Chemotherapy. 2012; doi: 10.1093/jac/dks086.

70. Miriagou V, Tzelepi E, Gianneli D, Tzouvelekis LS. Escherichia coli with a self-transferable, multiresistant plasmid coding for metallo-β-lactamase VIM-1. Antimicrobial Agents and Chemotherapy. 2003; doi: 10.1128/AAC.47.1.395-397.2003.

71. Bush K, Bradford PA. Epidemiology of β-Lactamase-Producing Pathogens. Clinical Microbiology Reviews. 2020; doi: 10.1128/CMR.00047-19.

72. Ceccarelli D, Bani S, Cappuccinelli P, Colombo MM. Prevalence of aadA1 and dfrA15 class 1 integron cassettes and SXT circulation in Vibrio cholerae O1 isolates from Africa. Journal of Antimicrobial Chemotherapy. 2006; doi: 10.1093/jac/dkl352.

73. Berglund F, Österlund T, Boulund F, Marathe NP, Larsson DGJ, Kristiansson E. Identification and reconstruction of novel antibiotic resistance genes from metagenomes. Microbiome. 2019; doi: 10.1186/s40168-019-0670-1.

74. Guillard T, Lebreil AL, Hansen LH, Kisserli A, Berger S, Lozniewski A, et al. Discrimination between native and Tn6010-associated oqxAB in Klebsiella spp., Raoultella spp., and other enterobacteriaceae by using a two-step strategy. Antimicrobial Agents and Chemotherapy. 2015; doi: 10.1128/AAC.00669-15.

75. Bunny KL, Hall RM, Stokes HW. New mobile gene cassettes containing an aminoglycoside resistance gene, aacA7, and a chloramphenicol resistance gene, catB3, in an integron in pBWH301. Antimicrobial Agents and Chemotherapy. 1995; doi: 10.1128/AAC.39.3.686.

76. Tribuddharat C, Fennewald M. Integron-mediated rifampin resistance in Pseudomonas aeruginosa. Antimicrobial Agents and Chemotherapy. 1999; doi: 10.1128/aac.43.4.960.

77. Browne AJ, Chipeta MG, Haines-Woodhouse G, Kumaran EPA, Hamadani BHK, Zaraa S, et al. Global antibiotic consumption and usage in humans, 2000–18: a spatial modelling study. The Lancet Planetary Health. 2021; doi: https://doi.org/10.1016/S2542-5196(21)00280-1.

78. Kai Blin: ncbi-genome-download 0.2.11. https://github.com/kblin/ncbi-genome-download Accessed 2022 Jun 21.

79. Hyatt D, Chen GL, LoCascio PF, Land ML, Larimer FW, Hauser LJ. Prodigal: Prokaryotic gene recognition and translation initiation site identification. BMC Bioinformatics. 2010; doi: 10.1186/1471-2105-11-119.

80. McArthur AG, Waglechner N, Nizam F, Yan A, Azad MA, Baylay AJ, et al. The comprehensive antibiotic resistance database. Antimicrobial Agents and Chemotherapy. 2013; doi: 10.1128/AAC.00419-13.

81. Liu B, Pop M. ARDB - Antibiotic resistance genes database. Nucleic Acids Research. 2009; doi: 10.1093/nar/gkn656.

82. Lakin SM, Dean C, Noyes NR, Dettenwanger A, Ross AS, Doster E, et al. MEGARes: An antimicrobial resistance database for high throughput sequencing. Nucleic Acids Research. 2017; doi: 10.1093/nar/gkw1009.

83. Zankari E, Hasman H, Cosentino S, Vestergaard M, Rasmussen S, Lund O, et al. Identification of acquired antimicrobial resistance genes. Journal of Antimicrobial Chemotherapy. 2012; doi: 10.1093/jac/dks261.

84. Yin X, Jiang XT, Chai B, Li L, Yang Y, Cole JR, et al. ARGs-OAP v2.0 with an expanded SARG database and Hidden Markov Models for enhancement characterization and quantification of antibiotic resistance genes in environmental metagenomes. Bioinformatics. 2018; doi: 10.1093/bioinformatics/bty053.

85. Gupta SK, Padmanabhan BR, Diene SM, Lopez-Rojas R, Kempf M, Landraud L, et al. ARG-annot, a new bioinformatic tool to discover antibiotic resistance genes in bacterial genomes. Antimicrobial Agents and Chemotherapy. 2014; doi: 10.1128/AAC.01310-13.

86. Arango-Argoty G, Garner E, Pruden A, Heath LS, Vikesland P, Zhang L. DeepARG: A deep learning approach for predicting antibiotic resistance genes from metagenomic data. Microbiome. 2018; doi: 10.1186/s40168-018-0401-z.

87. Arango-Argoty GA, Guron GKP, Guron GKP, Garner E, Riquelme M V., Heath LS, et al. ARGminer: A web platform for the crowdsourcing-based curation of antibiotic resistance genes. Bioinformatics. 2020; doi: 10.1093/bioinformatics/btaa095.

88. Wallace JC, Port JA, Smith MN, Faustman EM. FARME DB: A functional antibiotic resistance element database. Database. 2017; doi: 10.1093/database/baw165.

89. Buchfink B, Xie C, Huson DH. Fast and sensitive protein alignment using DIAMOND. Nature Methods. 2014; doi: 10.1038/nmeth.3176.

90. Alcock BP, Raphenya AR, Lau TTY, Tsang KK, Bouchard M, Edalatmand A, et al. CARD 2020: Antibiotic resistome surveillance with the comprehensive antibiotic resistance database. Nucleic Acids Research. 2020; doi: 10.1093/nar/gkz935.

91. Li H, Handsaker B, Wysoker A, Fennell T, Ruan J, Homer N, et al. The Sequence Alignment/Map format and SAMtools. Bioinformatics. 2009; doi: 10.1093/bioinformatics/btp352.

92. Danecek P, Bonfield JK, Liddle J, Marshall J, Ohan V, Pollard MO, et al.. Twelve years of SAMtools and BCFtools. Gigascience. 2021; doi: 10.1093/gigascience/giab008.

93. Browne PD, Nielsen TK, Kot W, Aggerholm A, Gilbert MTP, Puetz L, et al. GC bias affects genomic and metagenomic reconstructions, underrepresenting GC-poor organisms. Gigascience. 2020; doi: 10.1093/gigascience/giaa008.

94. Siguier P. ISfinder: the reference centre for bacterial insertion sequences. Nucleic Acids Research. 2006; doi: 10.1093/nar/gkj014.

95. Seemann T. Prokka: Rapid prokaryotic genome annotation. Bioinformatics. 2014; doi: 10.1093/bioinformatics/btu153.

96. Seemann T. barrnap 0.9 : rapid ribosomal RNA prediction. https://github.com/tseemann/barrnap.

97. Edgar RC. Search and clustering orders of magnitude faster than BLAST. Bioinformatics. 2010; doi: 10.1093/bioinformatics/btq461.

98. Nielsen TK, Browne PD, Hansen LH, 2022. Supporting data for “Antibiotic resistance genes are differentially mobilized according to resistance mechanism.” GigaScience Database. http://dx.doi.org/10.5524/102232.

Click here to access/download;Figure;Fig1.eps

Figure 1

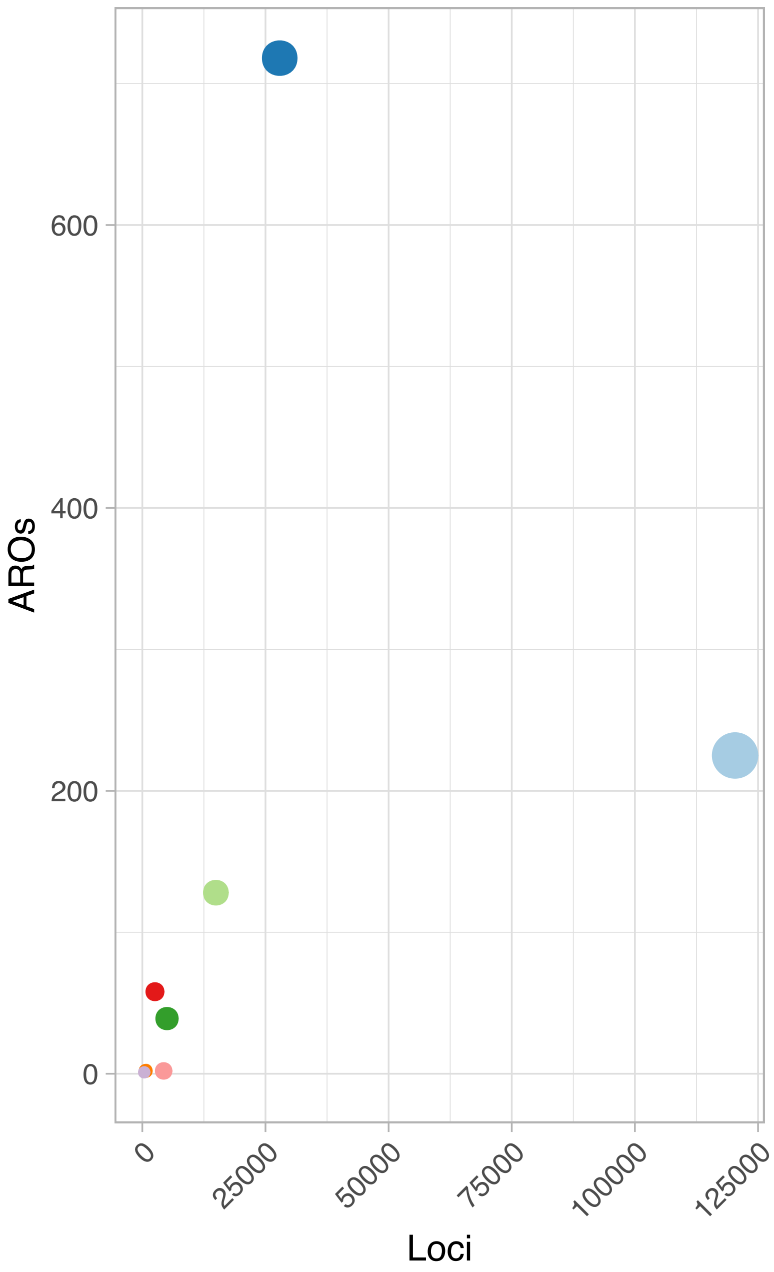

Mechanism	Loci	CRLs	AROs
Antibiotic efflux (AE)	120336	28953	225
Antibiotic inactivation (AI)	27889	13763	718
Antibiotic target alteration (ATA)	14931	4856	128
Antibiotic target replacement (ATR)	5009	3360	39
Antibiotic efflux (AE);<br>Reduced permeability to antibiotic (RPA)	4330	962	2
Antibiotic target protection (ATP)	2559	1428	58
Reduced permeability to antibiotic (RPA)	778	142	3
Antibiotic target alteration (ATA);<br>Antibiotic target replacement (ATR)	675	276	2
Antibiotic target alteration (ATA);<br>Antibiotic efflux (AE)	381	155	1

Figure 2

Click here to access/download;Figure;Fig2.eps

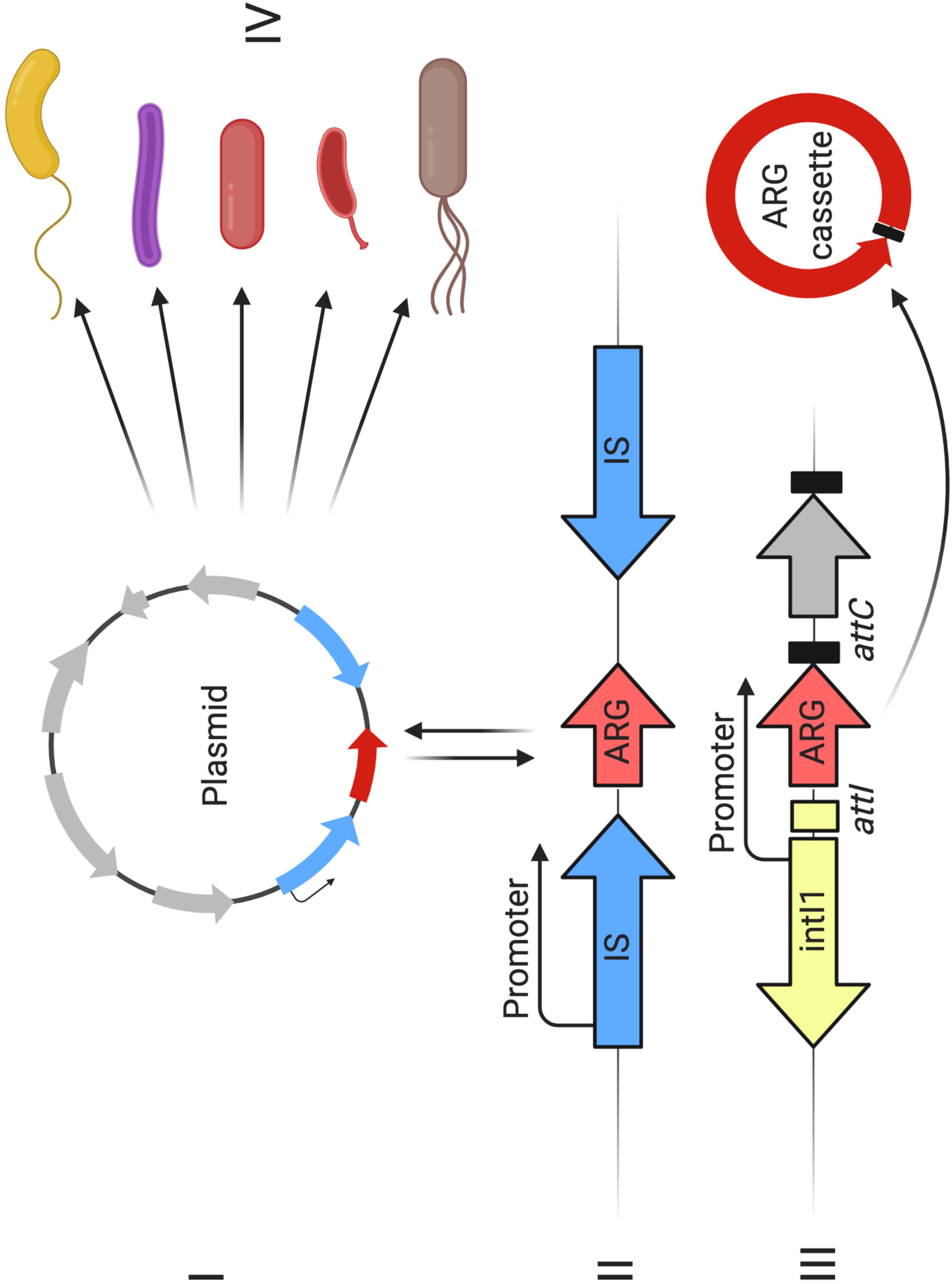

Figure 3

Click here to access/download;Figure;Fig3.eps

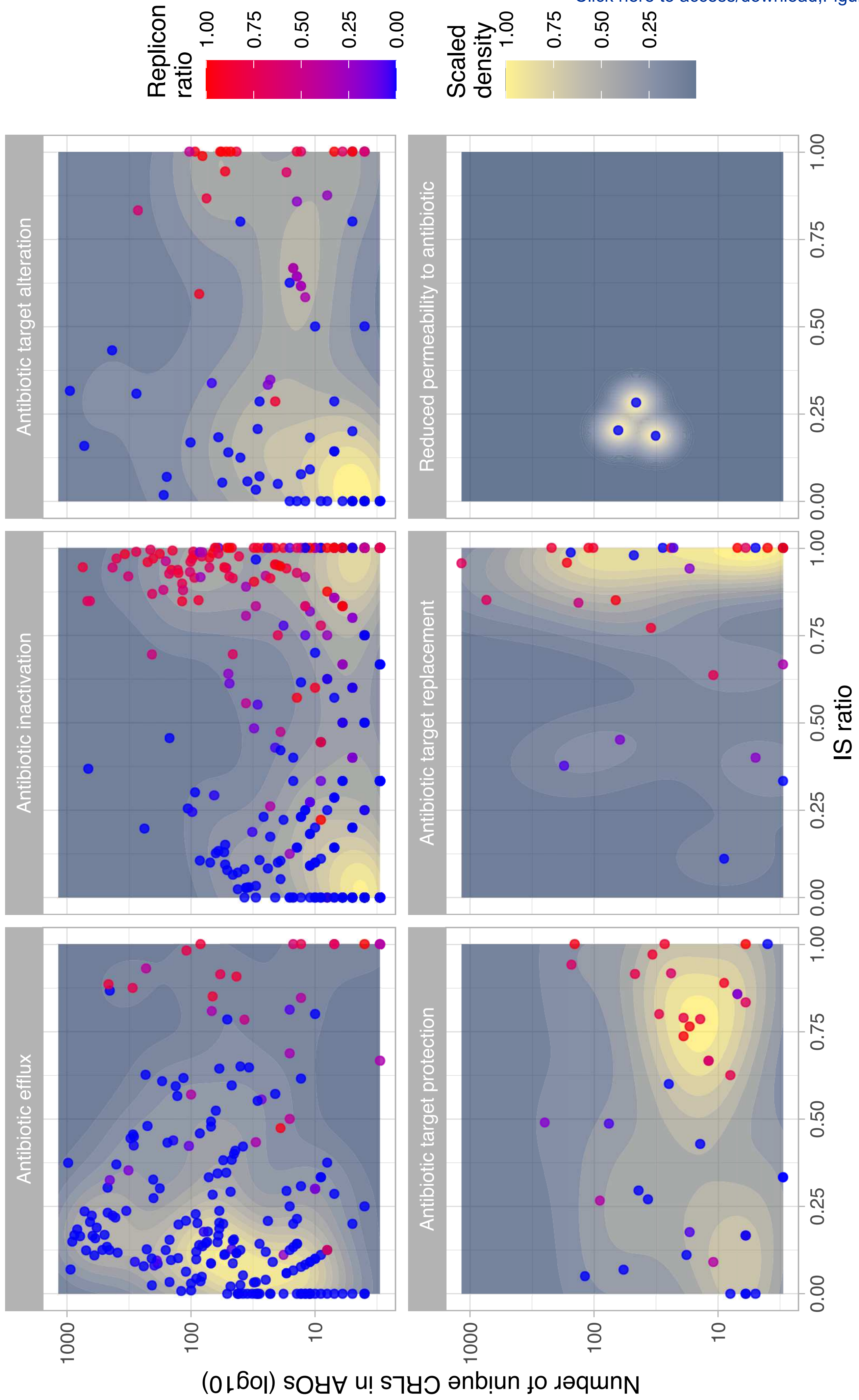

Figure 4

Click here to access/download;Figure;Fig4.eps

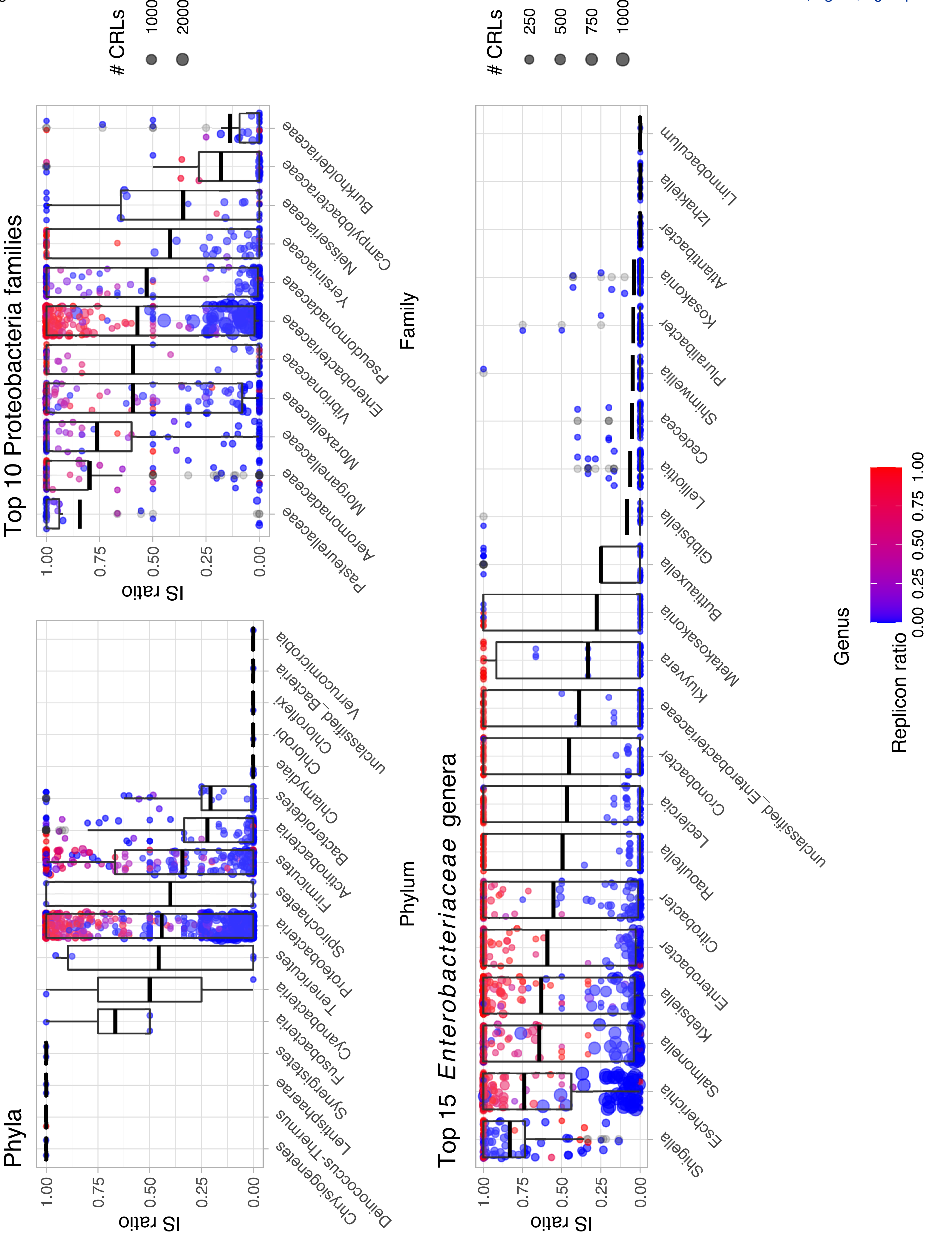

Figure 5

Click here to access/download;Figure;Fig5_revised.pdf

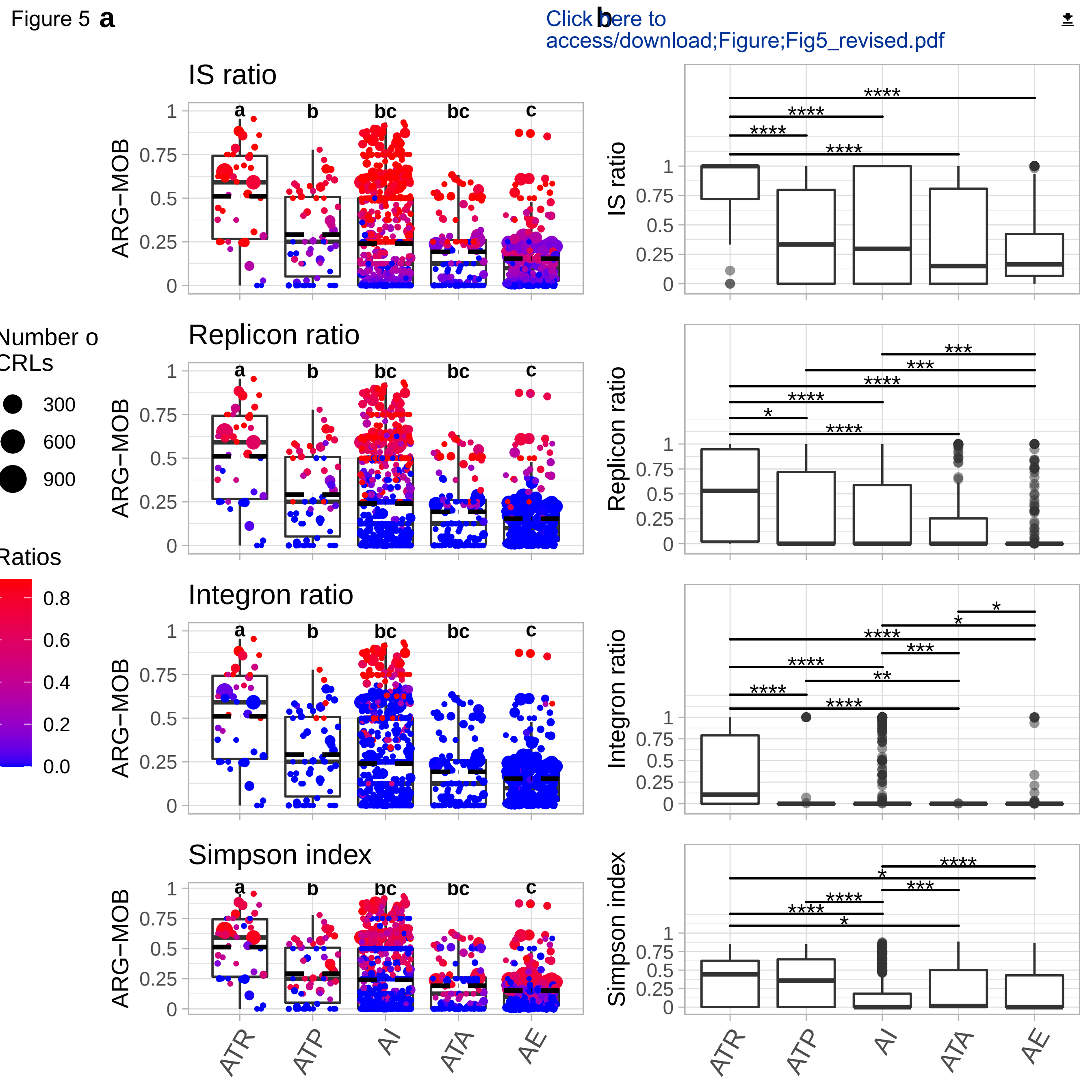

Figure 6 Click here to access/download;Figure;Fig6.eps

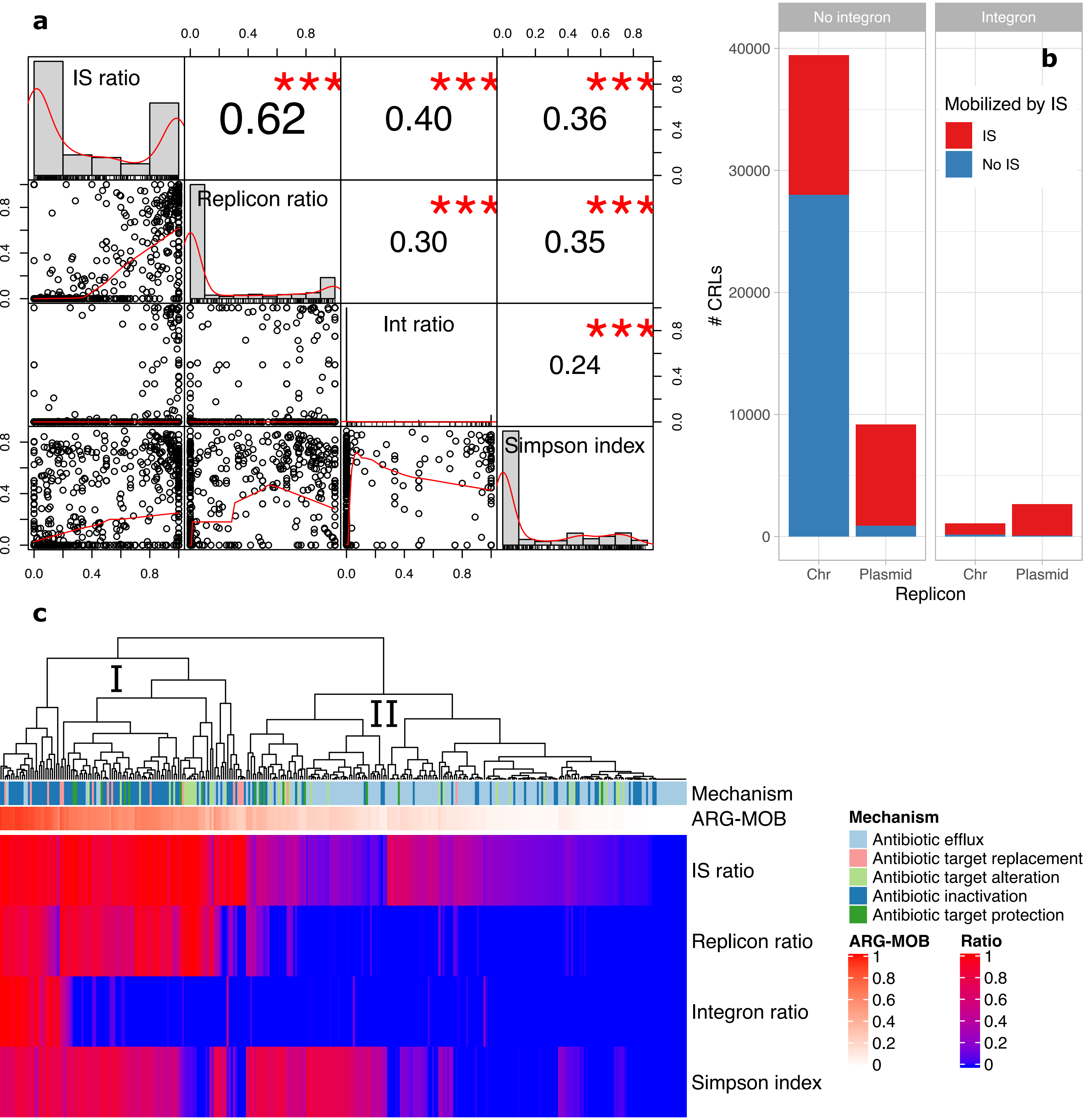

Figure 7

Click here to access/download;Figure;Fig7.pdf

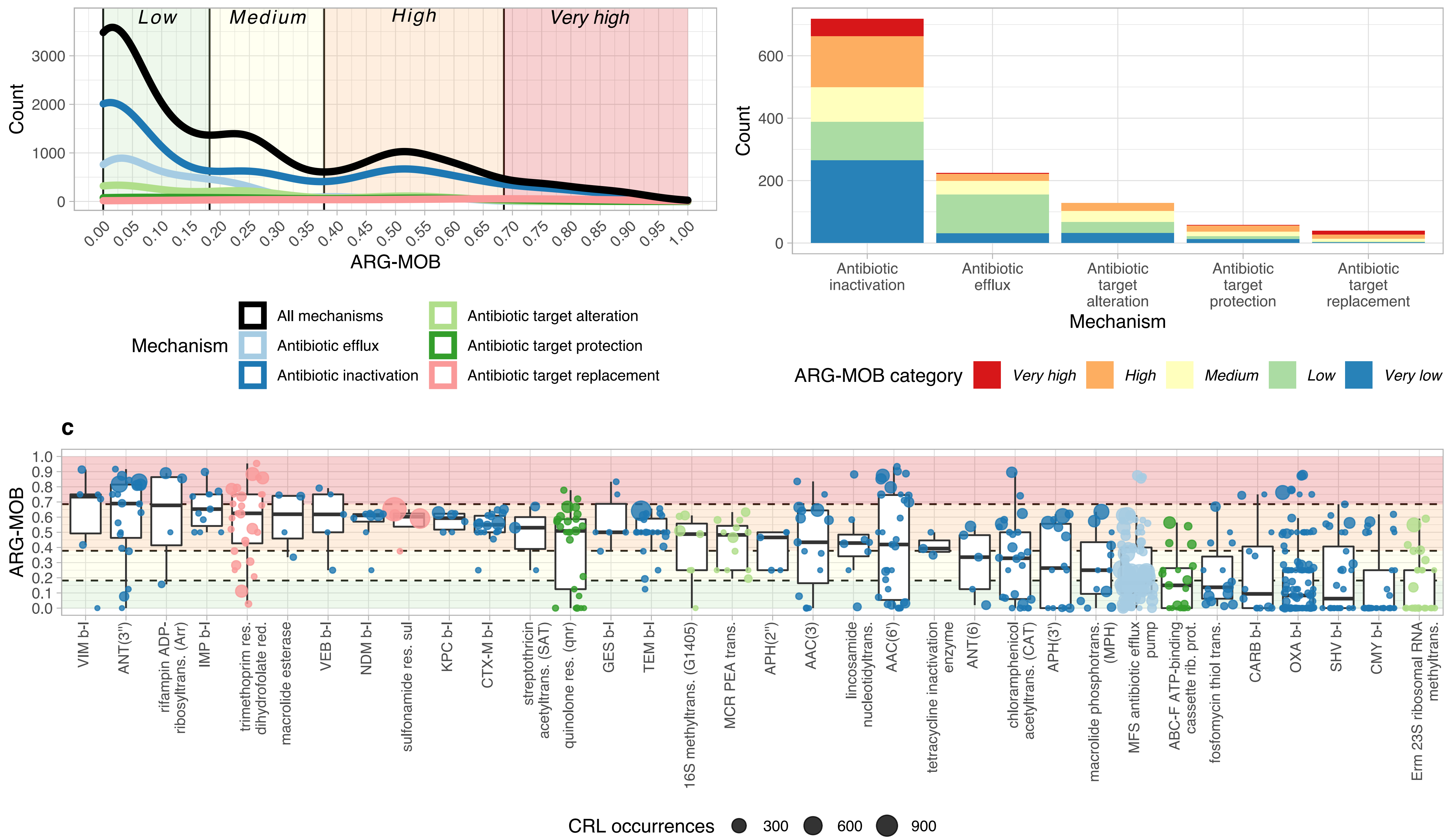

Click here to access/download
Supplementary Material
ARGMOB_Supp_GigaS_revised.pdf

Supplementary Data

Click here to access/download
Supplementary Material
Dataset1.html

Click here to access/download
Supplementary Material
Response_to_reviewers.pdf

Cover letter Click here to access/download;Personal Cover;Cover_letter.pdf

Cover letter for resubmission of GIGA-D-21-00206

Dear Editor

This submission is a resubmission of manuscript GIGA-D-21-00206, which received an "open reject" decision by editor Hongling Zhou.

We greatly appreciate the decision to change the 'Rejection' to 'Open Rejection' upon further considerations. We originally received comments from two non-anonymous reviewers that were very positive about our paper and from one anonymous reviewer that were very critical of both our approach and discussion. For several reasons, as is apparent in our attached response to reviewers, we do not feel that Reviewer #3 gave a fair and unbiased review.

Based on the very critical Reviewer #3, the original decision by the Editor was:
"I find myself largely agreeing with the critical reviewer here. Its really a shame he/she won't release their review. The distance cut off does seem arbitrary, and the lack of a negative control is really a substantial problem. They also seem to suggest similar recent papers which have not been cited , but I have not dug into that."

We have addressed all editor and reviewer concerns in our response and have thoroughly revised the manuscript, based on the feedback. The study is very much improved by this.

To address the editor concerns: Briefly, our distance cutoff of 12.17 kbp is actually not arbitrary, but based on empirical data from the trusted and curated The Transposon Registry database. Our empirical approach can be compared to the truly arbitrary cutoffs used in papers in the Razavi and Ebmeyer papers, that Reviewer #3 refers to. We believe that our empirical approach is much stronger and more sensible than the arbitrary cutoffs presented in these papers. We have furthermore addressed the controls comment, although we already had the 16S rRNA control, which Reviewer #3 apparently missed upon reading the manuscript. Finally, we have now included reference to the Razavi paper and others that each of the reviewers kindly suggested. The Ebmeyer paper was already referenced in the submitted version. We appreciate all the comments and suggestions made by the three reviewers and the manuscript is much stronger because of this.

Regarding the comments made by Reviewer #3:

While the critical and anonymous reviewer #3 clearly is an expert in the field (without being able to look at their publication record), we do not believe that he/she has given a fair review. Considering the very positive feedback and helpful comments from non-anonymous reviewers #1 and #2 (with long publication record within the field of ARGs), it is suspicious that reviewer #3 is so critical towards the manuscript with some fair points but others that are not justified.
Reviewer #3 often reference the Razavi et al. 2020 paper or papers coming from the same group of Joakim D.G. Larsson. We believe that the anonymous reviewer is one of the authors on the Razavi paper (Mohammad Razavi, Erik Kristiansson, Carl-Fredrik Flach, D. G. Joakim Larsson) or another person from dr. Larsson's group, possibly also dr. Larsson's long-time collaborator Johan Bengtsson-Palme or a colleague of his. While our manuscript has many similarities to the Razavi paper, there are many fundamental differences. We believe that reviewer #3 is a person that is influenced by a competitive angle to not see our manuscript published, since it on some points contradicts some of their work and on other points competes with their efforts to describe the association between ARGs and mobile genetic elements.
Our conclusion is that ARGs are overpredicted, since studies very rarely consider the genetic context that is so important for gene expression and thus phenotypic resistance. Many researchers, including dr. Larsson and colleagues, have produced impactful papers that describe the presence of ARGs in all sorts of environments, without considering the fact that the predicted ARGs likely do not confer resistance in most (environmental) cases. This opinion is somewhat controversial, as researchers have neglected this aspect for decades, but it is time to improve how we do resistance prediction. We believe that our paper strongly contributes to this endeavour and to the discussion on antibiotic resistance in general.
Within the ARG community, there has been a small divide where one side for long have been uncritically predicting ARGs in all environments, including pristine and ancient samples, and have consequently concluded that "ARGs are everywhere" and that resistance predates the use of antibiotics. Dr. Larsson and Dr. Bengtsson-Palme are leading scientists within this field. On the other side, which we subscribe to, Dr. José L. Martínez and Dr. Fernando Baquero have been focusing on the fact that ARGs perform non-resistance functions in natural genetic context and screening for so-called ARGs everywhere will lead to overestimation of resistance in natural settings, since e.g. all bacteria have efflux pumps but not all bacteria are resistant. This was discussed in their seminal paper "What is a resistance gene? Ranking risk in resistomes" in 2015 which was countered by a comment paper by Johan Bengtsson-Palme and D.G. Joakim Larson "Antibiotic resistance genes in the environment: prioritizing risks". In this comment paper, they advocate that Martinez and Baquero "*underappreciates the potential consequences of the transfer of previously unknown resistance determinants from the environmental resistome."*. From our manuscript, it is quite clear that we subscribe to the Martinez and Baquero concept of ARGs performing non-resistance functions until they are highly overexpressed (e.g. by mobilization). From the critical comments made by reviewer #3 it is likewise clear that he/she does not agree with this opinion and rather adheres to the opinion of Bengtsson-Palme and Larsson and consequently have not provided an unbiased, fair review. We acknowledge that there is an

ongoing debate between proponents of the two viewpoints and have accordingly softened the manuscript throughout, in order to not alienate readers on either side.

We do accommodate many of the points made in their review and are welcoming the suggestions that leads to an improved manuscript. However, as is documented in our “Response to reviewers”, some comments made by Reviewer #3 are objectively wrong. This includes a suggestion to change to Bonferroni correction, that the reviewer claims to not require independence in testing, which is simply not true. Likewise, reviewer #3 claims that our filters for ARG searches are too relaxed but instead refers to papers by Razavi and Ebmeyer and claims that they use better filtering. When going through their manuscript, it is apparent that they use more relaxed filters than we do, which makes us suspicious that reviewer #3 is either not giving a fair review or has failed to understand our manuscript, as well as the studies that he/she refers to. As much as possible, we have taken the opportunity to strengthen our analyses and manuscript based on the comments made by all reviewers. For more on why we believe that Reviewer #3 gave an unfair review, please see our rebuttals in the “Response to reviewers”.

We greatly appreciate the chance to submit our manuscript again, as we believe that GigaScience is a very relevant journal for our study.

Kind regards,
Tue Kjærgaard Nielsen
Assistant Professor
University of Copenhagen
Denmark
