# Supplementary material for: Antibiotic resistance genes are differentially mobilized according to resistance mechanism
Source: Gigascience. 2022 Jul 30;11:giac072. doi: 10.1093/gigascience/giac072 (PMC9338424; doi:10.1093/gigascience/giac072)
Supplement: giac072_Supplemental_Files [file giac072_supplemental_files.zip › ARGMOB_Supp_GigaS_revised.pdf]

Supplementary Materials for

Mobilization of antibiotic resistance genes differ by resistance mechanism

Tue Kjærgaard Nielsen*, Patrick Denis Browne, Lars Hestbjerg Hansen*

Corresponding authors: Tue Kjærgaard Nielsen, Lars Hestbjerg Hansen
Email: tkn@plen.ku.dk, lhha@plen.ku.dk

This PDF file includes:

Supplementary Text 1 to 9
Supplementary Figures 1 to 24
Supplementary Tables 1 to 5

Supplementary Text 1: Filtering DIAMOND blastp CARD hits

By default, all blastp hits with bitscores exceeding the per-ARG-curated RGI bitscore-cutoffs are accepted. A ratio (bitratio) is calculated by dividing bitscores with the RGI cutoffs where a bitratio of less than 1 indicates that the blastp hit has a lower bitscore than the RGI ARG cutoff. However, as can be seen in Supplementary Fig. 1, there are many blastp hits that have high percentage identity and high query coverage, although their bitscores are below 1 and would thus be discarded if only RGI cutoffs are considered. Considering the database biases described in the article and in further detail below, it is likely that these high-similarity but low-bitratio hits are actually true ARG homologs in strains that are not related to those highly abundant in the CARD database. To include these hits, another filter was introduced where hits with bitratio lower than 1 are still included if their % identity and % query coverage are above 80% (Supplementary Fig. 1).

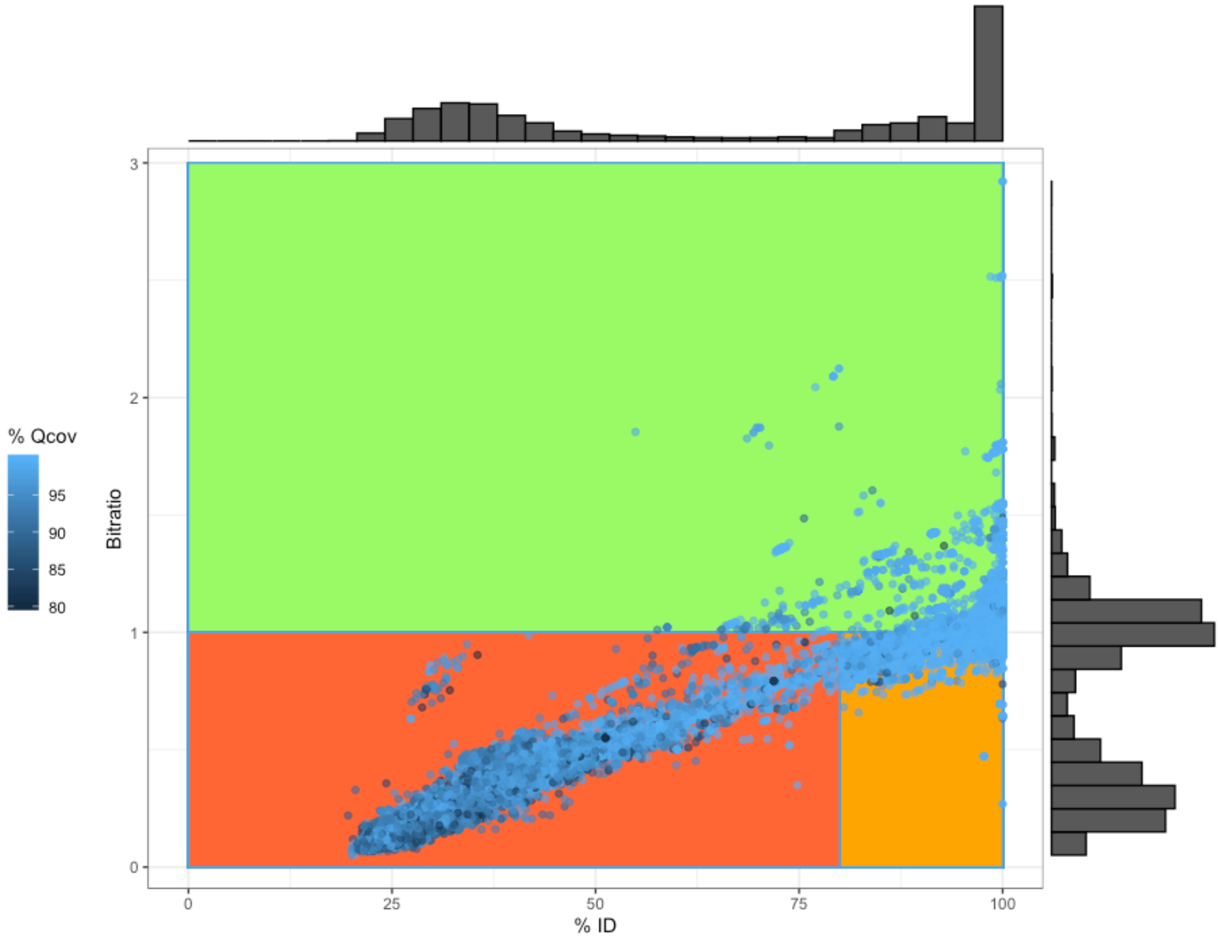

Supplementary Fig. 1. Distribution of DIAMOND blastp hits. All proteins from RefSeq complete genomes were used as queries against the CARD database. Before applying filters, the search cutoffs were at least 80% query coverage and an E-value of 10E-10. Subsequently, hits were filtered based on ARG-specific bitscores (curated by CARD/RGI) and by minimum 80% ID. Hits passing the RGI bitscore cutoff are located in the green area, while hits passing the custom 80% ID cutoff are located in the orange area. Hits not passing either filter are located in the red area. Bitratio on Y-axis is calculated by dividing individual bitscores by the curated bitscore-cutoffs from CARD/RGI. Histograms on the outside of the plot show the distribution of both %ID and bitratio.

Blastp hits passing the filters were investigated for phylogenetic distribution. By also including blastp hits that are more than 80% identical to a CARD protein, we include additional 61,620 hits on top of the 115,268 hits passing the RGI bitscore cutoffs. The major taxonomic orders are mostly equally included by the RGI bitscore filter and the 80% ID filter (Supplementary Fig. 2), with the biggest order being *Enterobacterales* that constitutes 25% and 49% of blastp hits passing the ID and RGI filter, respectively.

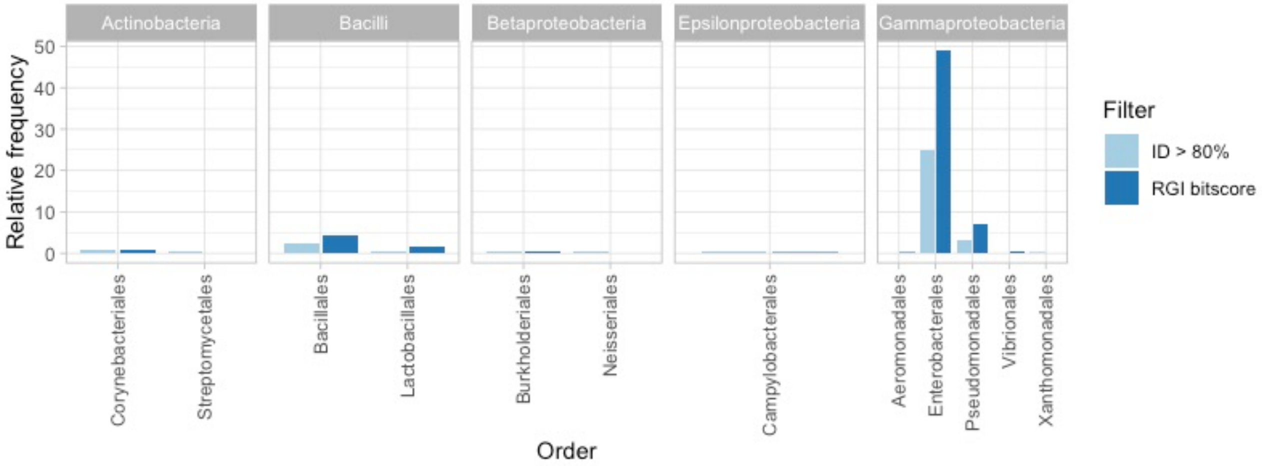

Supplementary Fig. 2. Major bacterial orders of ARG blastp hits passing either of the defined filters. On the Y-axis, the percentage of the total hits passing the respective filter is shown. Major orders are defined as orders that constitute more than 0.2% of the total data. In this plot, blastp hits can be in either ID filter or RGI filter, but not both (either bitscore > threshold or bitscore < threshold but ID > 80%).

However, the minor bacterial orders are not as equally distributed in RGI and ID filters as the major orders (Supplementary Fig. 3). Specifically, the orders *Micrococcales*, *Pseudonocardiales, Rhodospirillales*, *Aeromonadales*, *Alteromonadales*, *Legionellales*, and *Vibrionales* are passing the ID filter more than the RGI bitscore cutoff. This shows that including hits passing the additional ID filter expands the scope of this study to incorporate more environmental bacteria.

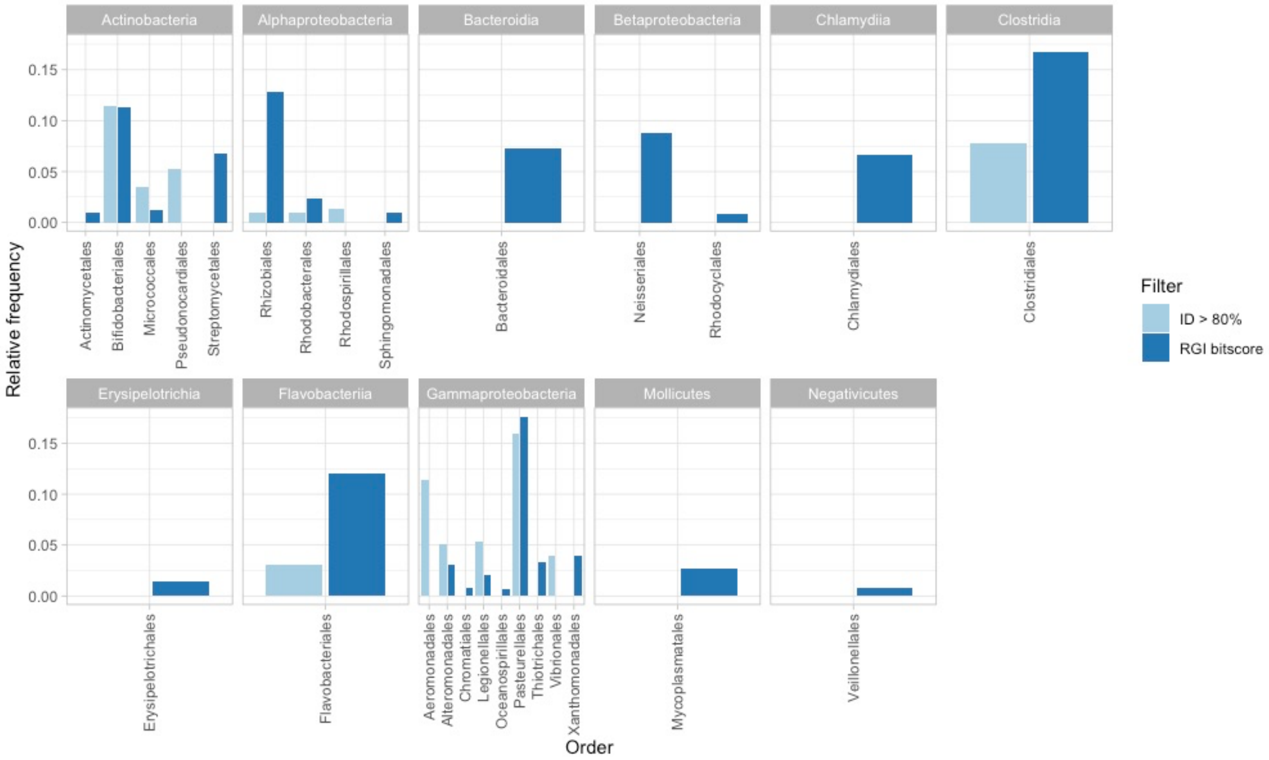

Supplementary Fig. 3. Minor bacterial orders of ARG blastp hits passing either of the defined filters. On the Y-axis, the percentage of the total hits passing the respective filter is shown. Minor orders are defined as orders that constitute less than 0.2% of the total data but only orders with more than 10 hits are shown here. In this plot, blastp hits can be in either ID filter or RGI filter, but not both (either bitscore > threshold or bitscore < threshold but ID > 80%).

Mean lengths of composite and unit transposons

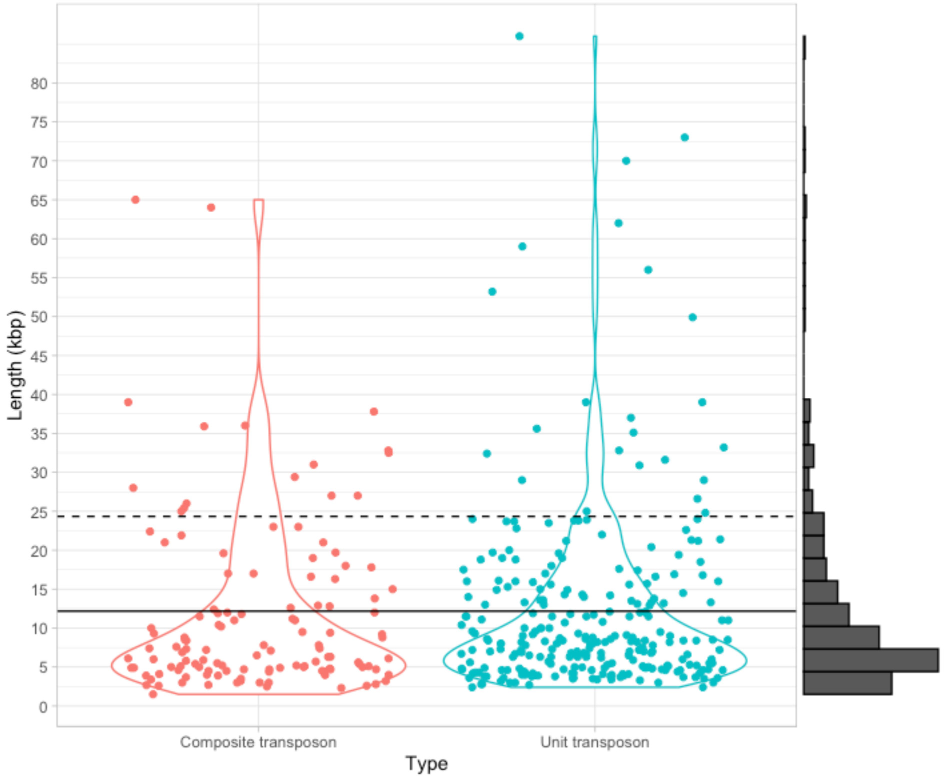

Supplementary Fig. 4. Violin plot of sequence lengths of 313 unit transposons and 136 composite transposons from The Transposon Registry [1]**.** The mean length (12.17 kbp) for both transposon types is shown as a solid line, and the maximum length of a genetic region with a putative ARG (24.34 kbp) is shown with a dashed line. A histogram of both length distributions combined is shown to the right. The individual mean lengths are 12.37 and 11.74 kbp for unit and composite transposons, respectively.

Supplementary Table 1. Cutoffs and search criteria for ARG and IS element prediction.

Rule	ARG hit	IS hit
Bitscore (DIAMOND)	Higher than RGI cutoffs	NA
E-value (DIAMOND)	10E-10	10E-30
Query coverage (DIAMOND)	80%	90%
Percent ID (DIAMOND)	80% (if bitscore < RGI cutoff)	NA
Maximum distance ARG-IS (SI)	No higher than the mean size (12.17 kbp) of all unit and composite transposons in The Transposon Registry [1]. The maximum distance is investigated in both directions of ARGs for IS elements and integrons.	

Supplementary Text 2: Database biases

In the RefSeq complete genomes, bacteria belonging to the Enterobacterales order make up the biggest order in the database and represent 20.42% of entries (Supplementary Fig. 5). Likewise, specialized functional gene databases, such as CARD and ISfinder, can be assumed to be biased towards certain taxonomic groups. Together, these biases will likely bias the analyses presented here synergistically by the combined biases the RefSeq, CARD, and ISfinder databases.

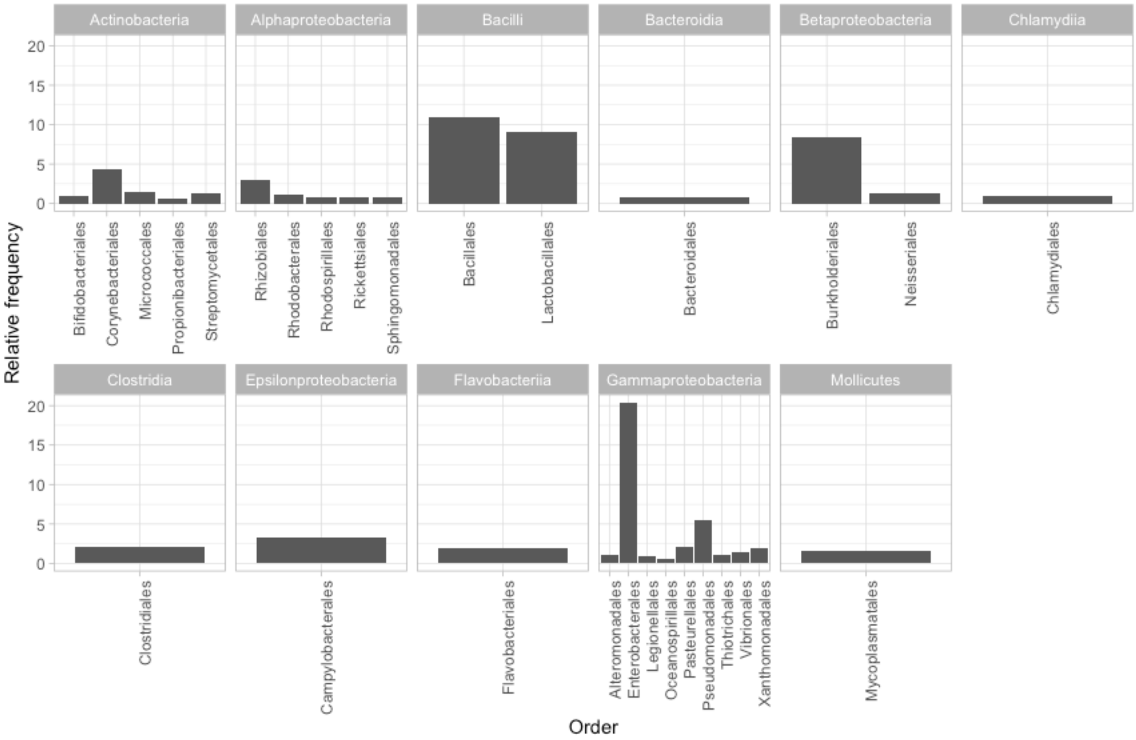

Supplementary Fig. 5. Frequencies (%) of RefSeq genomes by order taxonomic level. Only orders making up at least 0.5% of the total abundance are shown. Orders are organized by phylum.

Proteins in the CARD database are representatives from single strains of bacteria. Comparing relative abundances of represented genera in the CARD protein homology database with the relative abundances of the same genera in the RefSeq complete genome database shows that the two databases do not have an equal distribution of genera (Supplementary Fig. 6).

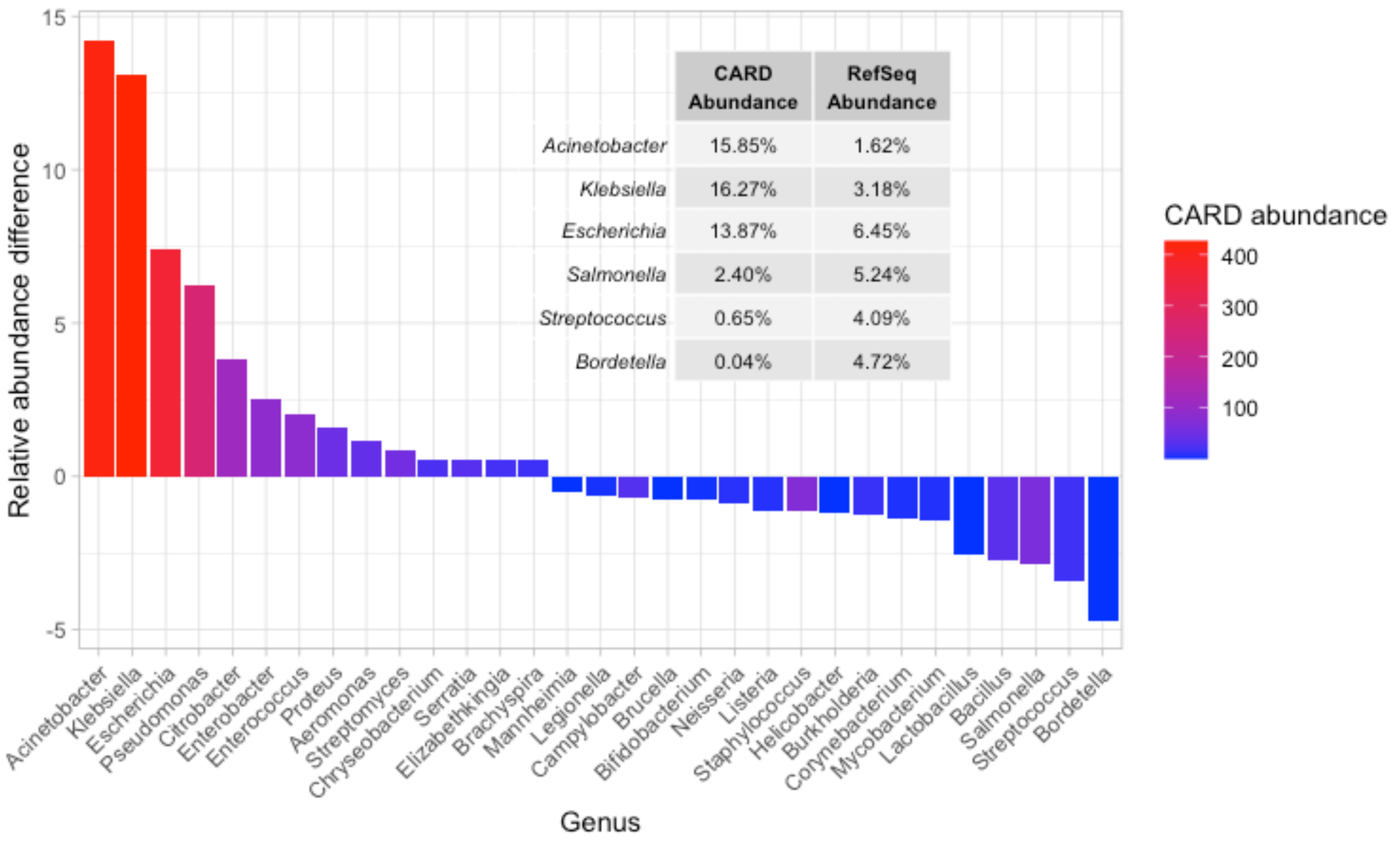

Supplementary Fig. 6. Differences in relative abundance in the representation of genera in CARD and RefSeq complete genomes databases. Positive difference indicates higher abundance in CARD than in RefSeq and vice versa. Only genera present in both databases and with difference in relative abundance of at least 0.5% are shown.

The 6 most extreme cases with biggest difference in relative abundance between CARD and RefSeq databases are highlighted in a table within Supplementary Fig. 6. Putative ARGs from *Acinetobacter* strains represent 15.6% of data in CARD, while *Acinetobacter* only accounts for 1.6% of entries in RefSeq complete genomes. Conversely, *Bordetella* genomes constitute 4.7% of RefSeq complete genomes, but are only represented by 0.04% of the CARD proteins. *Streptococcus* is likewise less abundant in CARD. All of the top 6 most different genera are all of potential clinical relevance and there is no apparent logical reason why one of these genera should have fewer or more inherent or acquired ARGs than the other. Considering the absolute abundance of the CARD genera, it seems likely that the differences in relative abundance between CARD and RefSeq is largely due to biased representation in CARD proteins. ARG prediction in underrepresented genera such as *Bordetella* and *Streptococcus* is therefore likely to be less accurate and encompassing than in e.g. *Acinetobater*, *Klebsiella*, and *Escherichia*.

Supplementary Text 3: Ameliorating database biases by clustering

Most publicly available sequence databases are biased in entries towards organisms that have gathered the most research interest, usually human-associated bacteria such as enterobacteria. It is therefore assumed that both CARD and RefSeq databases are heavily biased, but not towards the same genera. These biases will, naturally, affect analyses performed in this study. However, as one of the most curated and widely used ARG databases, CARD is the only obvious choice for this study. Likewise, RefSeq complete genomes comprise a large and well-curated database of publicly available genomes and is the natural choice for this study. Both RefSeq and CARD databases are generally biased towards the Enterobacteriales order but RefSeq is more skewed towards *Bordetella*, *Streptococcus*, and *Salmonella* than CARD. On the other hand, CARD entries are overrepresented by *Acinetobacter*, *Klebsiella*, and *Escherichia* compared to RefSeq (Supplementary Figs. 6 and 7A).

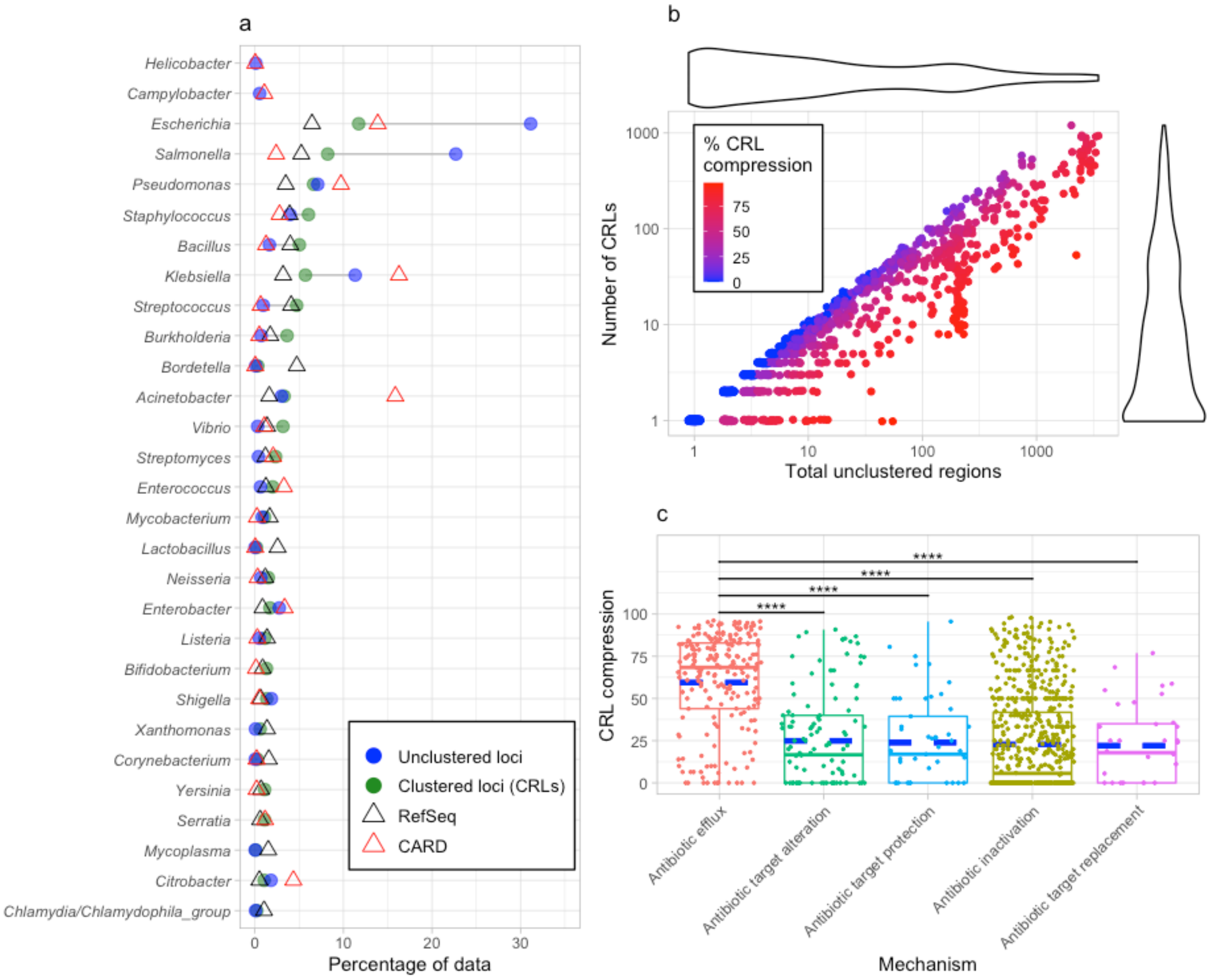

Supplementary Fig. 7: Effect of clustering ARG loci to CRLs. (A) Database content of most abundant genera and the effect of clustering to CRLs. Clustered loci (CRLs; green circles), divided into the parent genus, generally come closer to the relative abundance of the given genus in the

RefSeq complete genomes database (black triangles), than the unclustered, redundant ARG loci (blue circles). The relative abundance of genera from the CARD database are also shown (red triangles). Only genera that make up at least 1% of the total dataset of either unclustered loci, CRLs, or RefSeq complete genomes are shown. The displayed genera constitute 92.68%, 78.43%, 61.64%, and 81.52% of the total datasets for unclustered loci, CRLs, RefSeq, and CARD databases, respectively. (B) Compression effect of clustering ARG loci to CRLs. Each dot represents a single ARO category where its position on the x-axis indicates the number of identified unclustered loci and its position on the y-axis is the number of CRLs produced from clustering. The colour of the points shows the compression rate of clustering in percentage, calculated by dividing number of CRLs with the number of unclustered loci. Red points indicate that there are many, almost identical, ARG loci for a given ARO category, resulting in a low number of CRLs relative to the number of unclustered loci. Vice versa, blue points indicate that most of the unclustered loci are unique and clustering results in a number of CRLs that is close to the number of total unclustered loci. Violin plots are shown for both axes on the outside of the plot. Note that the position of points have been jittered very slightly to improve visualization. (C) The CRL compression rate per major resistance mechanism (the very low abundance 'Reduced permeability to antibiotic' category is not shown). Each jittered point is a unique ARO CRL. Boxplot beneath CRL points show interquartile range and median as solid horizontal line. The dashed blue line indicates the mean. Whiskers extend to 1.5 * of the interquartile ranges and outliers of this range are not highlight. Difference in means of CRL compression per resistance mechanism were tested with Mann-Whitney U-test with Holm-Bonferroni correction for multiple testing. Only significant differences are shown (**** P < 0.0001).

In order to minimize the impact of oversampling of almost identical genomes from RefSeq (e.g. *E. coli* substrains), ARG loci were clustered with USEARCH to 99% sequence similarity over at least 90% of the region length (length of ARG + 12,170 bp in both directions). 176,688 loci with ARGs passed all filters and were clustered to 53,895 CRLs that represent 1,176 individual CARD ARO categories out of a total of 2,617 in the CARD protein homolog database (Supplementary Table 2). The missing AROs are located in bacteria that do not yet have completed genomes or are so similar to one of the 1,176 identified AROs that they were not included, since only the best ARO match per query protein was considered. Indeed, removing the initial 1,176 AROs from the CARD database and performing the analyses again resulted in 336 AROs that were not included in the main analysis (results not shown).

Supplementary Table 2. Overview of number of hits and clusters. Of the 176,888 loci passing filters, 115,268 pass the RGI bitscore cutoff while 61,620 pass the 80% ID filter. All hits have at least 80% query coverage against the CARD ARGs.

Blastp hits against CARD (80% Qcov, E-value 10E-10)	DNA loci with ARG hits passing filters (RGI and/or ID > 80%)	Clustered CRLs	Total number of AROs represented

1,341,463	176,888	53,895	1,176

Genetic loci with ARGs are summarized based on the genus of the strain they occur in (Supplementary Fig.A). The relative abundance of each genus with ARGs are compared with the relative abundance in the RefSeq complete genome database. The differences between relative abundances per genus (given in percent of total database size) are summarized, resulting in a Euclidean distance of 30.89 (373 genera) between unclustered loci and RefSeq. When clustering these loci to CRLs, the Euclidean distance of relative genera abundances compared to RefSeq abundance is reduced to 10.26 (370 genera), which shows that clustering reduces the effect of oversampling of e.g. almost clonal *E. coli* strains. The most abundant genera, which make up at least 1% of either unclustered loci, CRLs, or RefSeq, are shown in Supplementary Fig.A. Almost all of the 29 genera shown are associated with either human pathogens or other anthropogenic activity (*Lactobacillus*, *Bifidobacterium*), showing that bacteria in these ecological niches are overrepresented in both the RefSeq and the CARD database. The CARD database is even more biased towards known pathogens including *Escherichia*, *Pseudomonas*, *Klebsiella*, *Acinetobacter*, and a few others (Supplementary Fig.A). This bias is sure to have a major effect on ARG prediction and leads to high ARG estimates in these genera compared to others (e.g. environmental bacteria). Clustering nearly identical ARG loci to CRLs definitely helps to smoothen this skew but it cannot completely even out the biases discussed here. Furthermore, clustering to CRLs reduces the overemphasis on the human-associated genera shown in Supplementary Fig.A from 92.68% of the total ARG loci to 78.43%, which slightly improves representation of other genera. These database biases are not surprising, since many researchers and clinicians are interested in human pathogens or closely related bacteria that can develop and transfer antimicrobial resistance. Therefore, we accept these biases in the present study but are aware that environmental bacteria and their potential resistance genes are underrepresented here.

Not all ARO categories are compressed equally in relative abundance by clustering loci to CRLs (Supplementary Fig.B and C). Some AROs are represented only by completely unique DNA loci in the unclustered dataset, resulting in a number of CRLs that is the same as the number of unclustered loci (low compression rate). On the other hand, other AROs are represented by a large number of nearly identical DNA loci which either stem from biased oversampling of e.g. almost clonal *E. coli* or by clonal expansion of one or more DNA loci by HGT (high compression rate).

Generally, loci with efflux pump resistance determinants have a significantly higher average CRL compression rate than the other functional categories (Figure 1, Supplementary Fig.C; $P < 0.0001$), which means that there are many almost clonal DNA loci with efflux pumps in the RefSeq complete bacterial genome database. The other general functional categories do not have a significantly different CRL compression rate, signifying that AROs belonging to these categories are located in more diverse DNA loci than efflux pumps are.

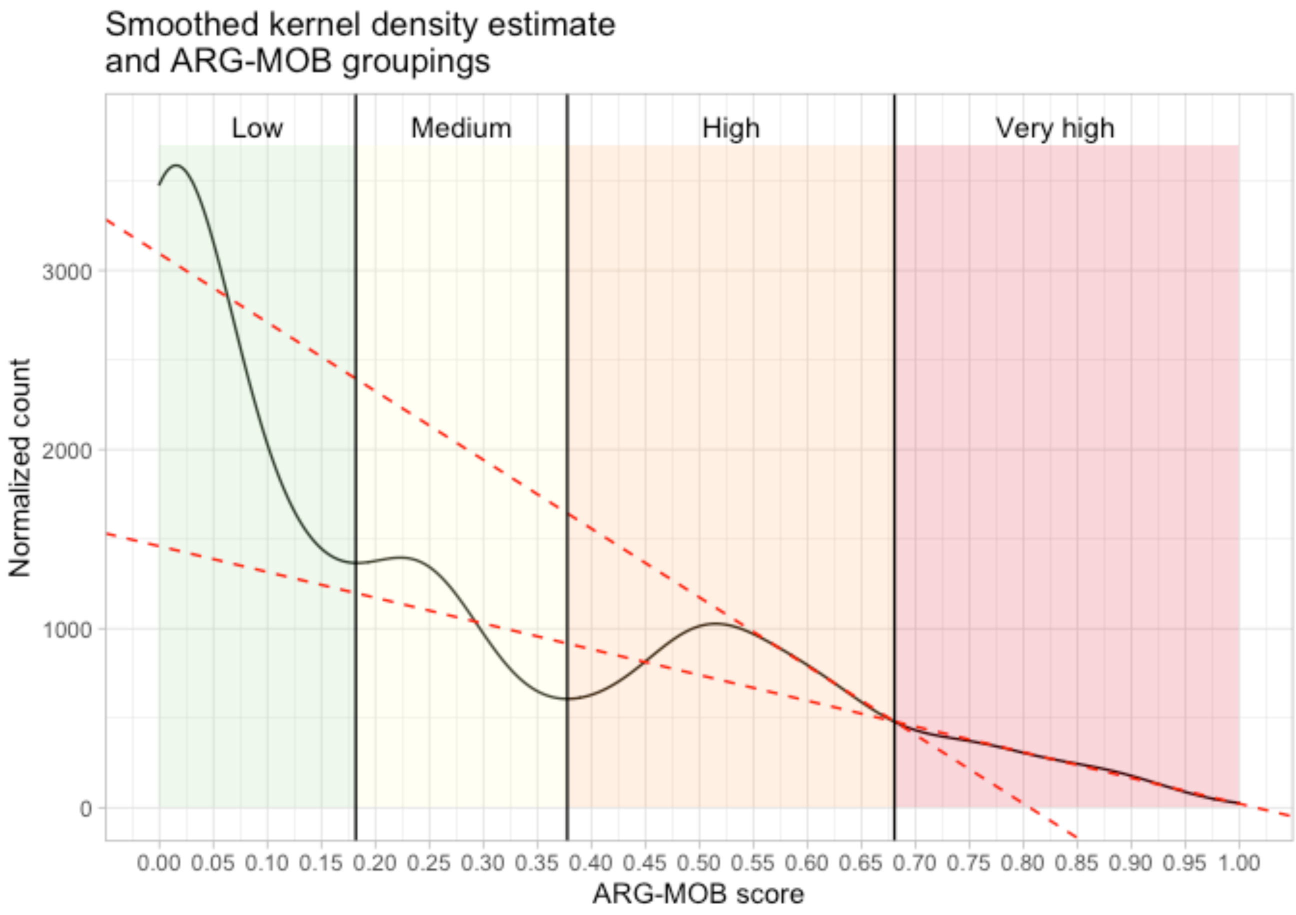

Supplementary Fig. 8. Smoothed kernel density estimates of all AROs and their ARG-MOB values. ARG-MOB values defining the categories were computed by identifying local minima in R. The High-Very high intersection is not found by this method. Instead, linear models were fitted to High interval 0.55-0.65 and Very high interval 0.7-1. These intervals are approximately linear. The intersection between the two models (0.685) is used as the High-Very high limit.

Supplementary Text 4: Distance between all 16S rRNA genes and closest IS elements

Using barrnap [18], 80,141 16S rRNA genes were identified in 15,790 strains in RefSeq complete genomes (mean 5.08 16S genes/genome). Only IS elements within 100 kbp of 16S rRNA genes were considered. Out of the 80,141 16S rRNA genes 4,480 had one or more IS elements within 12.17 kbp, corresponding to 5.59% (
Supplementary Fig. 9). This gives an acceptable accuracy of 94.61% when predicting associations between ARGs and IS elements.

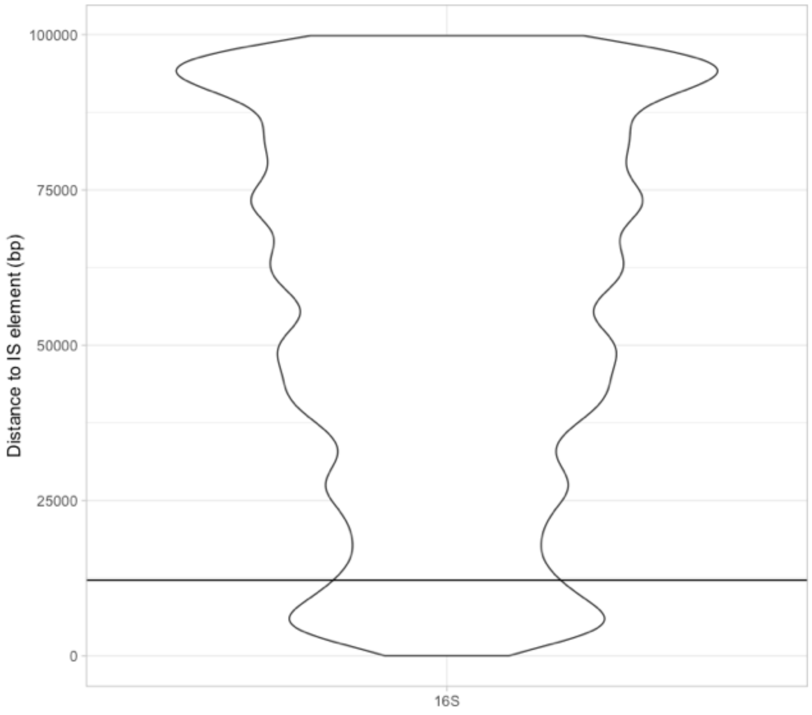

Supplementary Fig. 9. Violin plot of distances between all 16S rRNA genes and the closest IS element. Only 16S:IS associations within 100 kbp were plotted here.

Supplementary Text 5: IS element families in major mechanisms

The IS elements occurring in the proximity of putative ARGs were classified by their IS families and tested for significantly different relative abundances between resistance mechanisms (Supplementary Fig. **10**10). Among the 17 most abundant IS families, IS*1*, IS*110*, IS*1380*, IS*200*/IS*605*, IS*21*, IS*3*, IS*30*, IS*4*, IS*5*, ISL*3*, and Tn*3* occur with significantly different frequencies in *inactivation* and *efflux* loci, with lower median frequencies in *efflux*. These families are therefore more active in decontextualizing *inactivation* ARGs than *efflux*, accompanying the observation that *inactivation* is generally more mobilized than *efflux* ARGs. CRLs of the *replacement* mechanism are significantly less associated with a lack of IS elements, which in turn means that there are more CRLs of this mechanism with IS elements in proximity. This is also shown in Figure 3 and Supplementary Fig. 10 where it is apparent that this mechanism is very often found in association with IS elements and often on plasmids.

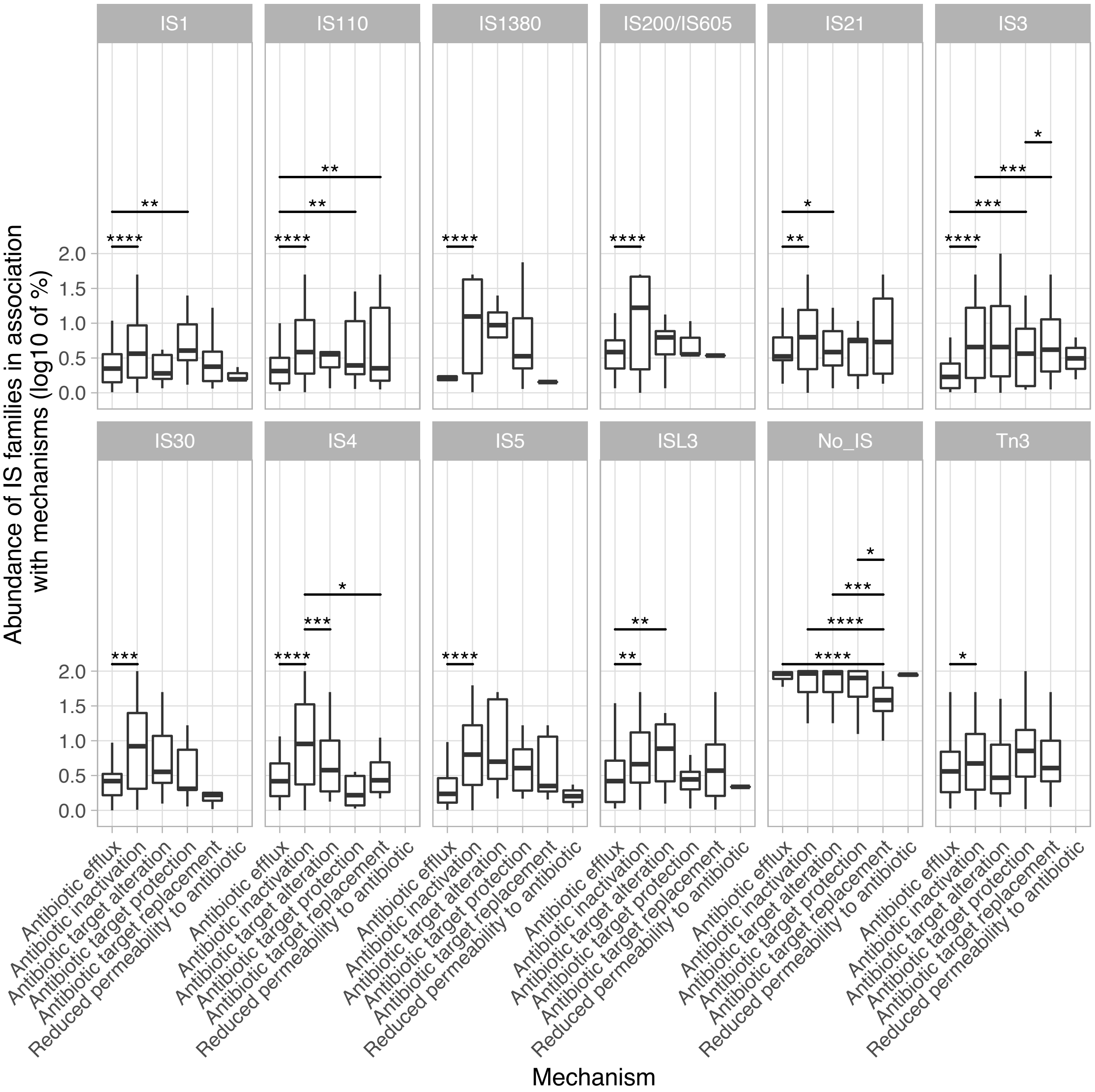

Supplementary Fig. 10. Relative abundance of 17 IS families and the lack of IS elements (No_IS) in ARO mechanisms. Only IS families with significant differences in mean value between mechanisms are shown. Log10 of relative abundance on Y-axes is derived from the frequency of IS families per mechanism category. Boxes indicate first and third quartiles (25% and 75% of data) and horizontal lines in boxes shows the median. Whiskers extend to 1.5 * of the interquartile ranges. Above boxplots, bars indicate significant differences in mean between mechanisms (Mann-Whitney test with Holm-Bonferroni correction). Only significant differences are displayed (*: p < 0.05;**: p < 0.01; ***: p <0.001; ****: p <= 0.0001).

Supplementary Text 6: Antibiotic efflux ARGs are more loosely associated with IS elements

Within the 12,170 bp investigated in both directions of identified ARGs, the distance to nearest IS element might be thought to be an indicator of how "tightly" associated a given ARG is with an IS element. Likely, there are some false positive associations between ARGs and IS elements found in the extremes of the 12,170 bp maximum distance. The mean distance between ARGs and IS elements per ARO is shown on the Y-axes in Supplementary Fig. 11a (mean distance per ARO is calculated from unique CRLs). For *efflux*, the bulk of the AROs have low IS ratios and a mean distance to closest IS elements of just over 5,000 bp. This is significantly different from 4 of the 5 other major mechanisms (Supplementary Fig. 11b), with the exception for the very low abundance Reduced permeability to antibiotic mechanism. The *efflux* AROs that do have higher IS ratios also have shorter distances to closest IS elements, which is comparable to AROs of other mechanisms that are also highly mobilized. For these mobilized AROs, the mean distance between ARGs and IS elements is closer to 2,500 bp.

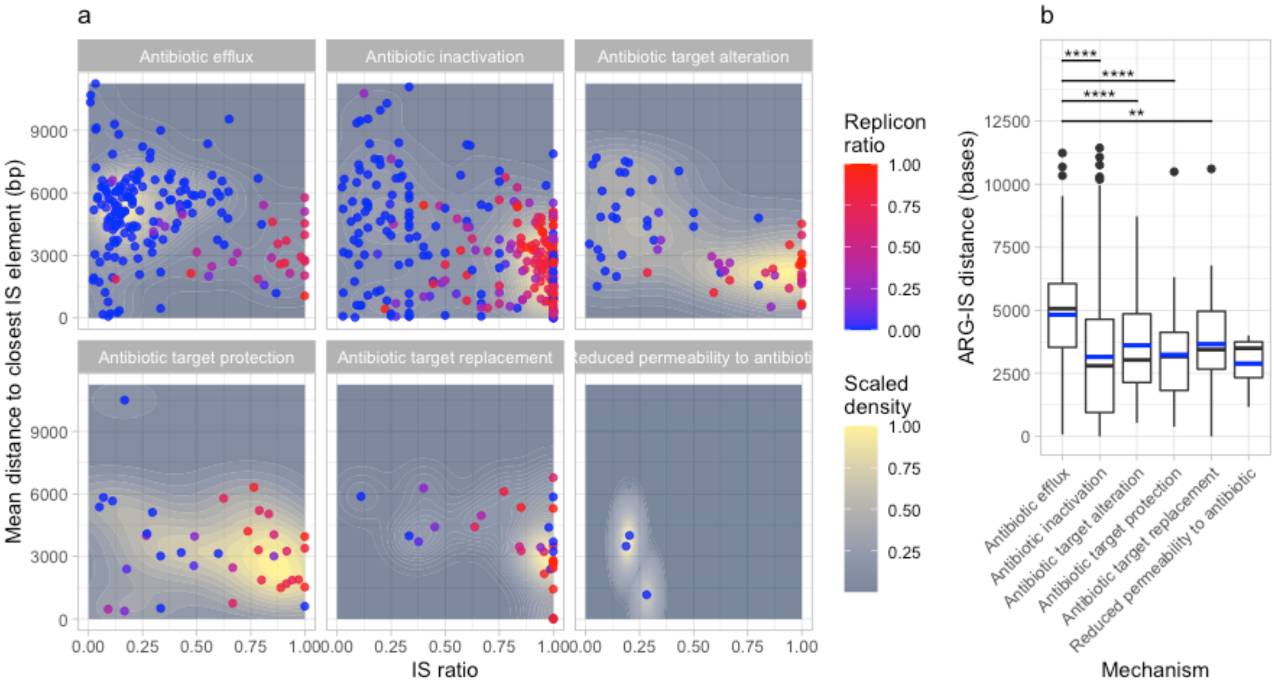

Supplementary Fig. 11. Distance between ARGs and closest IS element. (a) Density plots of IS ratio against the mean distance in bases to the nearest IS element in both directions. Each point represents a unique ARO category. Plots are divided into the individual mechanisms and are colored according to the replicon ratio, where a high ratio (red) indicates that an ARO is more often found on plasmids and a low ratio (blue) indicates that an ARO is more on chromosomes. Density estimates are calculated with two-dimensional kernel density estimation, as implemented in the stat_density_2d function under the ggplot R package. (b) Boxplot of median distance (bases) between ARGs and closest IS elements. Mean is shown with blue dashed lines. Boxes indicate first

and third quartiles (25% and 75% of data) and horizontal lines in boxes shows the median. Whiskers extend to 1.5 * of the interquartile ranges. Outliers are shown as black dots. Above boxplots, bars indicate significant differences in mean between mechanisms (Mann-Whitney test with Holm-Bonferroni correction). Only significant differences are displayed (**: p_{adj} <= 0.01, ****: p <= 0.0001).

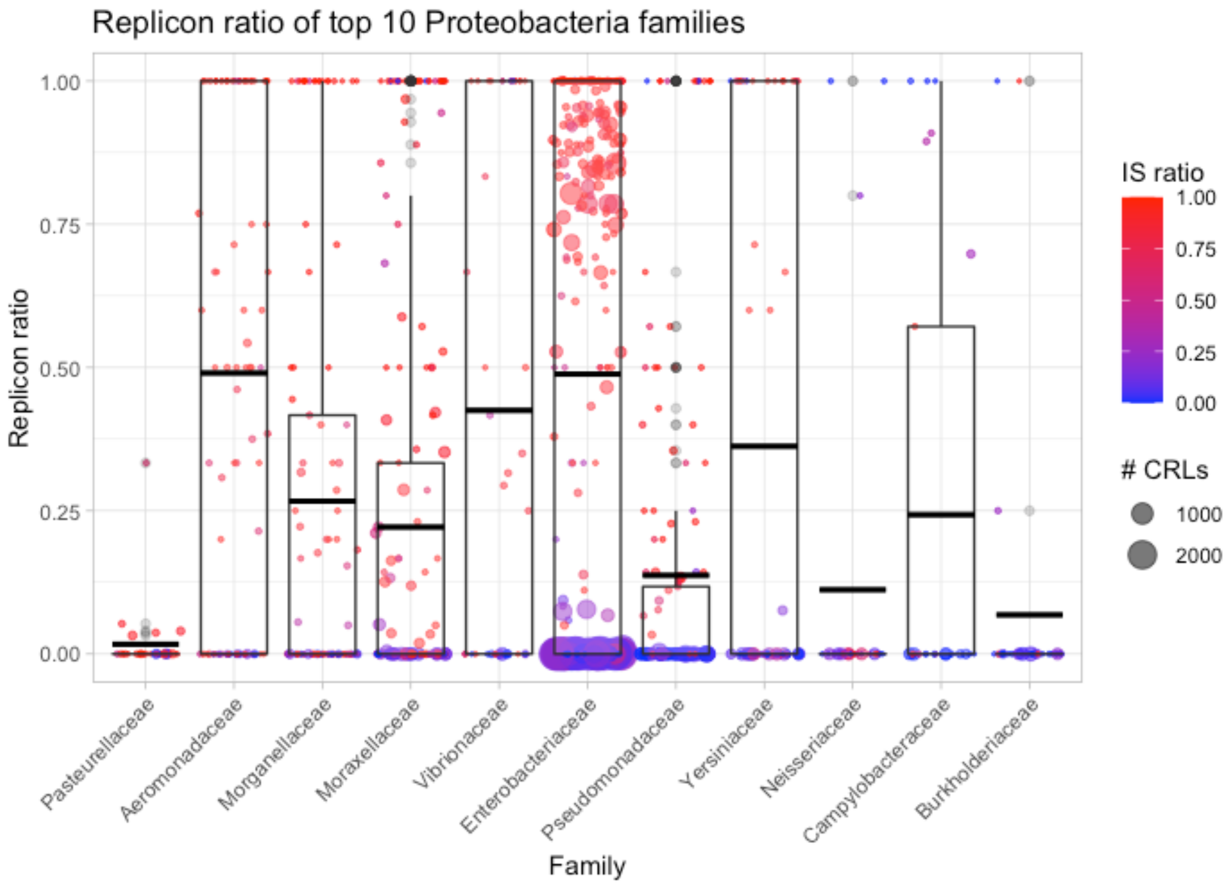

Supplementary Fig. 12. Replicon ratios of the top 10 most abundant Proteobacterial families. The colour of the CRL circles indicate IS ratio rather than the Replicon ratio shown in main figure 4.

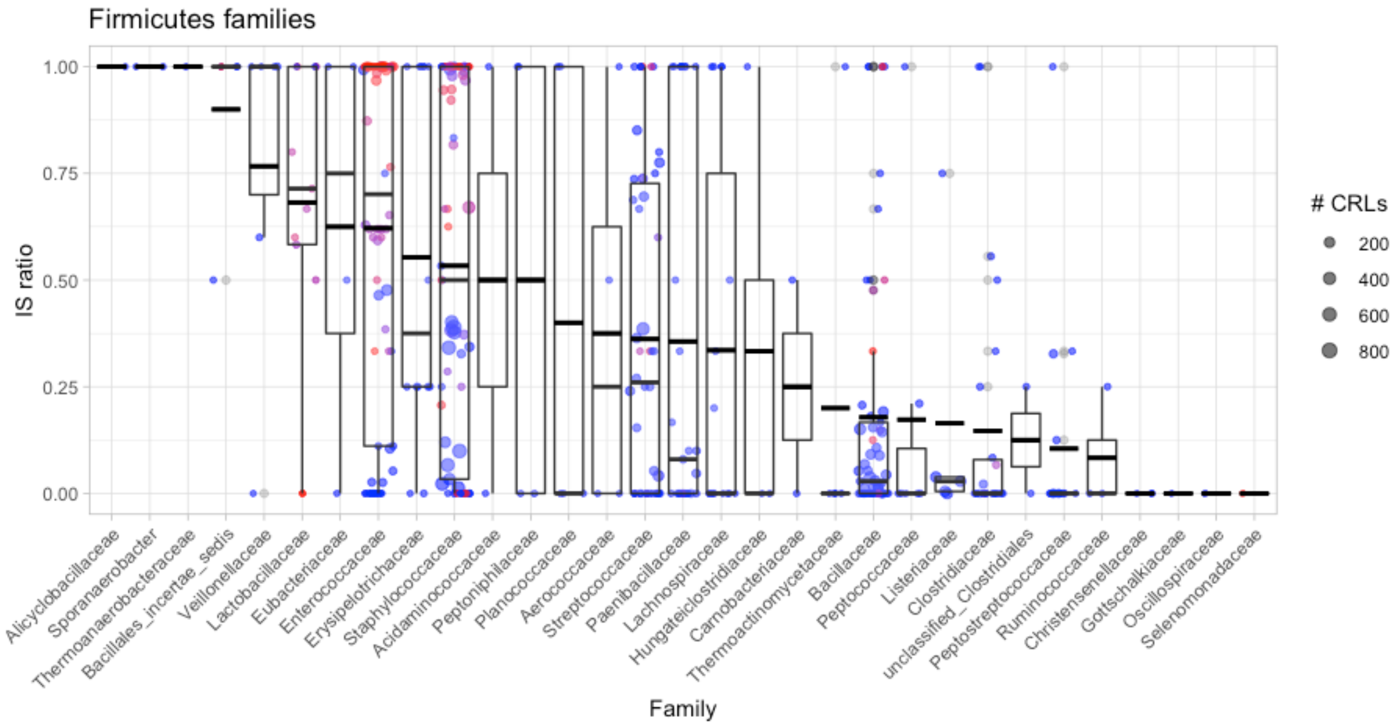

Supplementary Fig. 13. Taxonomic distribution of ARO categories within the Firmicutes phylum. Boxplots and dots show IS ratio per taxonomic group. The size of the points indicates the number of unique CRLs in a given ARO, while the colour is the replicon ratio with highest (red) indicating more plasmid than chromosome placement of CRLs Boxes indicate first and third quartiles (25% and 75% of data) and horizontal lines in boxes show the median. Whiskers extend to 1.5 * of the interquartile ranges.

Supplementary Table 3. Pairwise Mann-Whitney tests for IS and Replicon ratios in Enterobacteriaceae. *Holm-Bonferroni correction for multiple testing was applied. Only significant comparisons are listed here. Significance levels are *: p < 0.05; **: p < 0.01; ***: p < 0.001; ****: p < 0.0001.*

Genus 1	Genus 2	IS p	IS sign.	Replicon p	Replicon sign.
Cedecea	*Citrobacter*	9,10E-07	****	0,00015	***
Cedecea	*Cronobacter*	0,008	**	0,0064	**
Cedecea	*Enterobacter*	1,10E-07	****	3,50E-06	****
Cedecea	*Escherichia*	1,40E-13	****	2,20E-08	****
Cedecea	*Klebsiella*	7,80E-09	****	2,90E-07	****
Cedecea	*Leclercia*	0,00089	***	0,0049	**
Cedecea	*Raoultella*	0,00091	***	0,00053	***
Cedecea	*Salmonella*	7,20E-11	****	1,10E-06	****
Cedecea	*Shigella*	5,30E-13	****	0,0066	**
Citrobacter	*Escherichia*	0,006	**	NA	NA
Citrobacter	*Kluyvera*	0,032	*	NA	NA
Citrobacter	*Kosakonia*	2,50E-09	****	6,60E-06	****
Citrobacter	*Lelliottia*	2,80E-08	****	2,20E-06	****
Citrobacter	*Pluralibacter*	3,60E-10	****	6,60E-06	****
Cronobacter	*Escherichia*	0,022	*	NA	NA
Cronobacter	*Kosakonia*	0,00044	***	0,00097	***
Cronobacter	*Lelliottia*	0,0058	**	5,00E-04	***
Cronobacter	*Pluralibacter*	5,20E-05	****	0,00097	***
Enterobacter	*Kluyvera*	0,0048	**	NA	NA
Enterobacter	*Kosakonia*	1,80E-10	****	6,60E-08	****
Enterobacter	*Lelliottia*	2,10E-09	****	1,60E-08	****
Enterobacter	*Pluralibacter*	1,80E-11	****	6,60E-08	****
Enterobacter	*unclassified_Enterobacteriaceae*	0,039	*	NA	NA
Escherichia	*Kluyvera*	6,30E-07	****	0,0053	**
Escherichia	*Kosakonia*	9,70E-18	****	1,10E-10	****
Escherichia	*Leclercia*	0,031	*	NA	NA
Escherichia	*Lelliottia*	2,50E-17	****	1,60E-11	****
Escherichia	*Pluralibacter*	2,70E-18	****	1,10E-10	****
Escherichia	*Raoultella*	0,047	*	NA	NA
Escherichia	*unclassified_Enterobacteriaceae*	4,90E-06	****	NA	NA
Klebsiella	*Kluyvera*	0,0017	**	0,022	*
Klebsiella	*Kosakonia*	6,60E-12	****	2,50E-09	****
Klebsiella	*Lelliottia*	3,00E-11	****	4,40E-10	****
Klebsiella	*Pluralibacter*	1,20E-12	****	2,50E-09	****

Klebsiella	*unclassified_Enterobacteriaceae*	0,014	*	NA	NA
Kluyvera	*Pluralibacter*	0,046	*	NA	NA
Kluyvera	*Salmonella*	5,70E-05	****	NA	NA
Kluyvera	*Shigella*	2,60E-05	****	NA	NA
Kosakonia	*Leclercia*	2,20E-05	****	0,00069	***
Kosakonia	*Raoultella*	2,70E-05	****	4,40E-05	****
Kosakonia	*Salmonella*	1,80E-14	****	1,50E-08	****
Kosakonia	*Shigella*	5,30E-16	****	0,00097	***
Kosakonia	*unclassified_Enterobacteriaceae*	0,016	*	0,0038	**
Leclercia	*Lelliottia*	4,00E-04	***	0,00034	***
Leclercia	*Pluralibacter*	2,20E-06	****	0,00069	***
Lelliottia	*Raoultella*	0,00034	***	1,80E-05	****
Lelliottia	*Salmonella*	2,10E-13	****	3,00E-09	****
Lelliottia	*Shigella*	8,20E-17	****	0,00049	***
Pluralibacter	*Raoultella*	3,30E-06	****	4,40E-05	****
Pluralibacter	*Salmonella*	2,00E-15	****	1,50E-08	****
Pluralibacter	*Shigella*	1,10E-15	****	0,00097	***
Pluralibacter	*unclassified_Enterobacteriaceae*	0,0038	**	0,0038	**
Salmonella	*unclassified_Enterobacteriaceae*	0,00058	***	NA	NA
Shigella	*unclassified_Enterobacteriaceae*	0,00069	***	NA	NA
Cedecea	*unclassified_Enterobacteriaceae*	NA	NA	0,021	*
Escherichia	*Shigella*	NA	NA	0,0063	**
Klebsiella	*Shigella*	NA	NA	0,039	*
Kluyvera	*Lelliottia*	NA	NA	0,042	*
Lelliottia	*unclassified_Enterobacteriaceae*	NA	NA	0,002	**

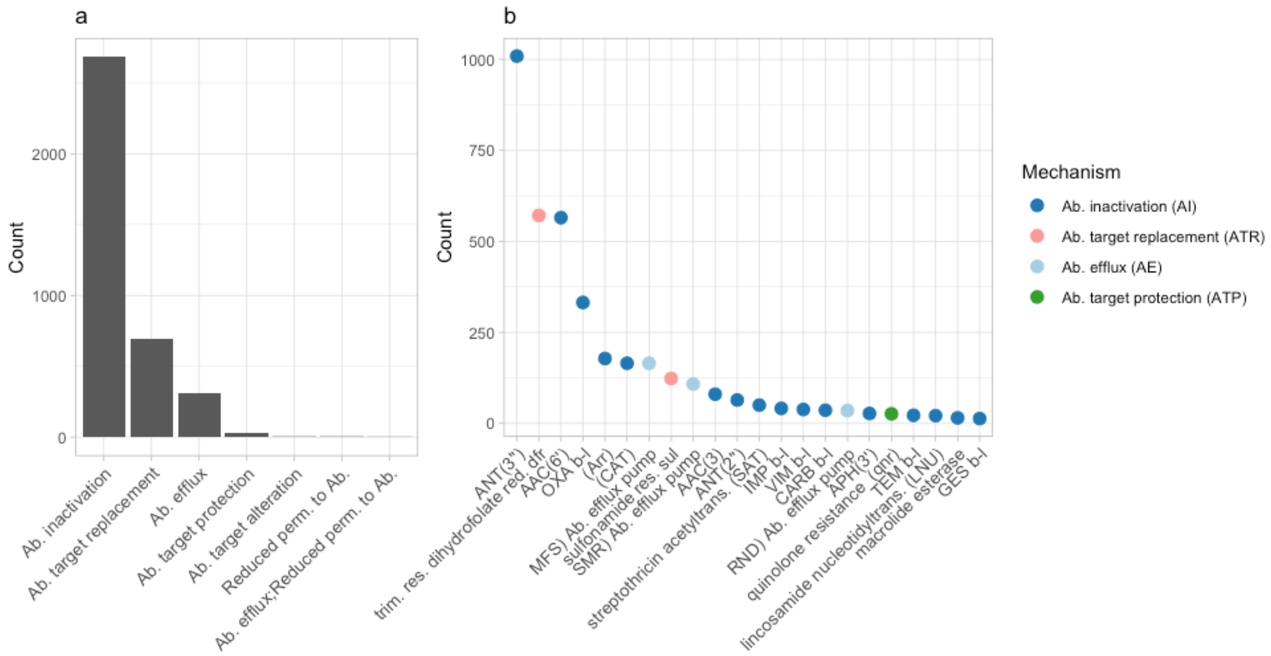

Supplementary Fig. 14. ARGs associated with integrons. (A) Major mechanisms associated with integrons. The count is in unique CRLs belonging to each mechanism. (B) CRLs where the identified ARG is inserted in an integron, summarized by submechanism category. Each point can include multiple ARO categories. Only submechanisms with more than 10 CRLs are shown.

Supplementary Table 4. The top 20 AROs associated with integrons.

Gene	ARO	Mechanism	Submechanism	Total CRL occurrences	CRLs associated with an integron (% of ARO total)	Percentage of total integron occurrences
aadA	3002601	Antibiotic inactivation	ANT(3'')	429	370 (86.2%)	13.24%
aadA2	3002602	Antibiotic inactivation	ANT(3'')	399	346 (86.7%)	12.38%
AAC(6')-Ib-cr	3002547	Antibiotic inactivation	AAC(6')	224	224 (100%)	8.01%
dfrA12	3002858	Antibiotic target replacement	trimethoprim resistant dihydrofolate reductase dfr	221	221 (100%)	7.91%
OXA-1	3001396	Antibiotic inactivation	OXA beta-lactamase	277	177 (63.9%)	6.33%
dfrA14	3002859	Antibiotic target replacement	trimethoprim resistant dihydrofolate reductase dfr	166	138 (83.1%)	4.94%
aadA5	3002605	Antibiotic inactivation	ANT(3'')	141	138 (97.9%)	4.94%
AAC(6')-Ib10	3002581	Antibiotic inactivation	AAC(6')	144	135 (93.7%)	4.83%
arr-3	3002848	Antibiotic inactivation	rifampin ADP-ribosyltransferase (Arr)	126	125 (99.2%)	4.47%
sul1	3000410	Antibiotic target replacement	sulfonamide resistant sul	1173	123 (10.5%)	4.40%

dfrA1	3002854	Antibiotic target replacement	trimethoprim resistant dihydrofolate reductase dfr	134	123 (91.8%)	4.40%
qacH	3003836	Antibiotic efflux	small multidrug resistance (SMR) antibiotic efflux pump	109	108 (99.1%)	3.86%
catB3	3002676	Antibiotic inactivation	chloramphenicol acetyltransferase (CAT)	101	100 (99%)	3.58%
cmlA1	3002693	Antibiotic efflux	major facilitator superfamily (MFS) antibiotic efflux pump	84	84 (100%)	3.01%
AAC(6')-Ib9	3002580	Antibiotic inactivation	AAC(6')	98	74 (75.5%)	2.65%
ANT(3'')-IIa	3004089	Antibiotic inactivation	ANT(3'')	94	67 (71.3%)	2.40%
OXA-9	3001404	Antibiotic inactivation	OXA beta-lactamase	67	66 (98.5%)	2.36%
ANT(2'')-Ia	3000230	Antibiotic inactivation	ANT(2'')	118	64 (54.2%)	2.29%
AAC(6')-Ib7	3002578	Antibiotic inactivation	AAC(6')	81	58 (71.6%)	2.08%
cmlA5	3002695	Antibiotic efflux	major facilitator superfamily (MFS) antibiotic efflux pump	58	54 (93.1%)	1.93%

Supplementary Text 7: Pearson correlation coefficient analyses for major mechanisms

Per major mechanism, Pearson correlation coefficients were also calculated pairwise between each of the four MOB parameters (Supplementary Figs. 15-19). For all mechanisms, IS and Replicon ratios correlate with similar correlation coefficients. ARG-IS association likewise correlates with ARG-integron association, except for the *target alteration* mechanism. For mechanisms *inactivation* and *efflux*, all correlations are positive and significant. For *target replacement*, the Simpson index correlates with neither IS nor integron ratio. For *target alteration* and *target protection*, four and two correlations were not significant, respectively. Numerically, *efflux* CRLs are most often found on chromosomes with no association with IS elements or integrons (Supplementary Fig. 20). Contrary to this, *inactivation* and *target replacement* CRLs are very often found on plasmids in association with both IS elements and integrons. Overall, there is also an evident association between integrons and IS elements, while integrons without IS elements is a rare combination (Supplementary Fig. 20).

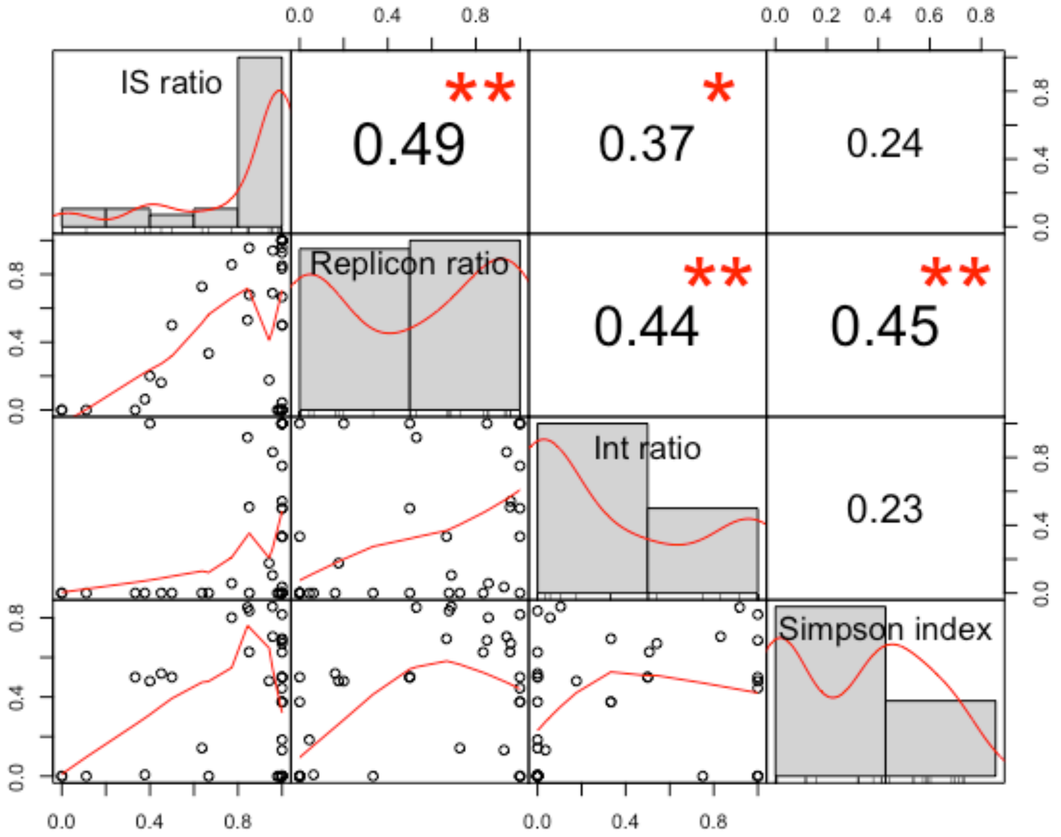

Supplementary Fig. 75. Pearson correlation coefficients for Antibiotic target replacement.

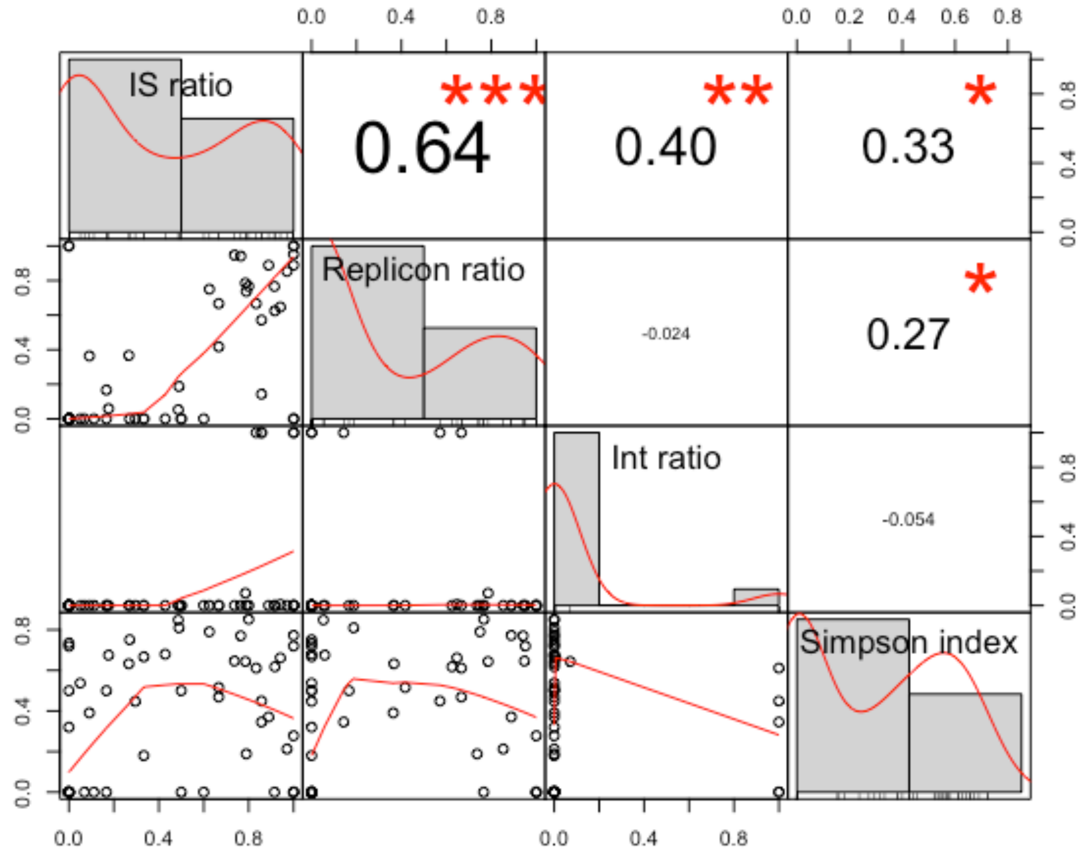

Supplementary Fig. 86. Pearson correlation coefficients for Antibiotic target protection.

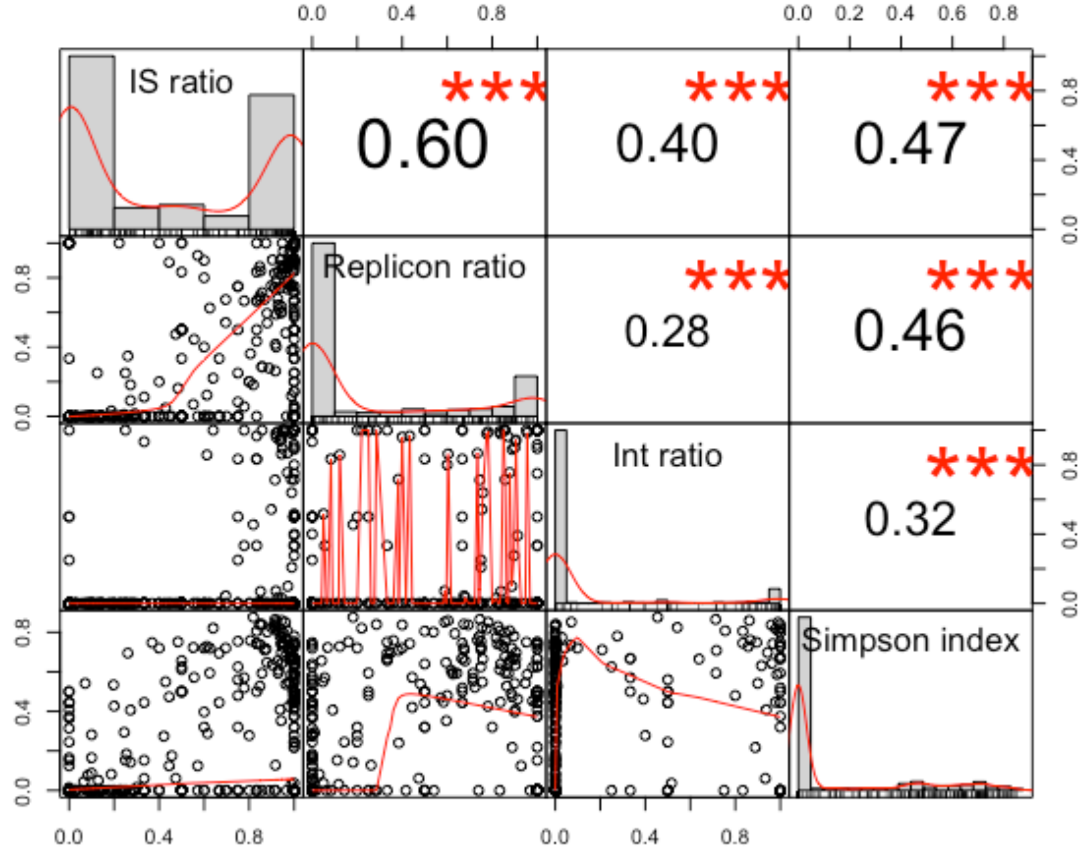

Supplementary Fig. 97. Pearson correlation coefficients for Antibiotic inactivation.

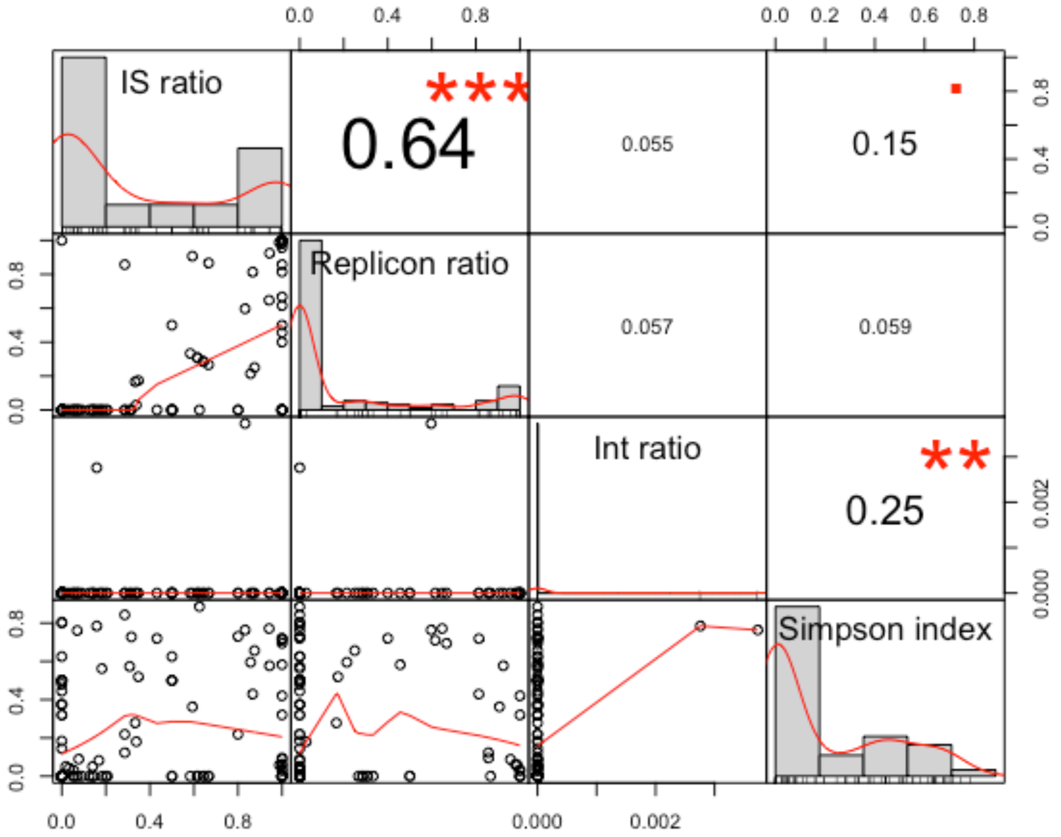

Supplementary Fig. 108. Pearson correlation coefficients for Antibiotic target alteration.

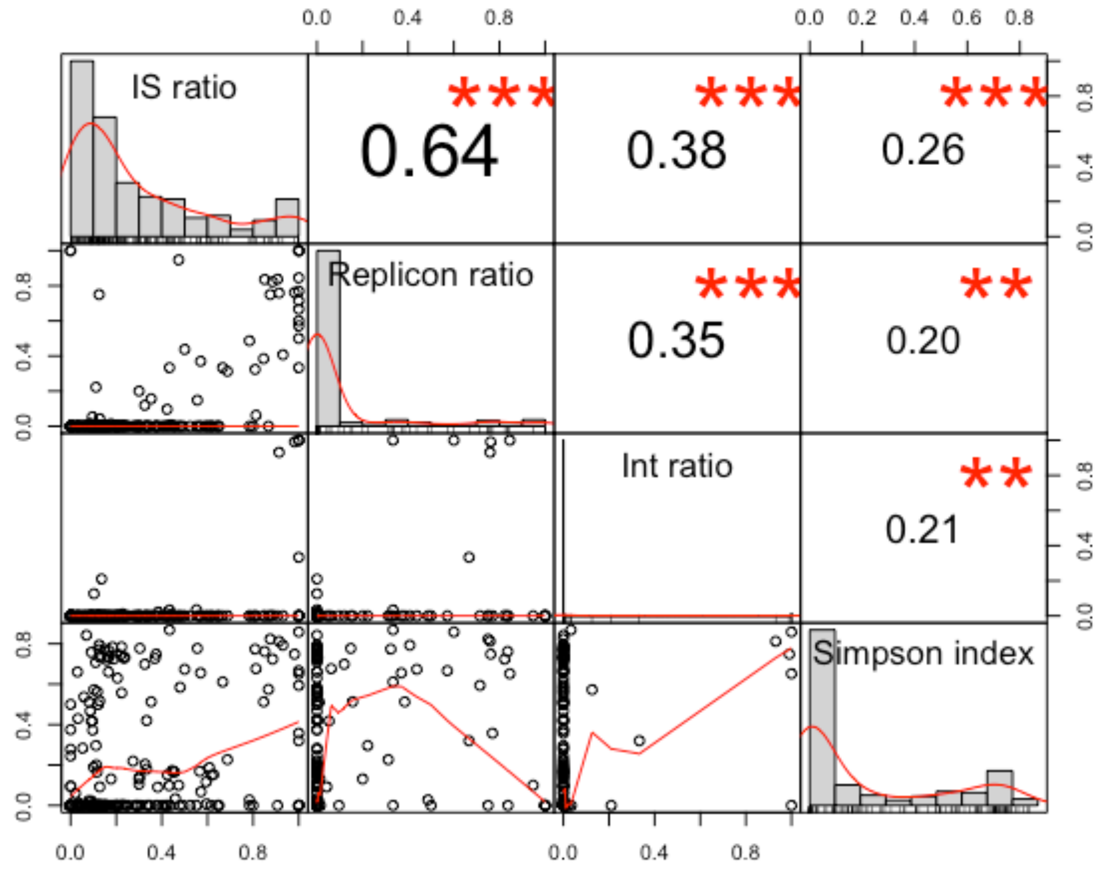

Supplementary Fig. 119. Pearson correlation coefficients for Antibiotic efflux.

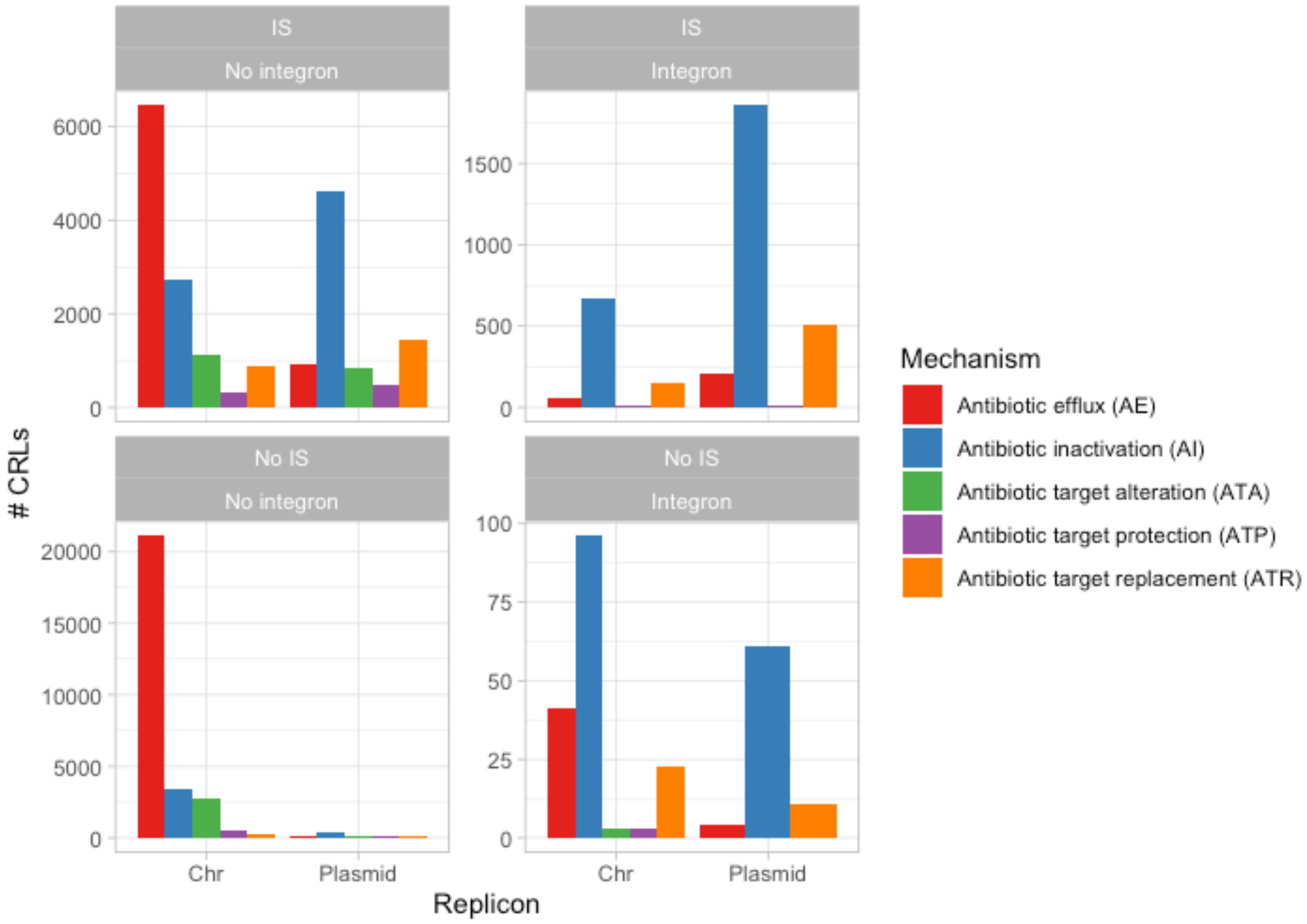

Supplementary Fig. 20: Counts of CRLs separated by replicon type, and IS- and integron association. Bars are coloured by resistance mechanism. Y-axis scales are different between each subplot.

Supplementary Text 8: Some AROs are highly divergent in mobilization

The mean IS and Replicon ratios per ARO are calculated across all genera they are found in. However, upon closer scrutiny of AROs per genus it becomes obvious that some AROs have a high spread from their IS and Replicon ratio means. For Supplementary Fig. 121, the genus-specific ratios were calculated per ARO and their difference from the mean global ARO ratio was calculated per genus. For each ARO, the summed positive and negative differences (for each genus with a given ARO) are shown below. A positive difference from the ARO mean indicates that there are genera in which ARGs of the given ARO are more mobilized than the ARO mean. Vice versa, a negative summed difference from the mean shows that ARGs of a given ARO are less mobilized in some genera than the ARO mean.

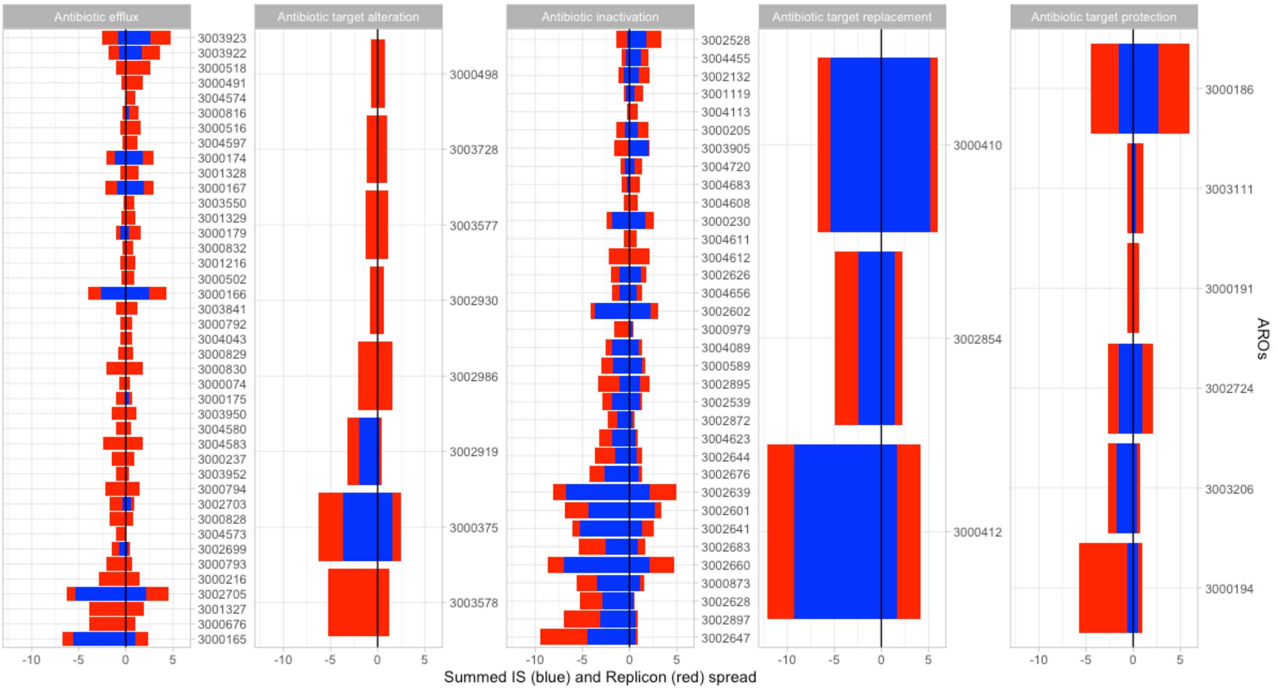

Supplementary Fig. 121: Summed spread from of mean IS and Replicon ratios from the ARO means. Differences from the mean per ARO were summed by the genera in which the AROs were identified. A positive summed difference from ARO mean indicates that some genera have more mobilized ARGs of a given ARO than the ARO mean. Mobilization by both IS elements (left) and plasmids (right) are shown. Before calculating the genus-specific IS and Replicon ratio per ARO, genus:ARO combinations with only 1 occurrence in the dataset were filtered, since they are a source of noise in this context. Only AROs with a summed negative plus positive spread from mean of at least 1 are shown.

From the same dataset, genera are plotted with their summed spread from the mean of all AROs found within the respective genera (Supplementary Fig. 132). Especially *inactivation* AROs in *Shigella* are highly mobilized by IS elements compared to the ARO means. Interestingly, the *inactivation* AROs are not very mobilized by plasmids in *Shigella*, indicating that *inactivation* ARGs are often associated with IS elements in *Shigella* but mostly on chromosomes. Also worth noting, *Proteus*, *Pseudomonas*, *Morganella*, *Acinetobacter*, and a few other genera have large negative summed Replicon spreads from mean, indicating that chromosomes in these may act as reservoirs for yet unmobilized ARGs. On the other hand, these putative and potential ARGs are not decontextualized and will likely occur as false-positive resistance genes in studies applying (q)PCR and metagenomic sequencing in environmental samples with these bacteria.

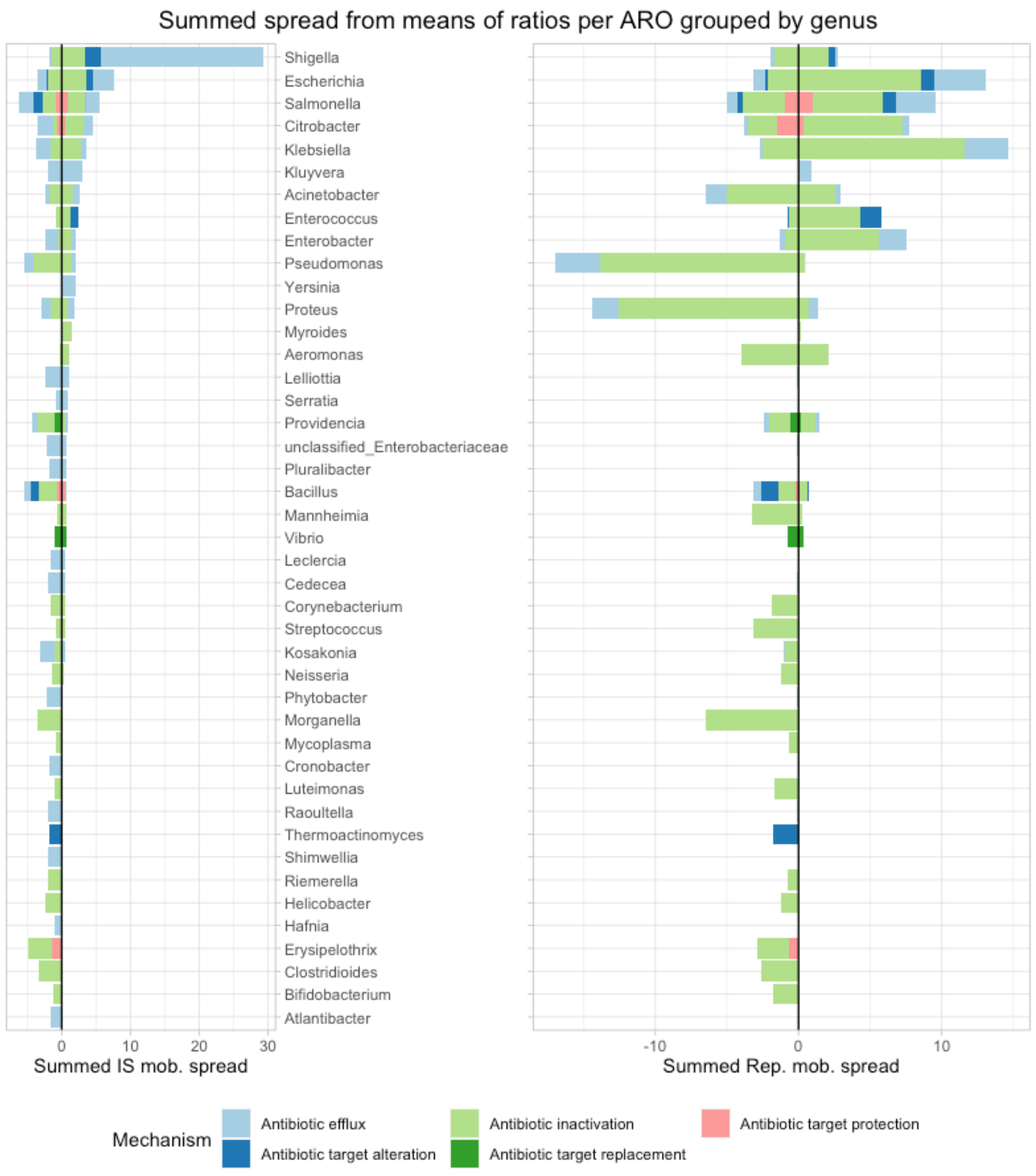

Supplementary Fig. 132: Summed spread from of mean IS and Replicon ratios from the ARO means grouped by genera. The same dataset as for figure Supplementary Fig. 21 is used here, but the summed spreads are grouped by genera. A large positive summed spread indicates that a given genus has AROs that are more mobilized, by either IS elements or plasmids, than the ARO mean.

In Supplementary Table 5, the efflux pump genes *oqxAB* is shown as an example of an ARO that is highly differential in IS and plasmid mobilization, depending on the genus it is located in. This pump is encoded by the neighbouring genes *oqxA* and *oqxB* that have the same mobilization characteristics. Only results for *oqxA* are shown in this example, since *oqxB* has similar results (not shown). In *Klebsiella* genomes, *oqxA* is found in association with IS elements in 15.5% of cases and

it is only on plasmids in 0.2%. As such, it should be considered a housekeeping gene, since it does not usually confer resistance unless highly overexpressed [2–4].

Supplementary Table 5. Information about the oqxA ARO.

**Genus**	**#Identified in total**	**# Found on chromosome**	**# Found on plasmid**	**# No IS association**	**IS Ratio**	**Replicon ratio**
Klebsiella	458	457	1	387	0.155	0.002
Enterobacter	124	123	1	111	0.105	0.008
Escherichia	36	2	34	0	1.000	0.944
Salmonella	20	3	17	0	1.000	0.850
Raoultella	14	14	0	13	0.071	0.000
Citrobacter	8	8	0	8	0.000	0.000
Kosakonia	7	7	0	4	0.429	0.000
Lelliottia	6	6	0	6	0.000	0.000
Cedecea	5	5	0	3	0.400	0.000
Phytobacter	2	2	0	2	0.000	0.000
Unclassified *Enterobacteriaceae*	2	2	0	2	0.000	0.000

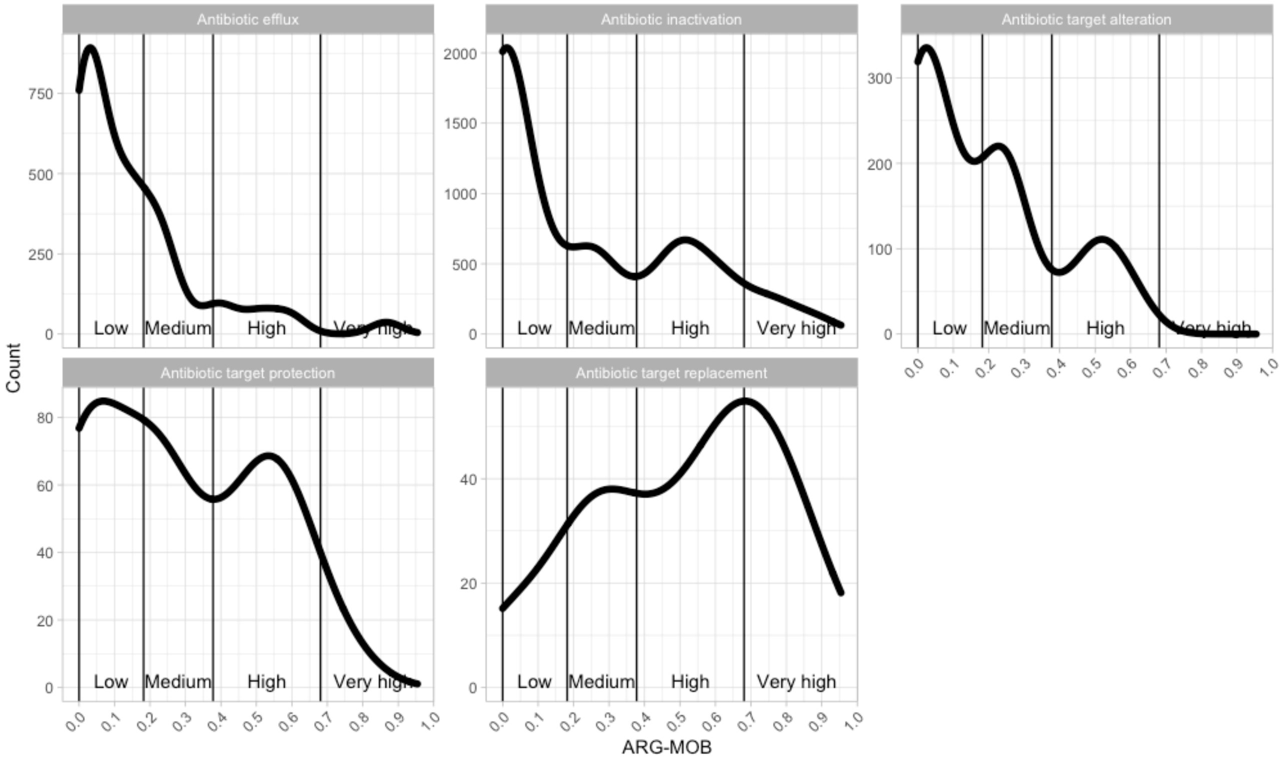

Supplementary Fig. 143: Count density of ARG-MOB per mechanism. Y-axes are not on the same scale between subplots. The global ARG-MOB category definitions are shown with vertical lines.

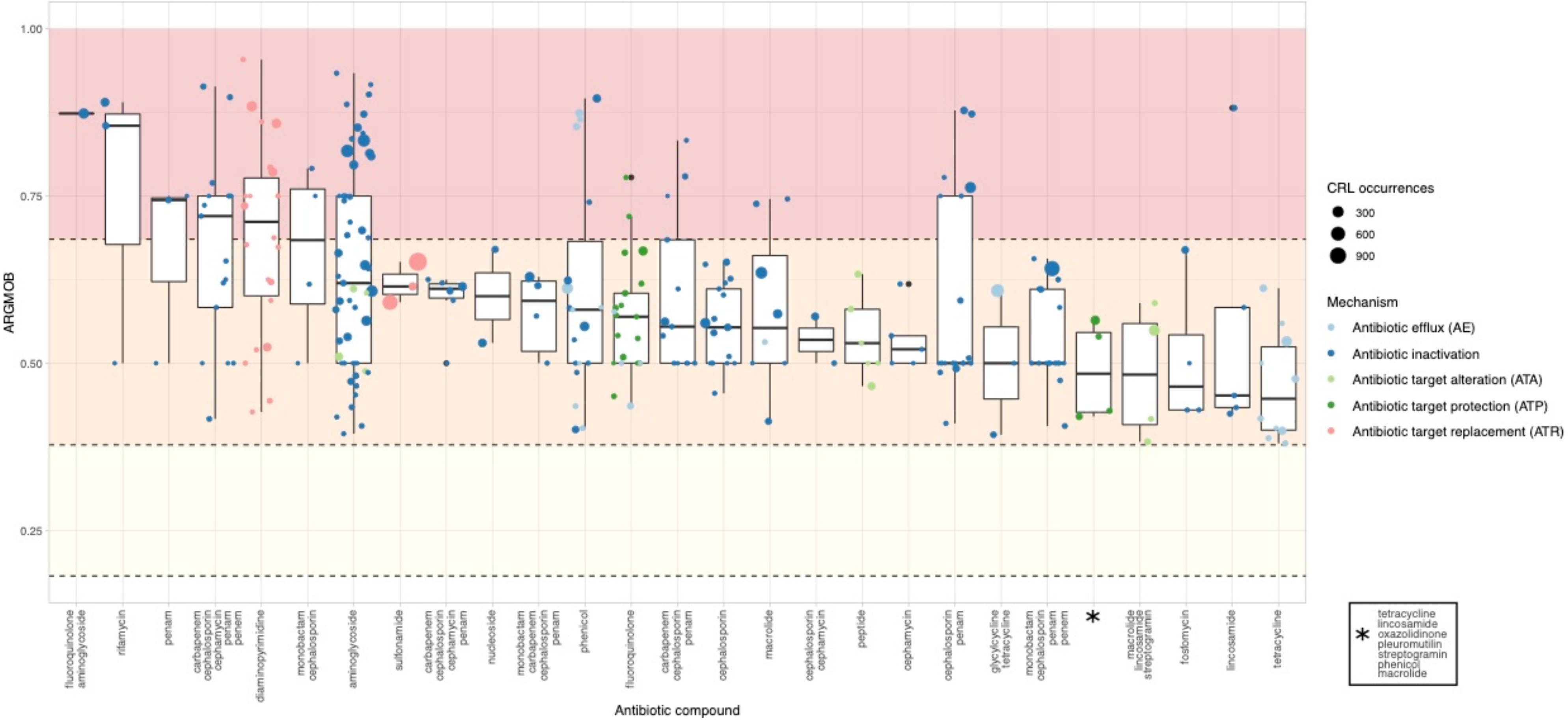

Supplementary Fig. 24. Antimicrobial compounds to which there are ARGs with *Very high* or *High* ARG-MOB scores. ARGs of different major mechanisms can confer resistance towards the same compounds, resulting in some compounds with more than one Mechanism.

Supplementary Text 9: Examples of Low and High ARG-MOB AROs

***Low* ARG-MOB examples**

ARG-MOB=0; 3003785 (*target alteration*) MurA transferase: Shown to confer resistance when cloned onto pBAD18 under overexpression in *E. coli* from *Chlamydia* [5].

ARG-MOB=0; 3000421 (*efflux*) NorB major facilitator superfamily (MFS) antibiotic efflux pump: Requires overexpression for resistance to quinolones, tetracycline and others. Was cloned from *Staphylococci* into *E. coli* for overexpression to give resistance [6].

ARG-MOB=0; 3000462 (*inactivation*) MgtA mgt macrolide glycotransferase resistance: Was shown to confer resistance to macrolides in *Streptomyces lividans* under overexpression from multicopy plasmid pLST21 with constitutive expression [7].

ARG-MOB=0; 3004143 (*efflux*) resistance-nodulation-cell division (RND) antibiotic efflux pump: The RND efflux pump AxyXY-OprZ is repressed by the upstream transcriptional repressor *axyZ*. Gene deletion mutation of *axyZ* leads to increased *axyZ* transcription and increased MICs of multiple antibiotics (fluoroquinolones, cefepime, tetracyclines) in *Achromobacter* [8]. The AxyXY are orthologs of MexX and MexY in *Pseudomonas aeruginosa* where mutations in the *mexZ* transcriptional regulator leads overexpression of the MexXY efflux pump and increased resistance [9]. These pumps are likely naturally occurring pumps that require significant overexpression to yield problematic resistance.

ARG-MOB=0; 3004775 (*inactivation*) CME beta-lactamase: CME-1 was cloned from a *Flavobacterium* and overexpressed from a cloning vector in *E. coli* to provide resistance to cefuroxime [10]. The strain was not a clinical isolate.

ARG-MOB=0; 3003035 (*target protection*) MfpA quinolone resistance protein (qnr): MfpA from *Mycobacterium tuberculosis* strain H37Rv protects DNA gyrase from fluoroquinolone

inhibition. It was found to confer low-level resistance when overexpressed from a multicopy plasmid [11], but strain H37Rv is not inherently resistant to fluoroquinolones [12].

ARG-MOB=0; 3002646 (*inactivation*) APH(3') aminoglycoside phosphotransferase: Aph(3')-IIc was cloned and overexpressed from the chromosome of *Stenotrophomonas maltophilia* in *E. coli* to confer increased MICs to amikacin, butirosin, kanamycin, lividomycin, neomycin, paromomycin, and tobramycin. In this study, Aph(3')-IIc was knocked out on the chromosome of the WT strain and MICs to butirosin, kanamycin, neomycin and paromomycin were decreased, showing the this gene indeed confers intrinsic resistance those antibiotics. While expression of Aph(3')-IIc was evaluated with qPCR in the WT strain, the expression levels were not reported and it is thus not known if resistance from this gene is due to mutation in an expression regulator gene in the clinical *Stenotrophomonas* isolate [13].

***High* ARG-MOB examples**

ARG-MOB=0.95; 3003013 (*target replacement*) *dfrA15* (trimethoprim resistant dihydrofolate reductase dfr): A Class 1 integron with *dfrA15* is widespread in *Vibrio cholera* isolates in Africa, causing resistance to trimethoprim. It was furthermore found on a conjugative plasmid [14]. In this study, it is the ARO with the highest risk, due to the fact that it was only found to be associated with IS elements, integrons and plasmids (all ratios = 1). It has a Simpson index of 0.82 and the 7 CRLs are dispersed across 6 genera (*Vibrio*, *Salmonella*, *Enterobacter*, *Leclercia*, *Klebsiella*, and *Escherichia*).

ARG-MOB=0.91; 3002271 (*inactivation*) VIM-1 (VIM beta-lactamase): VIM-1 was originally isolated from a multiresistant *E. coli* from a patient in Greece. It was inserted in a class 1 integron and found on a conjugative plasmid [15]. It has since been seen in multiple *Enterobacteriaceae*, typically in association with integrons and plasmids, and is globally spread. Here, it scores the fourth-highest ARG-MOB= of 0.91 with IS, replicon, and integron ratios of 0.95, 0.95, and 1, respectively. The 21 CRLs are found in 6 distinct genera (*Pseudomonas*, *Salmonella*, *Escherichia*, *Klebsiella*, *Citrobacter*, and *Enterobacter*).

ARG-MOB=0.89; 3004635 (*inactivation*) AAC(6'): During an *Enterobacter* outbreak in Venezuela, the multi-resistance encoding conjugative plasmid pBWH301 was isolated. Amongst other ARGs, *aacA7* was found to encode AAC(6')-I aminoglycoside acetyltransferase in an integron [16]. In RefSeq complete genomes, there are 12 CRLs which are all on plasmids and associated with IS elements. Furthermore, it is inserted in integrons in 92% of cases. It is dispersed across 3 genera for a Simpson index of 0.63. Several other AROs for AAC(6') subtypes are among the highest ARG-MOB scoring AROs, resulting in one of the highest mean ARG-MOB scores for any Antibiotic Inactivation submechanism.

ARG-MOB=0.86; 3002847 (*inactivation*) rifampin ADP-ribosyltransferase (Arr): From a multi-resistant clinical *P. aeruginosa* isolate, a class I integron was cloned into an expression vector and transformed into *E. coli* to screen for rifampin resistance [17]. Although an expression vector was used to identify rifampin resistance genes under heterologous expression, it was described that the DNA insert of the clone carrying rifampin resistance gene *arr-2* also carried a class I integron with *arr-2* inserted as a gene cassette. This corroborates our finding that *arr-2* is a high risk putative ARG where it was associated with IS elements in 94% of CRLs, found on plasmids on 87%, and found as integron gene cassettes in 96% of CRLs (n=54). Furthermore, it was found in 8 distinct genera, for a Simpson index of 0.64 (*Acinetobacter*, *Citrobacter*, *Escherichia*, *Klebsiella*, *Proteus*, *Pseudomonas*, *Salmonella*, and *Shewanella*).

Supplementary References

1. Tansirichaiya S, Rahman MA, Roberts AP. The Transposon Registry. *Mob DNA*. 2019; doi: 10.1186/s13100-019-0182-3.

2. Li J, Zhang H, Ning J, Sajid A, Cheng G, Yuan Z, et al.. The nature and epidemiology of OqxAB, a multidrug efflux pump. *Antimicrob Resist Infect Control*. 2019; doi: 10.1186/s13756-019-0489-3.

3. Perez F, Rudin SD, Marshall SH, Coakley P, Chen L, Kreiswirth BN, et al.. OqxAB, a quinolone and olaquindox efflux pump, is widely distributed among multidrug-resistant *Klebsiella pneumoniae* isolates of human origin. *Antimicrob Agents Chemother*. 2013; doi: 10.1128/AAC.00725-13.

4. Yuan J, Xu X, Guo Q, Zhao X, Ye X, Guo Y, et al.. Prevalence of the *oqxAB* gene complex in *Klebsiella pneumoniae* and *Escherichia coli* clinical isolates. *J Antimicrob Chemother*. 2012; doi: 10.1093/jac/dks086.

5. McCoy AJ, Sandlin RC, Maurelli AT. In vitro and in vivo functional activity of *Chlamydia* MurA, a UDP-N-acetylglucosamine enolpyruvyl transferase involved in peptidoglycan synthesis and fosfomycin resistance. *J Bacteriol*. 2003; doi: 10.1128/JB.185.4.1218-1228.2003.

6. Truong-Bolduc QC, Dunman PM, Strahilevitz J, Projan SJ, Hooper DC. MgrA is a multiple regulator of two new efflux pumps in *Staphylococcus aureus*. *J Bacteriol*. 2005; doi: 10.1128/JB.187.7.2395-2405.2005.

7. Cundliffe E. Glycosylation of macrolide antibiotics in extracts of *Streptomyces lividans*. *Antimicrob Agents Chemother*. 1992; doi: 10.1128/AAC.36.2.348.

8. Bador J, Neuwirth C, Grangier N, Muniz M, Germé L, Bonnet J, et al.. Role of AxyZ transcriptional regulator in overproduction of AxyXY-OprZ multidrug efflux system in *Achromobacter* species mutants selected by tobramycin. *Antimicrob Agents Chemother*. 2017; doi: 10.1128/AAC.00290-17.

9. Morita Y, Tomida J, Kawamura Y. Primary mechanisms mediating aminoglycoside resistance in the multidrug-resistant *Pseudomonas aeruginosa* clinical isolate PA7. *Microbiology*. 2012; doi: 10.1099/mic.0.054320-0.

10. Rossolini GM, Franceschini N, Lauretti L, Caravelli B, Riccio ML, Galleni M, et al.. Cloning of a *Chryseobacterium (Flavobacterium) meningosepticum* chromosomal gene (*blaA(CME)*) encoding an extended-spectrum class a β- lactamase related to the Bacteroides cephalosporinases and the VEB-1 and PER β-lactamases. *Antimicrob Agents Chemother*. 1999; doi: 10.1128/aac.43.9.2193.

11. Hegde SS, Vetting MW, Roderick SL, Mitchenall LA, Maxwell A, Takiff HE, et al.. Biochemistry: A fluoroquinolone resistance protein from *Mycobacterium tuberculosis* that mimics DNA. *Science (80-)*. 2005; doi: 10.1126/science.1110699.
12. Poissy J, Aubry A, Fernandez C, Lott MC, Chauffour A, Jarlier V, et al.. Should moxifloxacin be used for the treatment of extensively drug-resistant tuberculosis? An answer from a murine model. *Antimicrob Agents Chemother*. 2010; doi: 10.1128/AAC.00968-10.
13. Okazaki A, Avison MB. Aph(3′)-IIc, an aminoglycoside resistance determinant from *Stenotrophomonas maltophilia*. *Antimicrob Agents Chemother*. 2007; doi: 10.1128/AAC.00795-06.
14. Ceccarelli D, Bani S, Cappuccinelli P, Colombo MM. Prevalence of *aadA1* and *dfrA15* class 1 integron cassettes and SXT circulation in *Vibrio cholerae* O1 isolates from Africa. *J Antimicrob Chemother*. 2006; doi: 10.1093/jac/dkl352.
15. Miriagou V, Tzelepi E, Gianneli D, Tzouvelekis LS. *Escherichia coli* with a self-transferable, multiresistant plasmid coding for metallo-β-lactamase VIM-1. *Antimicrob Agents Chemother*. 2003; doi: 10.1128/AAC.47.1.395-397.2003.
16. Bunny KL, Hall RM, Stokes HW. New mobile gene cassettes containing an aminoglycoside resistance gene, aacA7, and a chloramphenicol resistance gene, catB3, in an integron in pBWH301. *Antimicrob Agents Chemother*. 1995; doi: 10.1128/AAC.39.3.686.
17. Tribuddharat C, Fennewald M. Integron-mediated rifampin resistance in *Pseudomonas aeruginosa*. *Antimicrob Agents Chemother*. 1999; doi: 10.1128/aac.43.4.960.
18. Seemann T. barrnap 0.9 : rapid ribosomal RNA prediction. *https://github.com/tseemann/barrnap*.
